# Prefrontal Transcranial Direct Current Stimulation in Pediatric Attention-Deficit/Hyperactivity Disorder: A Randomized Clinical Trial

**DOI:** 10.1001/jamanetworkopen.2024.60477

**Published:** 2025-02-21

**Authors:** Kerstin Krauel, Hannah Brauer, Carolin Breitling-Ziegler, Christine M. Freitag, Christina Luckhardt, Andreas Mühlherr, Magdalena Schütz, Sara Boxhoorn, Christine Ecker, Miguel Castelo-Branco, Daniela Sousa, Helena C. Pereira, Joana Crisóstomo, Fabienne Schlechter, Isabel Wrachtrup Calzado, Julia Siemann, Vera Moliadze, Maike Splittgerber, Giada Damiani, Ricardo N. Salvador, Giulio Ruffini, Rafal Nowak, Claire Braboszcz, Aureli Soria-Frisch, Axel Thielscher, Alena M. Buyx, Michael Siniatchkin, Astrid Dempfle, Alexander Prehn-Kristensen

**Affiliations:** 1 Department of Child and Adolescent Psychiatry and Psychotherapy, Otto-von-Guericke-University Magdeburg, Magdeburg, Germany; 2 German Center for Mental Health (DZPG), Partner Site Halle-Jena-Magdeburg, Germany; 3 Center for Behavioral Brain Sciences, Magdeburg, Germany; 4 Institute of Child and Adolescent Psychiatry, Centre for Integrative Psychiatry, School of Medicine, University Medical Center Schleswig-Holstein-Campus Kiel, Kiel, Germany; 5 Department of Child and Adolescent Psychiatry, Psychosomatics, and Psychotherapy, University Hospital Frankfurt, Goethe-University Frankfurt am Main, Frankfurt, Germany; 6 Coimbra Institute for Biomedical Imaging and Translational Research, Institute of Nuclear Sciences Applied to Health, University of Coimbra, Coimbra, Portugal; 7 Institute of Physiology, Faculty of Medicine, Coimbra, Portugal; 8 Department of Child and Adolescent Psychiatry and Psychotherapy, Ev. Hospital Bethel, Bielefeld, Germany; 9 Institute of Medical Psychology and Medical Sociology, University Medical Center Schleswig Holstein, Kiel University, Kiel, Germany; 10 Neuroelectrics, Barcelona, Spain; 11 Starlab, Barcelona, Spain; 12 Danish Research Centre for Magnetic Resonance, Centre for Functional and Diagnostic Imaging and Research, Copenhagen University Hospital Amager and Hvidovre, Hvidovre, Denmark; 13 Technical University of Denmark, Section for Magnetic Resonance, Department of Health Technology, Kongens Lyngby, Denmark; 14 Institute of History and Ethics in Medicine, Technical University of Munich, Munich, Germany; 15 Department of Child and Adolescent Psychiatry, Psychosomatics, and Psychotherapy, Medical Faculty, RWTH Aachen University, Aachen, Germany; 16 Institute of Medical Informatics and Statistics, University Hospital Schleswig Holstein, Kiel University, Kiel, Germany; 17 Department of Psychology, Faculty of Human Sciences, MSH Medical School Hamburg - University of Applied Sciences and Medical University, Hamburg, Germany

## Abstract

**Importance:**

Transcranial direct current stimulation (tDCS) has the potential to be a sustainable treatment option in pediatric attention-deficit/hyperactivity disorder (ADHD), but rigorously controlled multicenter clinical trials using state-of-the-art stimulation techniques are lacking.

**Objectives:**

To determine effect sizes of changes in cognitive and clinical measures and to assess safety and tolerability in the course of optimized multichannel tDCS over prefrontal target regions in pediatric ADHD.

**Design, Setting, and Participants:**

In the sham-controlled, double-blind, parallel-group randomized clinical trial E-StimADHD (Improving Neuropsychological Functions and Clinical Course in Children and Adolescents With ADHD With Anodal Transcranial Direct Current Stimulation of the Prefrontal Cortex: A Randomized, Double-Blind, Sham-Controlled, Parallel Group Trial Using an Uncertified Class IIa Device) with 2 independent study arms, participants were enrolled from September 15, 2018, to August 10, 2021, and follow-up was completed October 4, 2021. Data were analyzed January 26, 2022, to November 8, 2023. The trial was conducted at the departments of child and adolescent psychiatry or pediatrics of 5 university hospitals in Portugal and Germany. Youths 10 to 18 years of age with ADHD according to the *Diagnostic and Statistical Manual of Mental Disorders, Fifth Edition* (all presentations), an IQ of at least 80, and the ability to refrain from stimulant medication during participation in the trial were eligible.

**Interventions:**

Optimized multichannel anodal tDCS or sham stimulation (1 mA, 20 minutes) targeting the left dorsolateral prefrontal cortex (lDLPFC; study A) or the right inferior frontal gyrus (rIFG; study B) was applied in 10 sessions, concurrent with performance on a cognitive target task (study A, working memory assessed in the n-back task; study B, interference control assessed in the flanker task).

**Main Outcomes and Measures:**

Effect sizes for changes in accuracy measures (d-prime or flanker effect) in the target tasks assessed after the intervention. Primary analyses were based on the modified intention-to-treat set.

**Results:**

This study included 69 youths (54 [78%] male) with a median age of 13.3 years (IQR, 11.9-14.9 years). Compared with sham tDCS, verum stimulation of the lDLPFC led to significantly lower working memory accuracy (effect size, −0.43 [95% CI, −0.68 to −0.17]; *P* = .001). Stimulation of the rIFG significantly improved interference control (effect size, 0.30 [95% CI, 0.04-0.56]; *P* = .02). Adverse events were mostly mild and transient and did not occur more often in the verum group. For example, the most frequent adverse events were headache (sham, n = 30; verum, n = 20), nasopharyngitis (sham, n = 11; verum, n = 5), and feeling of electric discharge (sham, n = 5; verum, n = 3).

**Conclusions and Relevance:**

In this randomized clinical trial, prefrontal anodal tDCS induced small- to medium-sized effects in youths with ADHD, with only mild and transient adverse events. The optimized multichannel montage chosen to target the lDLPFC, however, decreased working memory performance. This unexpected finding stresses the importance of incorporating insights from basic research on the mechanisms and preconditions of successful tDCS in future study designs to advance application of tDCS in pediatric ADHD.

**Trial Registration:**

German Clinical Trials Register ID: DRKS00012659

## Key Points

**Question:**

What are the effect sizes of optimized multichannel transcranial direct current stimulation (tDCS) on cognitive and clinical measures in youths with attention-deficit/hyperactivity disorder (ADHD)?

**Findings:**

In this randomized clinical trial involving 69 youths with ADHD, tDCS of the left dorsolateral prefrontal cortex (maximum, 10 sessions) led to significantly lower working memory accuracy as assessed on an n-back task compared with the sham group. Verum stimulation of the right inferior frontal gyrus significantly improved interference control as assessed on a flanker task.

**Meaning:**

These results suggest that targeted tDCS can significantly affect brain regions relevant to ADHD and should be further explored as a treatment option in pediatric ADHD while carefully controlling for cognitive adverse effects.

## Teaser

This randomized clinical trial assesses effect sizes of changes in cognitive and clinical measures following prefrontal multichannel transcranial direct current stimulation vs sham stimulation for attention-deficit/hyperactivity disorder among youths.

## Introduction

Attention-deficit/hyperactivity disorder (ADHD), characterized by developmentally inappropriate levels of inattention, impulsivity, and hyperactivity, is one of the most common childhood-onset psychiatric disorders.^1^ Pharmacological treatment approaches, particularly the use of stimulants, have been shown to be initially effective and safe, but adverse effects or low treatment acceptance frequently lead to discontinuation of treatment.^2,3^ In recent years, the benefit of noninvasive brain stimulation techniques, such as transcranial direct current stimulation (tDCS), has been explored in ADHD. In tDCS, a low-intensity current (0.5-2.0 mA) is induced between anodal and cathodal electrodes mounted on the surface of the scalp.^4^ Repeated stimulation of a target brain region can strengthen functional connectivity in associated networks^5^ and can induce metaplasticity when paired with target-specific activation, for example, through a task.^6,7^ Even though a meta-analysis^8^ suggested small positive effects of tDCS in pediatric ADHD, evidence from double-blind, parallel-group, sham-controlled studies with repeated stimulation (≥5 sessions) and without concurrent stimulant medication is lacking. In the multicenter randomized clinical trial E-StimADHD (Improving Neuropsychological Functions and Clinical Course in Children and Adolescents With ADHD With Anodal Transcranial Direct Current Stimulation of the Prefrontal Cortex: A Randomized, Double-Blind, Sham-Controlled, Parallel Group Trial Using an Uncertified Class IIa Device), we chose the left dorsolateral prefrontal cortex (lDLPFC) and the right inferior frontal gyrus (rIFG) as target regions. Both regions have been targeted in earlier tDCS studies^9,10,11^ in ADHD to improve working memory (WM)^12,13,14^ and interference control.^15,16^ In contrast to previous studies, E-StimADHD used a multichannel montage^17^ optimized to maximize the electric field in the target region while reducing current flow in nontarget areas, which is particularly important in pediatric samples.^18,19^

The primary objectives of E-StimADHD were to investigate effect sizes of changes in task performance and study safety and tolerability of multichannel anodal tDCS targeting the lDLPFC (study A) or the rIFG (study B) in youths with ADHD who were not receiving concurrent stimulant medication. Our secondary objectives included the assessment of clinical effects, namely ADHD symptom load and health-related quality of life, as well as performance in nontarget tasks to investigate cognitive transfer or adverse effects.

## Methods

E-StimADHD was conducted as a multicenter clinical trial using a randomized, sham-controlled, parallel group design; the trial protocol and statistical analysis plan are provided in Supplement 1. Study A and study B were independent 2-arm studies, each consisting of a verum (anodal tDCS plus task) and sham stimulation (sham tDCS plus task) condition with identical inclusion criteria. Patients were included intermittently from September 15, 2018, to August 10, 2021, and follow-up was completed October 4, 2021. The trial was approved by the German and Portuguese national authorities and respective ethics committees (eTable 1 in Supplement 2). Participants and their parents or custodians received detailed written and oral information and provided written informed consent or assent. This randomized clinical trial followed the Consolidated Standards of Reporting Trials (CONSORT) reporting guideline.^20^ In addition to being registered with the German Clinical Trials Register, E-StimADHD is listed with the European Database on Medical Devices.

### Participants

Youths (10 to 18 years of age) with ADHD according to the *Diagnostic and Statistical Manual of Mental Disorders, Fifth Edition* (all presentations), and an IQ of at least 80 were recruited via the departments of child and adolescent psychiatry or pediatrics of 5 university hospitals in Portugal (Coimbra) and Germany (Bielefeld, Frankfurt, Kiel, and Magdeburg). All participants and their parents were interviewed by trained psychologists or child and adolescent psychiatrists using a German or Portuguese adaption of the revised Schedule for Affective Disorders and Schizophrenia for School-Age Children: Present and Lifetime Version.^21^ Nonverbal IQ was assessed at each site. Exclusion criteria were birth weight less than 2500 g; preterm birth (<37th week of pregnancy); past or present neurological disease or brain surgery; dermatologic diseases of the scalp; all comorbid psychiatric disorders other than oppositional defiant disorder, conduct disorder, elimination disorders, anxiety disorders, and learning disorders; history of craniocerebral injury with loss of consciousness; heart disease; pregnancy; concurrent neurofeedback therapy; concurrent pharmacological treatment of ADHD; or participation in another clinical study. Stimulant treatment had to be omitted 2 weeks before the baseline assessment and during the study. Youths or their families were reimbursed for their participation in all visits except those with intervention. Time between screening, baseline assessment, and start of the intervention was maximally 4 weeks each. The postintervention assessment took place within 1 week after the last intervention visit, and there was a follow-up assessment within 4 to 5 weeks. Details on screening procedures and sample size calculations are provided in eMethods 1 and 2 in Supplement 2.

### Intervention

Stimulation was administered using the hybrid electroencephalogram (EEG) and tDCS 32-channel neurostimulator Starstim32 (Neuroelectrics), a noncertified class IIa device. Prior to the clinical trial, optimized montages of electrodes with a total injected current strength of 1 mA were determined for each target region (eMethods 3 in Supplement 2) and used for all participants accordingly (eMethods 3 in Supplement 2). Circular electrodes (Pistim; 3.14 cm^2^) filled with EEG electrode gel were positioned using a head cap following the 10-10 system. For stimulation of the lDLPFC, anodal electrodes were positioned at AF3 and F3 (0.5 mA) and cathodal electrodes at TP7 and Oz (−0.5 mA). To stimulate the rIFG, anodal electrodes were positioned at F6 and F8 (0.5 mA) and cathodal electrodes at AFZ and P7 (−0.5 mA). There was a ramping period of 30 seconds at the beginning and at the end of stimulation. To facilitate blinding in the sham condition, the electric current was also ramped up and down at the beginning and the end of the session and additionally maintained for 5 seconds at the beginning of the session. Verum or sham tDCS was applied by trained staff (H.B., C.B.-Z., A.M., M. Schütz, S.B., D.S., H.C.P., J.C., F.S., I.W.C., and J.S.) for 20 minutes per weekday for 2 weeks (2 times 5 sessions). For missing sessions, replacement visits could be scheduled in a third week. During tDCS, patients performed either a WM task (target task study A: visual 2-back) or a cognitive interference task (target task study B: flanker) (eMethods 4 in Supplement 2).

### Randomization and Blinding

Participants were allocated randomly to 1 of the 2 studies (study A or study B) and treatment conditions (verum or sham). The allocation ratio to verum tDCS and sham stimulation was 1:1. Randomization was stratified by center and sex (details of sequence generation and allocation concealment are provided in eMethods 5 in Supplement 2). Patients, parents or legal caregivers, and personnel involved in administering the intervention or assessing outcomes were blinded to the stimulation condition (verum vs sham) for the whole duration of the trial (eMethods 6 in Supplement 2).

### Outcomes

Outcome measures of cognitive task performance were determined for the respective target task and 2 nontarget tasks. The target tasks of study A (n-back) and study B (flanker task) were defined as nontarget tasks in the respective other study. In both studies, the nontarget task continuous performance task A-X^22^ assessed effects on attentional and inhibitory control. Target tasks were assessed at baseline, each intervention visit, the postintervention visit, and within 4 to 5 weeks after the last stimulation session (follow-up visit). Nontarget tasks were assessed at baseline, the postintervention visit, and follow-up visit (eFigure 1 in Supplement 2).

The primary outcome was cognitive task performance at the postintervention visit, measured in study A by d-prime, which captures overall accuracy in the working memory task (d-prime = *z*-transformed rate of correct reactions to a target [hit rate] minus *z*-transformed rate of incorrect reactions to a nontarget [false alarm]). In study B, the primary outcome at the postintervention visit was the flanker effect (percentage of correct responses in incongruent trials minus percentage of correct responses in congruent trials) providing a measure of overall accuracy in cognitive interference control. Here, a more negative value indicated a higher proneness to interference.

The ADHD rating scale was used to assess ADHD symptom load^23^ at baseline and at the postintervention and follow-up visits. Participants’ health-related quality of life was measured via self-rating and parent or custodian rating^24^ at screening and follow-up.

### Safety and Tolerability

All sites used the questionnaire of sensations related to transcranial electrical stimulation to assess discomfort during and after the stimulation.^25^ Itching, pain, burning, warmth, metal taste, and fatigue were rated on an ordinal scale (0 = none, 1 = mild, 2 = moderate, and 3 = strong). Adverse events (AEs) were assessed at each visit after screening.

### Statistical Analysis

The primary analysis was based on the modified intention-to-treat set, including all randomized patients irrespective of the amount of treatment actually received or adherence to the intervention but excluding patients who did not start the intervention, that is, did not participate in any stimulation session (eMethods 7 in Supplement 2). Differences between verum and sham conditions at the postintervention and follow-up visits for the continuous primary and secondary outcomes were estimated from a linear mixed-effects model with the covariates center, sex, age, IQ, and the value of the respective outcome variable at screening or baseline. All available outcome values at all time points after randomization were used in this model without imputation of missing values (eMethods 7 in Supplement 2). Estimated marginal means (emmeans) for the original scale of assessment (presented are values for male sex and sample means of age, IQ, and baseline values of the outcome measure) and standardized effect sizes for the difference with 95% CIs were calculated for postintervention assessment or follow-up. Secondary outcome measures were considered as exploratory and thus were not adjusted for multiplicity.

Harms and tolerability were evaluated aggregating data from both study A and study B to increase statistical precision as no differences between target regions were expected. Tolerability was compared between intervention groups using a linear mixed-effects model to account for up to 10 assessments per participant, adjusted for age, sex, and IQ. Analyses were performed from January 26, 2022, to November 8, 2023, using R, version 4.2.3 (R Project for Statistical Computing). Statistical significance was defined as *P* < .05.

## Results

**Figure 1.**
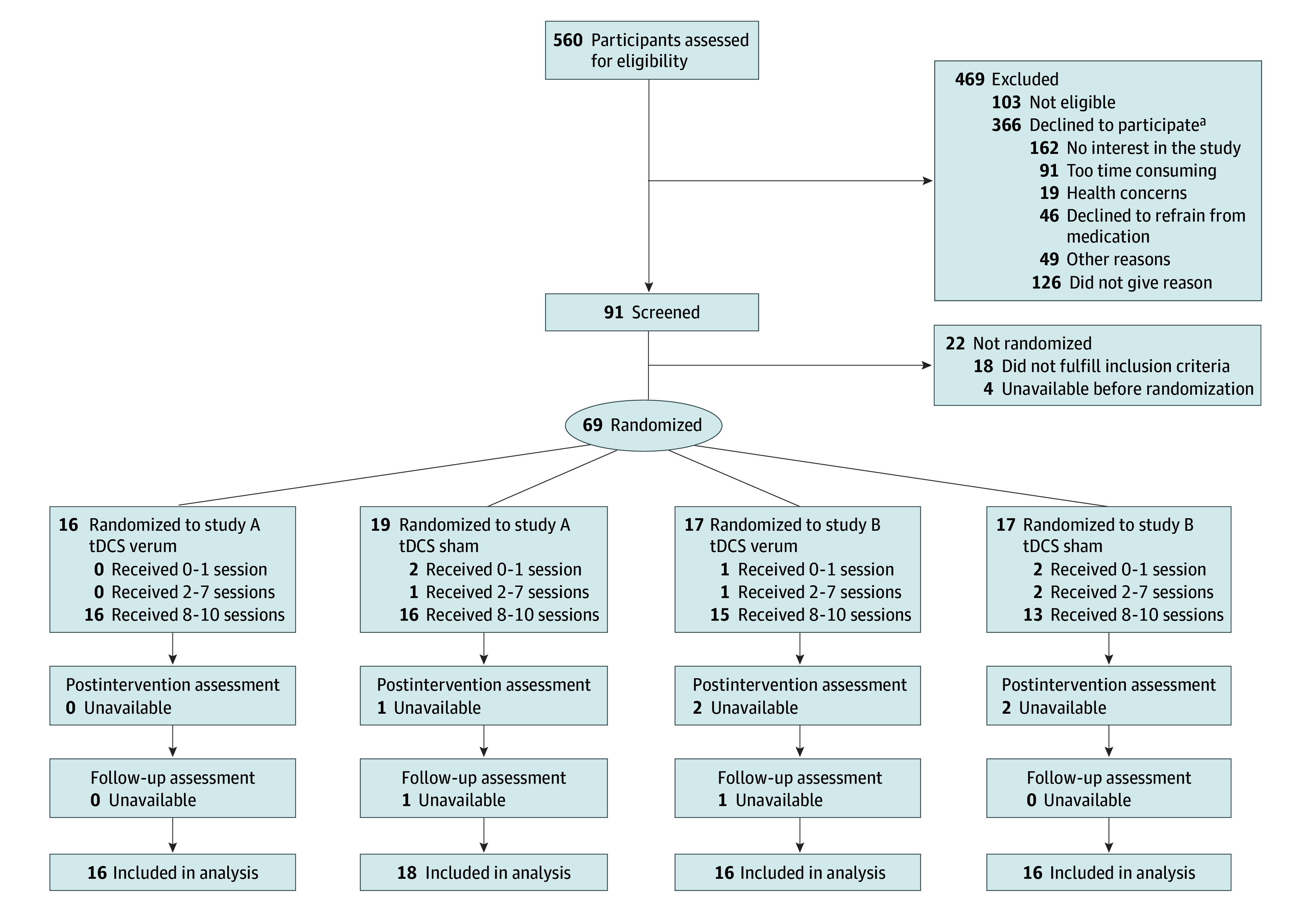
Patient Flow Diagram tDCS represents transcranial direct current stimulation. ^a^Multiple responses were allowed.

Across all study sites, a total of 560 patients were assessed for eligibility via telephone or email. In total, 69 patients (54 [78%] male, 15 [22%] female) with a median age of 13.3 years (IQR, 11.9-14.9 years) met inclusion criteria and were allocated randomly (Figure 1). Participants were included in the modified intention-to-treat analysis if there was at least 1 value available after baseline for the primary outcome. The mean (SD) numbers of sessions after baseline were 11.8 (0.6) for the verum and 11.1 (2.8) for the sham groups in Study A and 11.2 (1.8) for the verum and 10.7 (2.9) for the sham groups in Study B out of 12 possible sessions. Participants were considered unavailable for an assessment if there was no outcome measured at the respective time point, regardless of the number of tDCS sessions received. Due to delays in study conduct and difficulties in recruitment during the COVID-19 pandemic, the planned sample size could not be reached (eResults in Supplement 2). Table 1 provides an overview of the baseline demographics and clinical characteristics of the verum and sham groups in studies A and B (additional sample characteristics are given in eTable 2 in Supplement 2).

**Table 1.** Baseline Demographics and Clinical Characteristics of Verum and Sham Groups for Studies A and B

Characteristic	Participants, No. (%) for study A (n = 35)		Participants, No. (%) for study B (n = 34)	
	Verum (n = 16)	Sham (n = 19)	Verum (n = 17)	Sham (n = 17)
Sex				
Female	3 (19)	5 (26)	3 (18)	4 (24)
Male	13 (81)	14 (74)	14 (82)	13 (76)
Age, median (IQR), y	12.6 (11.7-15.2)	12.5 (11.0-15.4)	13.5 (11.7-14.2)	14.4 (12.2-14.9)
Nonverbal IQ, median (IQR), score	103 (92-115)	104 (94-112)	101 (90-111)	103 (94-115)
ADHD presentation				
Inattentive	9 (56)	12 (63)	7 (41)	6 (35)
Hyperactive or impulsive	1 (6)	0	3 (18)	0
Combined	6 (38)	7 (37)	7 (41)	11 (65)
Comorbidity^a^				
Oppositional defiant disorder	3 (19)	2 (11)	6 (35)	2 (12)
Conduct disorder	0	1 (5)	2 (12)	2 (12)
Anxiety disorders	1 (6)	1 (5)	1 (6)	1 (6)
Elimination disorders	0	1 (5)	1 (6)	0
Stimulant medication				
Naive	7 (44)	9 (47)	3 (18)	3 (18)
Previous	1 (6)	2 (11)	7 (41)	5 (53)
Current^b^	8 (50)	8 (42)	7 (41)	9 (29)

Abbreviation: ADHD, attention-deficit/hyperactivity disorder.

^a^ Multiple comorbidities possible.

^b^ Refrained from medication use during the study.

### Primary Outcomes

**Figure 2.**
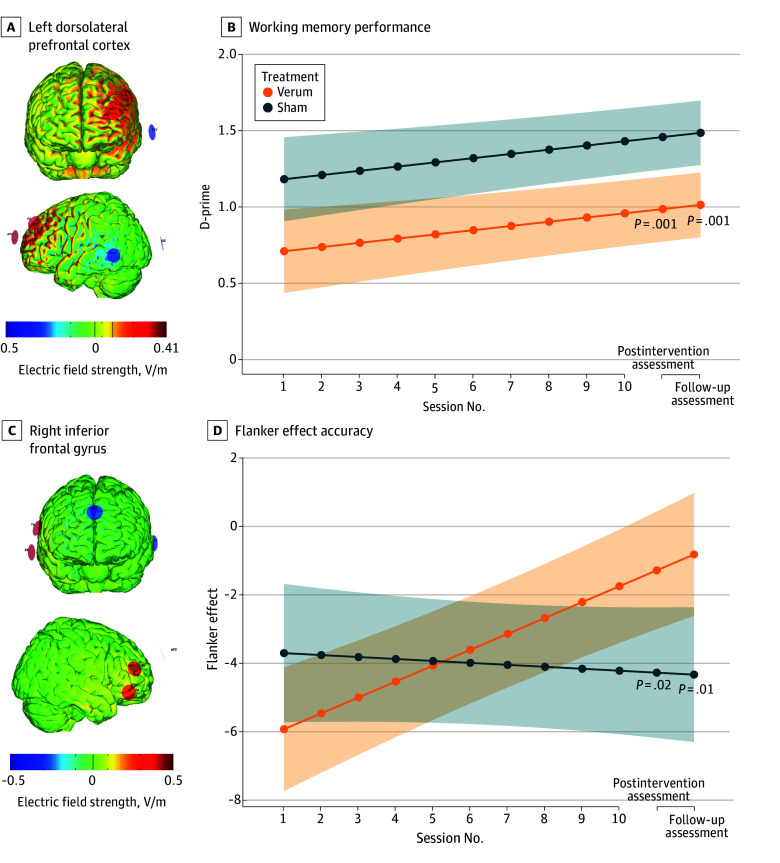
Changes in Primary Outcomes Across Sessions Visualization of the normal component of the electric field for the stimulation montage in study A of the left dorsolateral prefrontal cortex (A) and in study B of the right inferior frontal gyrus (C). Working memory performance (B) and flanker effect accuracy (D) during transcranial direct current stimulation (tDCS) intervention in sessions 1 through 10 and during electroencephalography in the preintervention, postintervention, and follow-up visits. Lines with shaded ribbons indicate estimated marginal means (emmeans) and 95% CIs for emmeans retrieved from a linear mixed-effects model with covariates of center, sex, age, IQ, and the value of the respective outcome at baseline. For d-prime, there was no interaction of time and treatment group. For the flanker effect accuracy, there was an interaction of treatment and time in the model, resulting in different emmeans for the respective time points.

For study A, WM performance assessed via d-prime at the postintervention assessment differed significantly between verum and sham condition, with better performance in the sham group (effect size, −0.43 [95% CI, −0.68 to −0.17]; *P* = .001) (Figure 2; eFigure 2 in Supplement 2). For study B, interference control measured by the flanker effect was significantly higher in the patient group receiving verum stimulation at the postintervention assessment (effect size, 0.30 [95% CI, 0.04-0.56]; *P* = .02) (Figure 2; eFigure 2 in Supplement 2).

### Secondary Outcomes

#### Study A: Cognitive Tasks

The effect of tDCS on d-prime at follow-up was as already mentioned for the postintervention assessment (effect size, −0.43 [95% CI, −0.68 to −0.17]; *P* = .001) (Table 2). For the n-back task, neither hit rate nor reaction time or reaction time variability for hits differed significantly between verum and sham groups at the postintervention or follow-up assessments (Table 2). At the postintervention assessment, continuous performance task accuracy (effect size, 0.21 [95% CI, −0.22 to 0.64]; *P* = .32) and the flanker effect (effect size, 0.23 [95% CI, −0.43 to 0.88]; *P* = .48) did not differ significantly between verum and sham groups (Table 3). Similar results were observed at the follow-up assessment.

**Table 2.** Results of the Target Tasks in Studies A and B, Including Primary and Secondary End Points

Target task and end point	Baseline, verum, and sham, mean (SE)	Postintervention assessment				Follow-up assessment			
		emmean (SE)		Effects		emmean (SE)		Effects	
		Verum	Sham	Effect size (95% CI)^a^	*P* value	Verum	Sham	Effect size (95% CI)^a^	*P* value
**Study A (n-back)**									
Primary end point									
D-prime^b^	0.94 (0.04)	0.99 (0.11)	1.46 (0.11)	−0.43 (−0.68 to −0.17)	.001^c^	1.01 (0.11)	1.49 (0.11)	−0.43 (−0.68 to −0.17)	.001^c^
Secondary end points									
Hit rate %^d^	59.42 (1.19)	41.28 (3.38)	45.20 (3.15)	−0.16 (−0.53 to 0.20)	.38	40.22 (3.61)	45.56 (3.34)	−0.23 (−0.62 to 0.17)	.26
RT hits^b^	601.94 (11.34)	518.17 (30.67)	516.25 (30.05)	0.01 (−0.38 to 0.40)	.96	510.84 (31.88)	508.92 (31.32)	0.01 (−0.38 to 0.40)	.96
SD-RT targets^b^	232.58 (6.24)	195.16 (17.37)	183.66 (15.78)	0.09 (−0.24 to 0.42)	.58	192.46 (17.87)	180.96 (16.30)	0.09 (−0.24 to 0.42)	.58
**Study B (flanker)**									
Primary end point									
Flanker effect accuracy^d^	−14.09 (0.61)	−1.28 (0.87)	−4.27 (0.97)	0.30 (0.04 to 0.56)	.02^c^	−0.82 (0.91)	−4.33 (1.00)	0.36 (0.09 to 0.63)	.01^c^
Secondary end points									
Commission errors (incongruent %)^b^	24.61 (0.75)	8.64 (1.95)	11.12 (2.11)	−0.26 (−0.82 to 0.29)	.36	8.46 (2.00)	10.94 (2.15)	−0.26 (−0.82 to 0.29)	.36
RT incongruent^b^	607.41 (8.97)	568.90 (19.30)	552.84 (20.29)	0.15 (−0.22 to 0.51)	.42	565.48 (20.11)	549.42 (21.06)	0.15 (−0.22 to 0.51)	.42
SD-RT incongruent^d^	256.37 (7.68)	156.95 (13.76)	163.19 (14.89)	−0.05 (−0.39 to 0.28)	.75	157.20 (14.18)	160.71 (15.28)	−0.03 (−0.38 to 0.31)	.86

Abbreviations: emmean, estimated marginal mean; RT, reaction time; SD-RT, standard deviation of reaction time.

^a^ Effect sizes are in favor of the verum group if they are positive for d-prime and flanker effect and negative for reaction times and commission errors.

^b^ Estimates from a model with only main effects of treatment and time (no interaction).

^c^ *P* ≤ .05.

^d^ Estimates from a model with an interaction term between treatment and time (note that emmeans and effect sizes differ for postintervention assessment and follow-up assessment).

**Table 3.** Results of Studies A and B for the ADHD Rating Scale and Nontarget Tasks

Task or variable	Baseline screening, verum and sham, mean (SE)	Postintervention assessment				Follow-up assessment			
		emmean (SE)		Effects		emmean (SE)		Effects	
		Verum	Sham	Effect size (CI)^a^	*P* value	Verum	Sham	Effect size (CI)^a^	*P* value
**Study A**									
ADHD rating scale (sum)									
Inattention^b^	18.41 (0.62)	14.81 (0.99)	14.38 (0.98)	0.12 (−0.61 to 0.84)	.74	13.23 (1.44)	15.05 (1.38)	−0.49 (−1.56 to 0.58)	.35
Hyperactivity^b^	7.29 (0.53)	5.93 (0.70)	5.24 (0.70)	0.26 (−0.46 to 0.97)	.46	4.30 (0.67)	4.85 (0.65)	−0.20 (−0.87 to 0.47)	.54
Impulsivity^b^	6.17 (0.44)	4.72 (0.54)	5.22 (0.54)	−0.24 (−0.96 to 0.48)	.49	3.42 (0.64)	4.95 (0.61)	−0.74 (−1.59 to 0.11)	.07
Total^b^	31.86 (1.33)	25.63 (1.85)	24.73 (1.82)	0.13 (−0.59 to 0.85)	.71	21.02 (2.40)	24.86 (2.31)	−0.55 (−1.51 to 0.41)	.23
Continuous performance task									
Accuracy targets^c^	88.21 (2.22)	89.99 (3.53)	86.45 (3.51)	0.21 (−0.22 to 0.64)	.32	91.43 (2.68)	87.90 (2.59)	0.21 (−0.22 to 0.65)	.32
False alarms %^b^	6.31 (1.50)	4.61 (0.97)	3.02 (0.96)	0.42 (−0.31 to 1.15)	.23	4.25 (0.88)	4.95 (0.84)	−0.19 (−0.83 to 0.45)	.55
RT targets^c^	442.27 (13.30)	481.98 (15.88)	495.28 (16.14)	−0.22 (−0.95 to 0.50)	.53	519.78 (19.55)	533.07 (19.57)	−0.22 (−0.94 to 0.49)	.53
SD-RT targets^c^	153.58 (7.78)	152.69 (14.81)	146.68 (15.04)	0.10 (−0.57 to 0.78)	.76	172.76 (16.39)	166.76 (16.25)	0.10 (−0.57 to 0.77)	.76
Flanker									
Flanker effect accuracy^c^	−12.77 (0.66)	−9.65 (2.81)	−12.12 (2.64)	0.23 (−0.43 to 0.88)	.48	−9.38 (2.60)	−11.84 (2.30)	0.23 (−0.43 to 0.89)	.48
Commission errors incongruent %^c^	33.46 (0.66)	29.05 (2.68)	24.40 (2.69)	0.47 (−0.28 to 1.22)	.20	22.20 (2.96)	17.55 (2.93)	0.47 (−0.28 to 1.22)	.20
RT incongruent^c^	677.92 (11.66)	619.47 (36.39)	627.75 (36.18)	−0.05 (−0.51 to 0.41)	.82	645.02 (31.54)	653.30 (29.61)	−0.05 (−0.52 to 0.42)	.82
SD-RT incongruent^c^	518.79 (44.73)	493.12 (190.27)	450.23 (190.04)	0.04 (−0.07 to 0.15)	.46	293.94 (45.63)	251.05 (41.34)	0.04 (−0.07 to 0.16)	.46
**Study B**									
ADHD rating scale (sum)									
Inattention^b^	17.71 (0.70)	16.26 (1.49)	14.29 (1.47)	0.40 (−0.47 to 1.26)	.34	14.34 (1.78)	14.09 (1.72)	0.05 (−0.96 to 1.06)	.92
Hyperactivity^b^	5.88 (0.48)	4.17 (0.91)	3.99 (0.90)	0.06 (−0.76 to 0.87)	.89	3.17 (0.75)	3.89 (0.72)	−0.23 (−0.89 to 0.43)	.48
Impulsivity^b^	6.47 (0.41)	6.27 (0.74)	4.65 (0.72)	0.66 (−0.22 to 1.53)	.12	5.77 (0.82)	5.43 (0.77)	0.14 (−0.80 to 1.08)	.76
Total^b^	30.06 (1.33)	26.68 (2.60)	22.99 (2.61)	0.42 (−0.45 to 1.29)	.32	23.24 (2.97)	23.47 (2.89)	−0.03 (−1.00 to 0.94)	.96
Continuous performance task									
Accuracy targets^c^	95.39 (0.86)	96.29 (1.68)	92.90 (1.70)	0.44 (−0.01 to 0.89)	.049^d^	96.84 (1.26)	93.45 (1.25)	0.44 (−0.01 to 0.89)	.049^d^
False alarms %^c^	1.24 (0.28)	1.98 (0.70)	1.82 (0.71)	0.05 (−0.49 to 0.59)	.84	1.46 (0.63)	1.30 (0.64)	0.05 (−0.49 to 0.60)	.84
RT targets^c^	466.74 (14.62)	522.90 (26.38)	535.20 (26.65)	−0.12 (−0.78 to 0.54)	.72	542.12 (23.59)	554.42 (23.79)	−0.12 (−0.78 to 0.55)	.72
SD-RT targets^b^	145.90 (7.54)	160.14 (15.38)	184.35 (15.58)	−0.44 (−1.27 to 0.39)	.28	193.45 (12.41)	162.48 (12.31)	0.56 (−0.12 to 1.24)	.08
N-back									
D-prime^c^	1.19 (0.04)	1.66 (0.16)	1.36 (0.16)	0.46 (−0.16 to 1.08)	.13	1.46 (0.15)	1.16 (0.15)	0.46 (−0.16 to 1.09)	.13
Hit rate %^c^	58.29 (0.93)	64.06 (3.86)	52.65 (3.94)	0.75 (0.08 to 1.41)	.02^d^	55.89 (3.82)	44.49 (3.88)	0.75 (0.08 to 1.41)	.02^d^
RT hits^c^	617.87 (11.14)	672.88 (38.07)	718.17 (37.67)	−0.32 (−1.03 to 0.39)	.36	678.91 (37.60)	724.20 (37.02)	−0.32 (−1.03 to 0.39)	.36
SD-RT targets^c^	272.26 (6.44)	219.59 (22.42)	271.45 (22.95)	−0.64 (−1.43 to 0.15)	.10	238.65 (28.98)	290.51 (29.30)	−0.64 (−1.42 to 0.14)	.10

Abbreviations: ADHD, attention-deficit/hyperactivity disorder; emmean, estimated marginal mean; RT, reaction time; SD-RT, standard deviation of reaction time.

^a^ Effect sizes are in favor of the verum arm if they are positive for d-prime, flanker effect, and accuracy and negative for ADHD rating scale scores, reaction times, and commission errors.

^b^ Estimates from a model with an interaction term between treatment and time (note that emmeans and effect sizes differ for postintervention assessment and follow-up assessment).

^c^ Estimates from a model with only main effects of treatment and time (no interaction).

^d^ *P* ≤ .05.

#### Study A: Clinical Measures

Both groups showed lower values on the ADHD rating scales for total ADHD symptoms at the postintervention and follow-up assessments compared with baseline, but there were no significant differential treatment group effects (Table 3). Reduction of impulsivity in the verum stimulation group at follow-up was not statistically significant (effect size, −0.74 [95% CI, −1.59 to 0.11]; *P* = .07). Parent ratings and self-ratings of different domains of health-related quality of life did not differ significantly during follow-up between groups (eTable 3 in Supplement 2).

#### Study B: Cognitive Tasks

At the follow-up assessment, the flanker effect accuracy was significantly higher in the patient group receiving verum stimulation (effect size, 0.36 [95% CI, 0.09-0.63]; *P* = .01) (Table 2). Commission errors as well as reaction time or reaction time variability of incongruent trials in the flanker task did not differ significantly between the sham and verum groups at the postintervention or follow-up assessments. The continuous performance task accuracy for targets favored the verum group at the postintervention assessment (effect size, 0.44 [95% CI, −0.01 to 0.89]; *P* = .049) and at the follow-up assessment (Table 3). In the n-back task, accuracy (hit rate) was increased in the verum group compared with the sham group at the postintervention and follow-up assessments (effect size, 0.75 [95% CI, 0.08-1.41]; *P* = .02 for both assessments).

#### Study B: Clinical Measures

Total ADHD symptoms as assessed using ADHD rating scales were lower in both groups at the postintervention and follow-up assessments compared with baseline but did not significantly differ between verum and sham stimulation (Table 3). There were also no significant differences in parent ratings and self-ratings for the different domains of health-related quality of life (eTable 3 in Supplement 2).

### Safety and Tolerability

Overall, 87 AEs were reported in the sham group and 74 AEs in the verum group (eTable 4 in Supplement 2). Serious adverse events did not occur. All AEs were transient and resolved by the end of the trial. None of the reported AEs were observed at a relevantly higher frequency in the verum group than in the sham group. The most frequent AE in both groups was headache (sham, n = 30; verum, n = 20), followed by nasopharyngitis (sham, n = 11; verum, n = 5) and feeling of electric discharge (sham, n = 5; verum, n = 3).

The overall strength of an itching sensation was mild (emmean verum, 0.87 [95% CI, 0.58-1.16]; emmean sham, 1.04 [95% CI, 0.77-1.31]; effect size, 0.24 [95% CI, −0.24 to 0.72]) with no significant difference between groups (*P* = .33). Reports for all other sensations were also mild and did not differ between intervention conditions (eTable 5 in Supplement 2).

### Blinding Integrity

After the last stimulation visit, participants rated whether they believed that they were in the sham group or verum group. Fisher exact tests revealed no indication that these ratings of the verum group (verum, 61%; sham, 18%; not specified, 21%) and sham group (verum, 50%; sham, 19%; not specified, 31%) were better than random guessing (*P* = .61).

## Discussion

In the present E-StimADHD randomized clinical trial, we aimed to investigate effect sizes of the changes in neuropsychological performance and clinical measures following optimized multichannel anodal tDCS over prefrontal target regions in youths with ADHD. Unfortunately, we did not reach the targeted sample size in the funded period. Thus, the reported effect sizes are less precise than intended but were nevertheless gained within a rigorously controlled trial design. Analyses of the primary outcomes showed that verum stimulation of the lDLPFC led to poorer WM accuracy compared with sham stimulation, whereas verum stimulation of the rIFG significantly improved interference control. Participants’ reports of AEs throughout the trial as well as the standardized assessment of unpleasant sensations at each tDCS session indicated that the application of tDCS with our parameter settings can be considered safe in the observed period and was well tolerated.

The lower WM performance of the verum group in study A was unexpected given that tDCS of the lDLPFC has been successfully used to enhance WM in healthy adults^26^ and certain patient groups (eg, those with schizophrenia^27^) and improved cognitive control in adult and pediatric ADHD.^28,29,30,31^ In adult psychiatric samples,^32,33,34^ tDCS trials often use higher stimulation intensities (2 mA), which could be necessary due to more severe brain pathology or pharmacological treatment. However, tDCS studies in children, adolescents, and healthy adults suggest a nonlinear association between stimulation intensity and effect favoring moderate intensities.^26,35,36,37^ The lower WM performance in our trial could be more likely associated with the placement of the cathodal electrodes over the temporoparietal (TP7) and occipital (Oz) cortex, which is different from common montages^26,38^ and could have interfered with occipital activation necessary for mastering visual WM tasks.^39,40^ Ratings of impulsivity at follow-up, however, were lower in the verum than in the sham group (effect size: −0.74), replicating findings from previous studies.^41^ Possibly, the chosen montage, even though not optimal for enhancing WM performance, successfully modulated altered connectivity patterns of the frontoparietal network with other relevant networks^42,43^ that have been associated with increased hyperactivity or impulsivity.^44^

In study B, optimized multichannel stimulation of the rIFG improved interference control in the verum group and increased accuracy in the WM and continuous performance tasks, supporting the role of the rIFG as an important network hub.^38,45^ Previous studies targeting the rIFG with a conventional bipolar or high-definition montage did not specifically improve interference control but yielded more general positive—but interindividually variable—effects on attentional and response stability^11,46^ or electrophysiological measures^47^ in pediatric ADHD. Our results suggest that the optimized multichannel montage allowed for a more targeted stimulation of the rIFG specifically modulating interference processing and cognitive control.^48^

Clinical effects were less clear. Ratings of ADHD symptoms appeared to decline after the treatment and particularly at follow-up, but this effect was not significantly stronger in the verum group. These findings could suggest different trajectories for (direct) neuropsychological and (indirect) clinical effects. Leffa et al^33^ showed in adult patients with ADHD that beneficial effects of right DLPFC stimulation on self-reported attentional problems did not occur after 14 sessions but only after 28 sessions. Thus, future studies need to identify the optimal number, spacing, and timing of sessions^49,50,51^ required to achieve persistent clinical effects in pediatric ADHD samples.

### Limitations

Our recruitment was impeded for several reasons. First, access to patients was severely compromised during the COVID-19 pandemic due to recurrent shutdowns; infections in patients, parents, or staff; and lockdown measures that affected families with children with ADHD in particular. Second, German state authorities were concerned about the use of electrical stimulation in minors and requested 2 recruitment stops. However, the exchange between authorities and researchers was also fruitful and reduced reservations. Third, in contrast to previous tDCS studies in pediatric ADHD,^52^ we did not allow concurrent stimulant medication. To probe tDCS as a clinical treatment option with prolonged application, the concurrent use of tDCS and stimulant medication needs to be explored, especially since increased availability of dopamine during tDCS can facilitate plasticity-related processes.^53,54^

## Conclusions

Our findings from a double-blind, sham-controlled, multicenter randomized clinical trial suggest that optimized multichannel anodal tDCS over the prefrontal cortex (lDLPFC, rIFG) in unmedicated youths with ADHD was safe and well tolerated in the observed period with our parameter settings and could induce small- to medium-sized effects in cognitive and clinical measures. The optimized multichannel montage chosen to target the lDLPFC, however, decreased working memory performance. To establish tDCS as a therapeutic option in ADHD and minimize (cognitive) adverse effects, the importance of increasing electrical field strength in a target region or network and to exploit the conceptual and methodological advances in the field of tDCS^55,56,57^ should be considered in future pediatric trial designs.

## Notes

Supplement 1.
Trial Protocol and Statistical Analysis Plan

Supplement 2. **eTable 1.** Ethical votes **eTable 2.** Additional sample characteristics **eTable 3.** Results of health related quality of life **eTable 4.** Safety and tolerability **eTable 5.** Unpleasant sensations during stimulation **eFigure 1**. Procedure **eFigure 2.** Raw means for working memory performance (d-prime) and flanker effect accuracy **eMethods 1**. Participants **eMethods 2.** Sample size **eMethods 3.** Optimized montages **eMethods 4.** Cognitive tasks **eMethods 5.** Randomization/sequence generation/allocation concealment **eMethods 6.** Blinding **eMethods 7.** Statistical methods **eResults.** Recruitment

Supplement 3.
Data Sharing Statement
