# Supplementary material for: Prefrontal Transcranial Direct Current Stimulation in Pediatric Attention-Deficit/Hyperactivity Disorder: A Randomized Clinical Trial
Source: JAMA Netw Open. 2025 Feb 21;8(2):e2460477. doi: 10.1001/jamanetworkopen.2024.60477 (PMC11846015; doi:10.1001/jamanetworkopen.2024.60477)
Supplement: Supplement 1. — Trial Protocol and Statistical Analysis Plan [file jamanetwopen-e2460477-s001.pdf]

Protocol

Improving neuropsychological functions and clinical course in children and adolescents with ADHD with anodal transcranial direct current stimulation (tDCS) of the prefrontal cortex: a randomized, double-blind, sham-controlled, parallel group trial using an uncertified class IIa device

E-StimADHD

Protocol Code:

EUDAMED-Nr:

Version: 1.0

Date: 15.08.2017

Sponsor

Dean: Prof. Dr. Hermann-Josef Rothkötter

Otto-von-Guericke University Magdeburg

Medical Faculty

Leipziger Str. 44

39120 Magdeburg

Coordinating Investigator

PD Dr. Kerstin Krauel

Department of Child and Adolescent Psychiatry and Psychotherapy

Otto-von-Guericke University Magdeburg

Medical Faculty

Leipziger Str. 44

39120 Magdeburg

Confidentiality

The information in this protocol is strictly confidential. It is only to be viewed by the investigator, further individuals participating in the conduct of this study, ethics committees and authorities.

This protocol must not be passed on to third parties without the Sponsor's or the coordination investigator's permission.

SIGNATURES

The persons listed here agree to the clinical study as outlined in this protocol by their signature and agree to conduct the clinical study according to this protocol, all applicable national regulations and the principles of Good Clinical Practice.

Sponsor‘s Representative

Prof. Dr. Hermann-Josef Rothkötter

Location, Date	Signature

Coordinating Investigator

PD Dr. Kerstin Krauel

Location, Date	Signature

Statistician

Prof. Dr. Astrid Dempfle

Location, Date	Signature

Table of Contents

SIGNATURES **2**

I. ORGANISATIONAL STRUCTURE 6
II. SYNOPSIS 9
III. FLOW-CHART 13
IV. EXAMINATION PLAN 14
V. LIST OF ABBREVIATIONS 15

1 INTRODUCTION **18**

1.1 BACKGROUND 18
1.2 RISK-BENEFIT RATIO 19
1.2.1 Risk-benefit ratio concerning the medical device *19*
1.2.2 Risk-benefit ratio concerning the clinical study *20*

2 OBJECTIVES **22**

2.1 STUDY OBJECTIVES 22
2.1.1 Primary objectives *22*
2.1.2 Secondary objectives *22*
2.2 ENDPOINTS 22
2.2.1 Primary endpoints *22*
2.2.2 Secondary endpoints *22*
2.3 SUBSTUDIES 23

3 STUDY DESIGN **23**

4 SCHEDULES (TIMELINES) **23**

5 SITES AND SUBJECTS **24**

5.1 SELECTION OF SITES 24
5.2 SELECTION OF PARTICIPANTS 24
5.2.1 Inclusion criteria *24*
5.2.2 Exclusion criteria *24*
5.2.3 Concomitant medication and therapy *24*
5.3 RECRUITMENT MEASURES 25

6 MEDICAL DEVICE **25**

6.1 DEVICE DESCRIPTION 25
6.2 COMPONENTS OF THE DEVICE 26
6.3 MANUFACTURER 27
6.4 SERIAL NUMBER OR BATCH LABEL 27
6.5 STORAGE, SHIPPING AND RETURN AS WELL AS DOCUMENTATION OF MEDICAL DEVICES (DEVICE ACCOUNTABILITY) 27
6.5.1 Storage *27*
6.5.2 Packaging *28*
6.5.3 Documentation *28*
6.5.4 Return *28*
6.6 Directions for use of medical devices *28*

7 STUDY PROCESS **29**

7.1 STUDY ENROLLMENT 29
7.1.1 Assignment to study arm / randomisation *29*
7.1.2 Blinding *29*
7.1.3 Unblinding *29*
7.2 VISITS 29
7.3 STUDY RELATED PROCEDURES 31
7.4 END OF STUDY AND TERMINATION 34
7.4.1 Regular end of study *34*
7.4.2 Premature end of study *34*
7.4.2.1 Withdrawal of study subject 34

7.4.2.2 Termination of study at a study site ... 35
7.5 Continued treatment and medical care of subject after end of study ... 35

8 EFFICACY AND SAFETY ... 35

8.1 Assessment of efficacy ... 35
8.2 Assessment of safety ... 35
8.3 Adverse events ... 36
8.3.1 Adverse event ... 36
8.3.1.1 Documentation and evaluation ... 36
8.3.1.2 Reporting pathways, responsibilities and deadlines ... 37
8.3.2 Serious adverse events ... 37
8.3.2.1 Documentation and evaluation ... 37
8.3.2.2 Reporting pathways, responsibilities and deadlines ... 37
8.4 Effects ... 37
8.4.1 Adverse effects of medical devices ... 37
8.4.2 Serious adverse device effect (SADE) ... 38
8.4.2.1 Reporting pathways, responsibilities and deadlines ... 38
8.4.3 Incidence ... 38
8.5 Medical history (concomitant diseases) ... 38
8.6 Handling of pregnancies ... 38
8.7 Measures for protection against imminent danger ... 38
8.8 Emergency measures ... 39
8.9 Data safety monitoring board (DSMB) ... 39
8.10 Other possible study related complications and/or risks ... 39

9 DOCUMENTATION ... 39

9.1 Trial master file (TMF) ... 39
9.2 Investigator site file (ISF) ... 40
9.3 Documentation of study data ... 40
9.3.1 Source documents ... 40
9.3.2 Case report form (CRF) ... 40
9.4 Data management ... 40
9.5 Archiving ... 41

10 QUALITY ASSURANCE ... 41

10.1 SOPs ... 41
10.2 Protocol deviations ... 42
10.3 Monitoring ... 42
10.4 Audits and inspections ... 42

11 STATISTICS ... 42

11.1 Sample size calculation ... 42
11.2 Randomisation ... 43
11.3 Statistical methods ... 43
11.3.1 Objectives ... 43
11.3.2 Analysis population ... 43
11.3.3 Data analysis ... 43
11.3.4 Interim analysis ... 44

12 REPORTS AND PUBLICATIONS ... 44

12.1 Reports ... 44
12.1.1 Safety reports ... 44
12.1.2 Final report ... 44
12.2 Publications ... 44

13 ETHICAL, LEGAL AND ADMINISTRATIVE ASPECTS ... 44

13.1 Ethics committees and authorities ... 44
13.2 Insurance ... 44
13.3 Informed consent ... 45

13.4 Data protection 45
13.5 Study protocol 45
13.5.1 Adherence 45
13.5.2 Amendments 45
13.6 Responsibilities 45
13.7 Study registration 46

14 FINANCING 46

15 LITERATURE 47

I. Organisational Structure

Sponsor (Representative)

Institution: Medical Faculty, Otto-von-Guericke-University Magdeburg

Represented by the Dean Prof. Dr. H.-J. Rothkötter

Represented by the Koordinierungszentrum für Klinische Studien (KKS) Magdeburg, Martina Beckmann

Address: Leipziger Str. 44, 39120 Magdeburg, Germany

Tel.: +49 (0) 391 6724780

Fax: +49 (0) 391 6715898

E-mail: martina.beckmann@med.ovgu.de

Coordinating Investigator

Name: PD Dr. Kerstin Krauel

Institution: Department of Child and Adolescent Psychiatry and Psychotherapy, Otto-von-Guericke-University Magdeburg

Address: Leipziger Str. 44, 39120 Magdeburg, Germany

Tel.: +49 (0) 391 6717003

Fax: +49 (0) 391 6717001

E-mail: kerstin.krauel@med.ovgu.de

Statistician

Name: Prof. Dr. Astrid Dempfle

Institution:Institute of Medical Informatics and Statistics, Christian-Albrechts-University Kiel

Address: Brunswiker Str. 10, 24105 Kiel, Germany

Tel.: +49 (0) 431 500 30705

Fax: +49 (0) 431 500 30704

E-mail: dempfle@medinfo.uni-kiel.de

Monitoring

Name: Tina Hagen-Hurley

Institution: Zentrum für Klinische Studien (ZKS) Kiel

Address: Brunswiker Str. 10, 24105 Kiel, Germany

Tel.: +49 (0) 431 50030754

Fax: +49 (0) 431 50030752

E-mail: hagen-Hurley@zks.uni-kiel.de

Data management

Name: Juliane Steiner

Institution: Zentrum für Klinische Studien (ZKS) Kiel

Address: Brunswiker Str. 10, 24105 Kiel, Germany

Tel.: +49 (0) 431 50030757

Fax: +49 (0) 431 50030752

E-mail: steiner@zks.uni-kiel.de

Vigilance

Name: Koordinierungszentrum für Klinische Studien (KKS) Magdeburg, Martina Beckmann

Institution: Medical Faculty, Otto-von-Guericke-University Magdeburg

Address: Leipziger Str. 44, 39120 Magdeburg, Germany

Tel.: +49 (0) 391 6724780

Fax: +49 (0) 391 6715898

E-mail: martina.beckmann@med.ovgu.de

Manufacturer of the Medical Device

Name: Neuroelectrics, contact: Dr. Rafal Nowak

Address: Avenida Tibidabo, 47 bis
08035 Barcelona, Spain

Tel: ++34 93 254 03 68

Fax: ++34 93 212 64 45

Email: info@neuroelectrics.com

Sites

1) Institution: ICNAS, Clinical and Academic Center, University of Coimbra

Address: Paco das Escolas, 3001 451 Coimbra, Portugal

Tel.: ++351 93 6360050

Fax: ++351 239833875

E-mail: mcbranco@fmed.uc.pt

Investigator: Prof. Dr. Miguel Branco

2) Institution: Department of Child and Adolescent Psychiatry, Psychosomatics, and Psychotherapy, Goethe-University Frankfurt am Main

Address: Theodor W. Adorno Platz 1, 60629 Frankfurt am Main, Germany

Tel.: ++49 (0) 69 6301 5408

Fax: ++49 (0) 69 6301 5487

E-mail: c.freitag@em.uni-frankfurt.de

Investigator: Prof. Dr. Christine Freitag

3) Institution: Institute of Medical Psychology and Medical Sociology, Christian-Albrechts-University Kiel

Address: Preußerstr. 1-9, 24105 Kiel, Germany

Tel.: ++49 (0) 431 50030801

Fax.:++49 (0)431 50030804

E-mail: siniatchkin@med-psych.uni-kiel.de

Investigator: Prof. Dr. Michael Siniatchkin

4) Institution: Department of Child and Adolescent Psychiatry, Center for Integrative Psychiatry Kiel

Address: Niemannsweg 147, 24105 Kiel, Germany

Tel.: ++49 (0) 431 50098316

Fax.:++49 (0) 431 50098342

E-mail: a.prehn@zip-kiel.de

Investigator: PD Dr. Alexander Prehn-Kristensen

5) Institution: Department of Child and Adolescent Psychiatry and Psychotherapy, Otto-von-Guericke-University Magdeburg

Address: Leipziger Str. 44, 39120 Magdeburg, Germany

Tel.: ++49 (0)391 6717003

Fax: ++49 (0) 391 6717001

E-mail: kerstin.krauel@med.ovgu.de

Investigator: PD Dr. Kerstin Krauel

Data Safety and Monitoring Board

1) Name: Prof. Dr. med. Michael Nitsche

Institution: Psychology and Neurosciences, Leibniz Research Centre for Working Environment and Human Factors at the Technical University Dortmund

Address: Ardeystraße 67, 44139 Dortmund, Germany

Tel.: +49 (0) 231 1084 301

Fax: +49 (0) 231 1084 340

E-mail: nitsche@ifado.de

2) Name: Prof. Dr. med. Christian Plewnia

Institution: Head of the Group Neurophysiology & Interventional Neuropsychiatry, University Clinic of Psychiatry and Psychotherapy, Eberhard-Karls-University Tübingen

Address: Calwerstr. 14, 72076 Tübingen, Germany

Tel.: +49 (0) 7071 29 86121

Fax: +49 (0) 7071 29 5904

E-mail: christian.plewnia@uni-tuebingen.de

3) Name: Prof. Dr. André Scherag (Statistican)

Institution: Center for Sepsis Control and Care (CSCC), Friedrich-Schiller-University Jena

Address: Erlanger Allee 101, 07747 Jena, Germany

Tel.: +49 (0) 3641 9396692

Fax: +49 (0) 3641 9323379

E-mail: andre.scherag@med.uni-jena.de

II. Synopsis

**Sponsor**	University of Magdeburg, Medical Faculty
**Coordinating Investigator**	PD Dr. Kerstin Krauel
**Title**	Improving neuropsychological functions and clinical course in children and adolescents with ADHD with anodal transcranial direct current stimulation (tDCS) of the prefrontal cortex: a randomized, double-blind, sham-controlled, parallel group trial using an uncertified class IIa device
**Short title**	E-StimADHD
**Indication**	Child and Adolescent Psychiatry, attention deficit hyperactivity disorder
**Study Design**	Randomized, double-blind, sham-controlled, parallel group clinical trial
**Number of Subjects**	**Study A (N = 100)**<br>Arm A (stimulation): 50 Arm B (sham): 50<br>**Study B (N = 100)**<br>Arm A (stimulation): 50 Arm B (sham): 50
**Number of Sites**	5
**Objectives**	***Primary Objectives***<br>• Investigate effect sizes of changes in neuropsychological performance and study safety and tolerability of multi-channel anodal tDCS targeting the left dorsolateral prefrontal cortex (dlPFC) **(Study A)** or the right inferior frontal gyrus (rIFG) **(Study B)** in children and adolescents with ADHD<br>***Secondary Objectives***<br>• Investigate the potential benefit of multi-channel anodal tDCS targeting the left dlPFC **(Study A)** or the rIFG **(Study B)** on behavior and quality of life in children and adolescents with ADHD<br>• Identify individual factors derived from MRI and/or EEG measures as well as clinical measures to predict responsivity to stimulation<br>• Assess expectations and concerns of parents and participants towards tDCS
**Endpoints**	***Primary Endpoints***<br>• **Study A:** Overall accuracy in an *n-back* task (assessing working memory)<br>• **Study B:** Overall accuracy in a *Flanker* task (assessing inhibitory/interference control)<br>• **Study A/B:** Ratings and observations of safety and tolerability aspects at all visits (1-10)

	Secondary Endpoints<br>• Severity of ADHD symptoms (inattention, hyperactivity/ impulsivity) *(Questionnaire)*<br>• **Study A:** Reaction time and reaction time variability in an n-back task, number of omission and commission errors<br>**Study B:** Reaction time and reaction time variability in a Flanker task, number of omission and commission errors in congruent and incongruent trials<br>• Performance measures in neuropsychological tasks that assess impaired functions in ADHD but are different from the task presented during stimulation: accuracy and reaction times / reaction time variability in<br>**Study A:** (1) a Flanker task and (2) a Continuous Performance task (CPT)<br>**Study B:** (1) an n-back working memory task and (2) a Continuous Performance task (CPT)<br>• Functional brain related outcome measures (1) *resting state EEG*: connectivity, phase coherence, Granger causality (2) *event related potentials*: amplitude, latency, latency variability, neuronal sources of task relevant components (3) *resting state fMRI*<br>• Quality of life *(Questionnaire)*
**Key Inclusion Criteria**	• ADHD according to DSM-5 (all subtypes)<br>• age 10 to 18<br>• participant and their parents/custodians are able and willing to give written informed assent/consent
**Key Exclusion Criteria**	• IQ < 80<br>• Birth weight < 2500 gr.<br>• Born before the 37th week of pregnancy<br>• Past or present neurological diseases/brain surgery<br>• Dermatological diseases of the scalp<br>• All psychiatric comorbid disorders other than oppositional defiant disorder, conduct disorder, elimination disorders, anxiety disorders, learning disorders<br>• Cochlear implants<br>• History of craniocerebral injury with loss of consciousness<br>• Heart disease<br>• Pregnancy<br>• Concurrent neurofeedback therapy<br>• Concurrent pharmacological treatment of ADHD<br>• Participation in another clinical study
**Medical Device**	• Wireless hybrid EEG/tCS 32-channel neurostimulator, class IIa device according to the classification in the Council Directive 93/42/CEE for medical devices<br>• Trade name: StarStim32<br>• Manufacturer: Neuroelectrics<br>• Type of application: 1mA, 20 min

<table>
<tr><td>Intervention</td><td>

Real stimulation (tDCS)
Control intervention: sham stimulation

</td></tr>
<tr><td>Visits</td><td>
Screening

Information of participants and their parent and confirmation of consent/assent
Assessment of inclusion and exclusion criteria (medical and psychosocial history, clinical interview and questionnaire, intelligence, pregnancy, MRI criteria)
Assessment of participants' characteristics (handedness, social economic status, ADHD rating scale, puberty status)
quality of life, expectancies towards treatment

Visit 0 (Baseline, within 4 weeks after screening)

Assessment of arousal, mood and motivation before and after the session
64-channel EEG: resting state, neuropsychological performance (tasks: n-back, Flanker, CPT)

Visit 0_MRI (if participants are eligible for MRI)

Assessment of arousal, mood and motivation before and after the session
Structural MRI (T1, T2), DTI, resting state fMRI

Visit 1-10 (Intervention, V1 within 4 weeks after V0 /V0_MRI)

Assessment of arousal, mood and motivation before and after the session
Multichannel stimulation (sham/real) of the left dlPFC during an n-back task (Study A) or rIFG during a Flanker task (Study B)
EEG before and after the multichannel stimulation
Assessment of adverse events in the course of the session (safety questionnaire)

Visit 11 (Post assessment, within 7 days after V10)

Assessment of arousal, mood and motivation before and after the session
64-channel EEG: resting state, neuropsychological performance (tasks: n-back, Flanker, CPT)
ADHD rating scale

Visit 11_MRI (if participants are eligible for MRI)

Assessment of arousal, mood and motivation before and after the session
resting state fMRI

Visit 12 (Follow-up, within 4 to 5 weeks after V10)

Assessment of arousal, mood and motivation before and after the session
64-channel EEG: resting state, neuropsychological performance (tasks: n-back, Flanker, CPT)
ADHD rating scale, quality of life, expectancies towards treatment

</td></tr>
</table>

**Statistical Methods**	• Test for a difference between stimulation and sham arm in the primary outcome measure in **Study A** the overall working memory accuracy (n-back task) and in **Study B** the overall accuracy in inhibitory/ interference control (Flanker task) at T3 using an analysis of covariance with covariates center, sex, puberty status, age, IQ, and the baseline value of accuracy in the respective tasks at T2.<br>• Secondary outcomes and the outcomes at the follow-up assessment (T4) will be analyzed in a similar way.<br>• Emphasis will be on the 95% confidence intervals of the effect size estimates; additionally, p-values will be calculated.<br>• Primary analysis based on the intention-to-treat (ITT) set, including all randomized patients irrespective of the amount of treatment actually received, per-protocol (PP) analysis as sensitivity analysis. All participants will be asked to participate in post and follow-up assessment, even if they drop out of treatment, to minimize the number of those who are lost to follow-up.<br>• In the primary analysis, multiple imputation is used to account for missing data. Sensitivity analyses will be performed to investigate the potential impact of missing data, in particular, by using data of both the post and follow-up assessments in a mixed-effects model repeated measures analysis, and a complete case analysis. There will be no interim analysis.
**Timelines**	• Planned recruitment period: Q3/2017 to Q3/2019<br>• Planned treatment period (LPI to LPO): Q3/2017 to Q3/2019<br>• Planned follow-up period: Q3/2017 to Q3/2019<br>• Duration of intervention per subject: 10 visits within two weeks (in case of illness within three weeks)<br>• Follow-up period per subject: 4 to 5 weeks after V10
**Study Related Procedures**	• Medical history interview<br>• Urine pregnancy test<br>• Clinical interview (K-SADS-PL)<br>• Social Communication Questionnaire (SCQ)<br>• Intelligence (CFT-20 R, performance IQ WISC-III)<br>• Edinburgh Handedness Inventory<br>• Pubertal Development Scale<br>• ADHD rating scale<br>• Quality of life questionnaire (KIDSCREEN-27)<br>• Expectations and concerns towards tDCS<br>• Safety questionnaire<br>• Assessment of arousal, mood and motivation (Self Assessment Manikin, Visual Analogue Scale)<br>• Structural MRI, resting state fMRI, DTI<br>• 64-channel EEG<br>• Neuropsychological tasks (n-back, Flanker, continuous performance)<br>• tDCS stimulation or sham treatment<br>• EEG before and after the multichannel stimulation

III. Flow-Chart

Screening (T1)
Informed consent
Inclusion & exclusion criteria, clinical characterization

↓

Baseline (T2)
structural MRI, resting state fMRI, DTI
64-channel EEG, neuropsychological performance

↓

Allocation to Study A or B; randomization (sham vs. real)

↓

Intervention

**Study A (dlPFC)**		**Study B (rIFG)**	
↓	↓	↓	↓
5 x sham	5 x real	5 x sham	5 x real
2-days break			
5 x sham	5 x real	5 x sham	5 x real

↓

Post (T3)
resting state fMRI
64-channel EEG, neuropsychological performance

↓

Follow up (T4)
64-channel EEG, neuropsychological performance

IV. Examination plan

	T1 Scree-ning	T2 (Baseline)		Intervention										T3 (Post)		T4 (Follow-up)
		V0	V0_MRI	V1	V2	V3	V4	V5	V6	V7	V8	V9	V10	V11	V11_MRI	V12
**Timeline**	V0 within 4 weeks of screening		V1 within 4 weeks of V0	V1 to V5 and V6 to V10 in five consecutive days (V5 to V6: 2 days break)**										V11 within 7 days of V10	V12 within 4 weeks of V11	
Informed consent/assent	x															
Assessment of inclusion/exclusion criteria (incl. MRI questionnaire, pregnancy test)	x															
Medical and psychosocial history (parent)	x															
Pregnancy test	x															
Clinical interview (K-SADS-Pl, parent, child)	x															
Social Communication Questionnaire (parent)	x															
IQ (nonverbal) (child)	x															
Social economic status (parent)	x															
Pubertal Developmental Scale (parent, child)	x															
Edinburgh Handedness Inventory (child)	x															
ADHD Rating Scale (parent)	x													x		x
Quality of life (Kidscreen27; parent, child)	x															x
Expectations towards tDCS (parent, child)	x															x
Structural MRI (T1, T2 weighted)			x*													
Resting state fMRI			x*												x*	
DTI			x*													
Assessment of arousal/motivation		x	x*	x	x	x	x	x	x	x	x	x	x	x	x*	x
64-channel EEG (resting state)		x												x		x
64-channel EEG (n-back / Flanker / CPT)		x												x		x
Stimulation (real/sham) + task (Study A: n-back; Study B: Flanker)				x	x	x	x	x	x	x	x	x	x			
EEG pre- and post-stimulation				x	x	x	x	x	x	x	x	x	x			
Safety Questionnaire				x	x	x	x	x	x	x	x	x	x			
AE documentation		x	x	x	x	x	x	x	x	x	x	x	x	x	x	x

* only applies when participants are eligible for MR assessment ** In case, intervention sessions are missed due to illness, participants can attend sessions at a later time point if the time between V1 and V10 does not exceed three weeks.

V. List of Abbreviations

**ADHD**	Attention deficit hyperactivity disorder
**AE**	Adverse Event
**ASD**	Autismus Spectrum Disorder
**CA**	Consortium agreement
**CAU**	Christian-Albrechts-University Kiel
**CFT- 20 R**	Culture Fair Test – 20 Revised
**CI**	Coordinating Investigator
**CPT**	Continuous performance task
**CRF**	Case Report Form
**CRO**	Contract Research Organisation
**CSCC**	Center for Sepsis Control and Care
**DC**	Direct current
**dlPFC**	Dorsolateral prefrontal Cortex
**DMP**	Data Management Plan
**DRKS**	German Clinical Trials Register
**DSM-V**	Diagnostic and Statistical Manual of Mental Disorders V
**DSMB**	Data Safety Monitoring Board
**DTI**	Diffusion tensor imaging
**EC**	European Community
**eCRF**	Electronic Case Report Form
**ECG**	Electrocardiogram
**EEG**	Electroencephalogram
**EHI**	Edinburgh Handedness Inventory
**EMG**	Electromyogram
**EOG**	Electrooculogram
**EUDAMED**	European Databank of Medical Devices
**FDA**	Food and drug administration
**fMRI**	Functional magnetic resonance imaging
**FPI**	First patient in
**GCP**	Good Clinical Practice
**GU**	Goethe University
**Hz**	Hertz
**IC**	Informed Consent

**ICMJE**	International Committee of Medical Journal Editors
**IEC**	International Electrotechnical Commission
**IEC**	Independent Ethics Committee
**IMD**	Investigational medical device
**IQ**	Intelligence quotient
**ISF**	Investigator Site File
**ITT**	Intention-to-treat
**IMD**	Investigational Medical Device
**KKS**	Koordinierungszentrum für klinische Studien (Coordinating Center for Clinical Trials), OvGU Magdeburg
**K-SADS-PL**	Revised Schedule for Affective Disorders and Schizophrenia for School-Age Children: Present and Lifetime Version
**LPI**	Last patient in
**LPO**	Last patient out
**LTD**	Limited Company
**mA**	Milliampere
**MAC**	Media-Access-Control
**µV**	Microvolt
**MPG**	Geman Medical Device Law
**MPH**	Methylphenidate
**MPKPV**	German Regulation for Clinical Trials with Medical Devices
**MPSV**	German Safety Plan Regulation for Medical Devices
**MRI**	Magnetic resonance imaging
**NEC**	Neuroelectrics Cap
**NIC**	Neuroelectrics Instrument Controller
**OVGU**	Otto-von-Guericke-University of Magdeburg
**PDS**	Pubertal Development Scale
**PP**	Per-protocol
**rIFG**	Right inferior frontal gyrus
**RMS**	Root Mean Square
**SADE**	Serious Adverse Device Effect
**SAE**	Serious Adverse Event
**SAM**	Self-assessment manikin
**SAP**	Statistical Analysis Plan
**SCQ**	Social Communication Questionnaire
**SDV**	Source Data Verification

**SOP**	Standard Operating Procedure
**SPDX**	Software Package Data Exchange
**SPS**	Sampling per Seconds
**STIPED**	Stimulation in Pediatrics
**SUSAR**	Suspected Unexpected Serious Adverse Reaction
**TACS**	Transcranial alternating current stimulation
**TPB**	Theory of planned behavior
**TCS**	Transcranial stimulation
**TDCS**	Transcranial direct current stimulation
**TES**	Transcrabial electric stimulation
**TMF**	Trial Master File (of the Sponsor)
**TRNS**	Transcranial random noise stimulation
**UC**	Centro Hospitalar e Universitário de Coimbra
**UKSH**	Universitätsklinikum Schleswig-Holstein
**VAS**	Visual analogue scale
**WISC-III**	Wechsler Intelligence Scale for Children - III
**WHO**	World Health Organisation
**WP**	Work package
**ZIP**	Zentrum für Integrative Psychiatrie GmbH
**ZKS**	Zentrum für Klinische Studien (Center for Clinical Trials)

1 INTRODUCTION

1.1 Background

Attention deficit hyperactivity disorder (ADHD) is one of the most common childhood-onset psychiatric disorders with a prevalence of 5% (Polanczyk et al., 2007). It is characterized by developmentally inappropriate levels of inattention, impulsivity and hyperactivity (DSM-V) and strongly reduces health-related quality of life (Ravens-Sieberer et al., 2008; Kamp-Becker et al., 2011). Despite normal or even above average intellectual abilities and reasoning skills, children and adolescents with ADHD consistently show lower scores on academic achievement scales than same-aged peers, are more likely to repeat a grade or leave school without graduation (Barry et al. 2002). Symptoms often persist into adulthood (Mannuzza et al., 2003) and the severity of ADHD symptoms during childhood is related to adult impairment (Fredriksen et al., 2014).

For the treatment of ADHD, different pharmacological approaches, particularly the use of stimulants, have been shown to be effective. However, many children and adolescents do not respond sufficiently to these treatments or they experience side effects which can lead to discontinuation of pharmacotherapy (Bokor and Anderson, 2014; Clavenna and Bonati, 2014). Behavioural therapy demonstrates only small to medium effect sizes, is cost intensive, and may only be provided for a small proportion of patients (Evans et al., 2014; Gajria et al., 2014). Therefore, patients and their parents often wish for alternative treatment strategies.

In the proposed study, we will investigate whether the innovative and cost-effective treatment approach of transcranial direct current stimulation (tDCS) can be successfully employed in children and adolescents with ADHD. TDCS uses low-intensity electrical stimulation (0.5 – 2.0 mA) between anode and cathode electrodes mounted at the surface of the scalp (Nitsche et al., 2008). A weak direct current (DC) penetrates the skull, enters the brain and alters spontaneous neural activity under the stimulating electrode. In general, the positively charged anode increases cortical excitability while the negatively charged cathode decreases it (Nitsche and Paulus, 2000). This modulation is brought upon by a modification of the resting membrane potential in regions of current flow (Stagg and Nitsche, 2011). These effects can last up to several hours depending on the duration of the stimulation (Nitsche and Paulus, 2000) and are reversible. Active stimulation is usually contrasted with a sham-condition where an initial current is applied but downregulated after 30 seconds. Transcranial stimulation (TCS) has been successfully employed to modulate resting state activity (Zaehle et al., 2010) as well as various perceptual, motor and cognitive processes, particularly working memory (Boggio et al., 2006; Zaehle et al., 2011; Heimrath et al., 2012). Moreover, tDCS has been shown to improve resting-state functional connectivity of different intrinsic brain networks (Polania et al., 2010; Keeser et al., 2011). Research focusing on the therapeutic value of tDCS in adult neuropsychiatric disorders, such as depression, schizophrenia, substance abuse, stroke and pain, has been expanding tremendously (Brunoni et al., 2013; Kuo et al., 2014; Tortella et al., 2015; Elsner et al., 2016; Kekic et al., 2016), strengthening the hope of establishing a break-through method for mental health disorders (Bourzac, 2016).

Even though the majority of studies has focused on tDCS application in adult populations, there is now solid evidence that tDCS can be also safely used in children and adolescents (age 2 to 18) (Krishnan et al., 2015). Studies conducted in minors did not report considerable side effects, except itching and tingling skin sensations and transient redness of the skin under the electrodes (Mattai et al., 2011; Andrade et al., 2014; Moliadze et al., 2014; Moliadze et al., 2015). Compared with other treatments, tDCS has a favourable safety-feasibility profile, offers a convincing placebo and is inexpensive (Nitsche and Paulus, 2011). Furthermore, when stimulation is applied repeatedly, stable changes in brain activity can be established: Cohen Kadosh et al. (2010) trained children for six days while applying tDCS increasing performance in a numerical competence task compared to a sham-control group. This improvement was still present six month after training. Thus, particularly repeated exposure to a cognitive task in combination with tDCS can induce neural plasticity and long term behavioral effects providing the opportunity to positively influence atypical brain development early and persistently (Krause and Cohen Kadosh, 2013).

Although a recent systematic review indicated that ADHD is one of the most promising childhood-onset psychiatric disorders that may profit from tDCS (Muszkat et al., 2016), there have been only few studies investigating tDCS effects in ADHD patients. Using oscillatory tDCS over the dorsolateral prefrontal cortex during slow wave sleep, an improvement of declarative memory performance on the next day was demonstrated in children with ADHD (Prehn-Kristensen et al., 2014) as well as improved reaction times in a go/nogo task (Munz et al., 2015). Since a large body of evidence has shown that ADHD is associated with structural and functional alterations in the dorsolateral prefrontal

cortex (dlPFC), most studies, some in adults, have stimulated this region yielding positive effects of (repeated) stimulation on clinical measures (Soff et al., 2017), and functional connectivity (Cosmo et al., 2015; Sotnikova et al., 2017). In healthy adults, tDCS over the dlPFC has improved reaction times and in part accuracy during working memory tasks (Brunoni and Vanderhasselt, 2014). Working memory refers to a limited-capacity process responsible for maintaining and manipulating cognitive representations in mind for later use. Working memory dysfunctions have been shown to be a primary neuropsychological deficit in ADHD.

In ADHD, the ability to control sensory processes and actions in a goal directed manner (Bunge et al., 2002) is severely compromised. ADHD patients are particularly impaired in different aspects of inhibition control, namely interference control, the suppression of task irrelevant, competing stimuli and response inhibition, the suppression of a prepotent response. Interference control has been repeatedly associated with the right inferior frontal gyrus (rIFG) (Luks et al., 2010; Zhu et al., 2010), a region that shows structural (Sowell et al., 2003; Durston et al., 2004) as well as functional alterations (Aron and Poldrack, 2005) in ADHD patients. For example, unmedicated ADHD patients showed less activity in the rIFG during an inhibitory task than healthy controls (Rubia et al., 2011), but reached age adequate levels when medicated (Lee et al., 2010). Since tDCS over of the rIFG and the pre-supplementary motor area had been successfully used to improve response inhibition in healthy adults (Hsu et al., 2011; Jacobson et al., 2011; Ditye et al., 2012), Breitling et al. (2016) chose to apply tDCS over the rIFG in adolescents with (and without) ADHD during a Flanker task, targeting interference control. They showed that anodal tDCS over the right IFG improved interference control and reduced reaction time variability compared to ADHD patients receiving sham stimulation (Breitling et al., 2016). In this study, a post-hoc simulation of current density concentrations within the brain revealed very little focal but a widespread current density distribution. This is a problem in most conventional tDCS set-ups, where large pad electrodes are used (bipolar montage), making it difficult to judge whether stimulation effects result from anodal stimulation of the target region or cathodal stimulation of the so-called reference or return electrode. Current recommendations (Woods et al., 2016) therefore advocate the use of optimized multi-channel montages.

In the current protocol, we aim to investigate in two separate studies how optimized multi-channel stimulation of the left dlPFC **(Study A)** and stimulation of the rIFG **(Study B)** improves relevant cognitive functions and clinical measures in children and adolescents with ADHD. These studies will be conducted within the framework of the STIPED (Stimulation in Pediatrics) consortium.

1.2 Risk-benefit ratio

1.2.1 Risk-benefit ratio concerning the medical device

In the design and performance stage of Starstim's NEC box, general requirements for basic safety and essential performance were created according to Independent Ethics Committee (IEC) 60601-1:2005, IEC 60601-1-2:2007 and IEC 60601-1-11:2010. Afterwards, the device was verified and validated both in-house and in a third-party laboratory to confirm its compliance to the aforementioned standards. Thereafter, the risk analysis in compliance with ISO 14971:2007 and literature review were performed to assure the device is safe. The most common adverse effects reported in the literature are sensations under the electrodes (tingling, burning, itching), fatigue and redness or skin irritation under the electrode (Brunoni et al., 2011).

Based on most recent clinical evidence (Bikson et al., 2016), the use of conventional tDCS protocols in human trials (≤ 40 min, ≤ 4 milliamperes, ≤ 7.2 Coulombs) has not produced any reports of serious adverse effect or irreversible injury with over 33.200 sessions and 1.000 subjects with repeated sessions. This includes a wide variety of subjects, including persons from potentially vulnerable populations. Adverse effects of active vs. sham tDCS include itching (39.3% vs. 32.9%, $p>0.05$), tingling (22.2% vs. 18.3%, $p>0.05$), headache (14.8% vs. 16.2%, $p>0.05$), burning sensation (8.7% vs. 10%, $p>0.05$) and discomfort (10.4 % vs. 13.4 %, $p>0.05$). Post-market surveillance activities performed by Neuroelectrics in the European clinical market including the Clinical Evaluation Report confirm that no SAEs have been reported to date. All adverse events (AE) reported are rare, minor and transient.

The device Starstim32 used in the current proposal allows the use of an optimized multi-channel montage which provides a more targeted stimulation of the critical brain area.

1.2.2 Risk-benefit ratio concerning the clinical study

This clinical study is designed to investigate effect sizes of changes in neuropsychological performance and study safety and tolerability of tDCS in children and adolescents with ADHD. Although ADHD can persist into adulthood, inclusion of minors in this study is required for the following reasons: ADHD is recognized as a neurodevelopmental disorder (American-Psychiatric-Association, 2013) since it begins in childhood and neuroanatomical alterations become more progressingly apparent during development. Imaging studies revealed a dynamic delay in cortical maturation which begins to show in children of preschool age (Shaw et al., 2007).

A benefit is the access to controlled and monitored tDCS treatment, which is not offered as a standard treatment, yet. Access to this treatment is beneficial in particular for those patients who do not respond to standard treatment such as Methylphenidate (MPH). Potential benefits for participants receiving real stimulation can be a decrease in ADHD symptom load as suggested by Soff et al. (2017) who showed a reduction in inattentive symptoms after 5 days of stimulation. However, data from tDCS studies in adults reveal a strong interindividual variance (Lopez-Alonso et al., 2014) suggesting that treatment might be successful in some but not all ADHD patients receiving verum. Interestingly, the intraindividual variance is low. Thus, identifying and characterizing children and adolescents who profit from tDCS could lead to the individual recommendation for study participants to further pursue tDCS treatment.

Since the aim of the current proposal is to investigate tDCS as a potential alternative to pharmacological treatment, only patients who are currently not under MPH treatment or are willing to refrain from this treatment can be included in the study. This will most likely apply to families who have decided against pharmacological treatment or use MPH flexibly (e.g. only during school days). However, some patients are severely impaired in their daily functioning when they are off medication and are therefore at a higher risk to experience more than usual problems in school, with their family and friends when they receive sham stimulation or do not profit from treatment. This risk will be discussed clearly with families. Due to the rapid pharmacokinetic of MPH, resuming MPH treatment after study particpation is possible and the benefit of MPH will be present immediately.

A benefit for all patients, regardless whether they are allocated to the tDCS or the sham condition, consists in the detailed diagnostic process. The diagnostic procedure comprises a thorough assessment of cognitive skills and the presence of psychiatric disorders. Participants and their families can receive state-of-the-art information on ADHD and related problems. In addition, by using structural and functional brain imaging techniques (fMRI, DTI, EEG) the current state of the patient's brain is assessed and documented. Patients and their families are provided access to these advanced diagnostic information if they wish. Within the study, a clear policy on incidental findings is followed: In case unexpected or so far untreated abnormalities are found, patients can be referred to further specialists. Families will be informed of this possibility as part of gaining informed consent.

It needs to be pointed out that the number of diagnostic measures and questionnaires does not exceed the amount usually employed in research studies on ADHD. We particularly selected instruments that allow a brief but thorough assessment of the relevant variables. The same holds true for the neurobiological measures, namely EEG and MRI, which will be applied in the current trial. If exclusion criteria are applied and safety recommendations are considered, neither EEG nor MRI pose a risk for the study participants.

Studies examining explicitly the safety aspects of tDCS in adult populations have found no adverse effects related to motor performance, the spectral characteristics of electroencephalogram (EEG), or other clinical measures of brain function (Iyer et al., 2005; Gandiga et al., 2006; Tadini et al., 2011). The levels of neuron-specific enolase (NSE), a sensitive marker of neuronal damage, were not increased after tDCS (Nitsche et al., 2003), and no pathological changes were observed in contrast-enhanced MRI or EEG (Nitsche et al., 2003; Nitsche et al., 2004). A comprehensive review on safety aspects of the tDCS was published by an International Safety Consortium (Bikson et al., 2016), summarizing the most experimental (animal and human), clinical and computational studies which have been performed until 2016 in healthy subjects, patients and in theoretically vulnerable populations including children and the elderly, subjects with mood disorders, epilepsy, stroke, implants, and home users. Evidence from relevant animal and computational models indicates that brain injury by tDCS occurs at predicted brain current densities (6.3 – 13 A/m2) that are over an order of magnitude above those produced by conventional tDCS, such as used in the present clinical study (max. 1mA). Regarding long-term safety aspects in children and adolescents, the longest follow-up period was 1.5 years and no adverse responses were reported (Alon et al., 1998; Krishnan et al., 2015). Also regarding epilepsy the majority of studies in both adults and children showed that tDCS

does not elicit epileptic seizures or provoke epileptic EEG activity in EEG either in healthy subjects (Moliadze et al., 2015) or in children with known epilepsy (Varga et al., 2011; Bikson et al., 2016). Only in one single case an epileptic seizure was observed during a tDCS session conducted to treat epilepsy in a child. However, since the child suffered already from epilepsy it is doubtful whether tDCS indeed elicited the seizure. It seems more plausible to assume that this seizure occurred independently of the tDCS treatment and is probably not related to tDCS.

In our consortium, we investigated AE of tDCS systematically in different age and subject groups (Siniatchkin et al., 2012; Prehn-Kristensen et al., 2014; Moliadze et al., 2015; Munz et al., 2015; Breitling et al., 2016) participants tolerated the stimulation well with a low rate of AE during and after both stimulation and application of sham. No serious AE were reported. No participant dropped out of the studies because of AE. No serious adverse effects were seen in young patients, even after tDCS applied with a higher than usual current density (0.497 mA/cm2) and/or repeated over several days (Mattai et al., 2011; Schneider and Hopp, 2011; Göder et al., 2013; Munz et al., 2015), tDCS was applied to some children during sleep without awakening them, and none reported AE the following morning (Göder et al., 2013; Munz et al., 2015). Thus, we conclude that potential benefits outweigh the risks posed by participating in the clinical trial.

Great care will be applied to properly inform patients and their families about benefits and risk of the study and to clarify exclusion criteria. In case of serious AE, considered to be possibly or probably related to the intervention, the Data Safety Monitoring Board (DSMB, see also 8.9) will be asked to recommend on whether to stop or pursue the study.

2 OBJECTIVES

The current study aims to investigate in a sufficiently powered sample whether repeated multi-channel stimulation of the left dlPFC **(Study A)** or the rIFG **(Study B)** significantly improves relevant neuropsychological parameters in ADHD, namely interference control, response inhibition, and working memory short- and long-term (4 weeks). Results of the study will inform about potential effect sizes and the therapeutic potential of tDCS in ADHD.

2.1 *Study objectives*

2.1.1 Primary objectives

- Investigate effect sizes of changes in neuropsychological performance and study safety and tolerability of multi-channel anodal tDCS targeting the left dorsolateral prefrontal cortex (dlPFC) **(Study A)** or the right inferior frontal gyrus (rIFG) **(Study B)** in children and adolescents with ADHD

2.1.2 Secondary objectives

- Investigate the potential benefit of multi-channel anodal tDCS targeting left dlPFC **(Study A)** or the rIFG **(Study B) on** behavior and quality of life in children and adolescents with ADHD
- Identify individual factors derived from MRI and/or EEG measures as well as clinical measures to predict responsivity to stimulation
- Assess expectations and concerns of parents and participants towards tDCS

2.2 *Endpoints*

2.2.1 Primary endpoints

- **Study A:** Overall accuracy in an *n-back* task (assessing working memory): changes from V0 to V11
- **Study B:** Overall accuracy in a *Flanker* task (assessing inhibitory/interference control): changes from V0 to V11
- **Study A/B:** Ratings and observations of safety and tolerability aspects at all visits (1-10)

2.2.2 Secondary endpoints

- Severity of ADHD symptoms (inattention, hyperactivity/impulsivity): changes from screening/ V0 to V11 and V12
- **Study A:** Reaction time and reaction time variability in n-back task, number of omission and commission errors: changes from V0 to V11 and V12

  Study B: Reaction time and reaction time variability in Flanker task, number of omission and commission errors in congruent and incongruent trials: changes from V0 to V11 and V12
- Performance measures in neuropsychological tasks that assess impaired functions in ADHD but are different from the task presented during stimulation accuracy and reaction times / reaction time variability in

  Study A: (1) Flanker task and (2) and a Continuous Performance task (CPT): changes from V0 to V11 and V12

  Study B: (1) n-back working memory task and (2) and a Continuous Performance task (CPT): changes from V0 to V11 and V12
- Functional brain related outcome measures (1) *resting state EEG*: connectivity, phase coherence, Granger causality (2) *event related potentials*: amplitude, latency, latency variability, neuronal sources of task relevant components: changes from V0 to V11 and V12 (3) *resting state fMRI:* changes from V0 to V11
- Quality of life: changes from Screening/V0 to V12

2.3 *Substudies*

(1) Assessment of expectations and concerns towards the application of tDCS in participating children/ adolescents and parents at baseline (V0) and follow up (V12) at the following sites: Magdeburg, Kiel (ZIP, Univesitätsklinikum Schleswig Holstein (UKSH)), and Frankfurt (Michael Siniatchkin, Alena Buyx, UKSH, Kiel, Germany)

(2) Comparison of resting state fMRI, collected prior (V0_MRI) and post to the intervention (V11_MRI). Post assessments will only be conducted at the following sites: Magdeburg, Kiel (ZIP, UKSH), and Coimbra (Kerstin Krauel, OvGU, Magdeburg, Germany)

(3) Comparison of EEG components associated with working memory performance collected at baseline (V0) prior to the intervention (Alexander Prehn-Kristensen, Hannah Brauer, ZIP, Kiel, Germany)

(4) MRI data collected at baseline (V0) are evaluated to develop biomarkers to accurately predict response to tDCS for individual cases in order to inform clinical decision making (Christine Ecker, GU, Germany, work package (WP) leader of WP6 in STIPED)

(5) EEG data of V0, V11, and V12 as well as fMRI data of V0_MRI and V11_MRI are compared to identify ongoing and stimulus related EEG and fMRI based brain connectivity signatures to understand the role of tDCS interventions (Miguel Castelo Branco, UC, Portugal, leader of WP7 in STIPED, and principal investigator of E-StimADHD at Coimbra)

(6) To judge whether the baseline data of the participants collected in E-STimADHD reflect typical or atypical development, behavioral as well as EEG- and MRI data will be compared with a healthy control group collected in WP3 (lead: Vera Moliadze, UKSH, Kiel, Germany)

3 STUDY DESIGN

Both **Study A** and **Study B** of **E-StimADHD** are randomized, two arm, double-blind, parallel group, sham-controlled studies with three measurement points (screening/baseline (V0); post-intervention assessment (V11); four weeks follow-up (V12)). The intervention is a repeated anodal direct transcranial current stimulation (tDCS) over the left dlPFC **(Study A)** or the rIFG **(Study B)** during an ongoing task (10 days of stimulation 20 min / day, with 1mA intensity). Blinded sham stimulation is applied in the same setting without an effective electrical current. The two arms represent 1) anodal tDCS+task; 2) sham stimulation+task. The studies will be conducted in the laboratory setting.

4 SCHEDULES (TIMELINES)

Planned recruitment period:	1 - 25 (Months)
Planned treatment period:	1 - 26 (Months)
Planned follow-up period:	2 - 27 (Months)
Planned first patient in (FPI):	Q3/2017 (Quarter/Year)
Planned last patient in (LPI):	Q3/2019 (Quarter/Year)
Planned last patient out (LPO):	Q3/2019 (Quarter/Year)
Duration of study intervention per subject:	2 x five consecutive days with a two days break in between). In case, intervention sessions are missed due to illness, participants can attend sessions at a later time point if the time between V1 and V10 does not exceed three weeks.
Duration of follow-up per subject:	4 weeks

5 SITES AND SUBJECTS

5.1 *Selection of sites*

We selected five sites that have extensive experience and expertise in the investigation and treatment of children and adolescents with ADHD. Participating sites are (1) the ICNAS, Clinical and Academic Center, University of Coimbra (UC), Portugal, (2) the Department of Child and Adolescent Psychiatry, Psychosomatics and Psychotherapy at the University Hospital Frankfurt, Goethe University (GU) Frankfurt am Main, Germany, (3) the Institute of Medical Psychology and Medical Sociology, University Clinics of Schleswig-Holstein (UKSH), Christian-Albrechts-University (CAU) Kiel, Germany (4) the Department of Child and Adolescent Psychiatry and Psychotherapy, Zentrum für Integrative Psychiatrie (ZIP), Kiel, Germany, and (5) the Department of Child and Adolescent Psychiatry and Psychotherapy, Medical Faculty, Otto-von-Guericke-University (OvGU) Magdeburg, Germany. Moreover, all sites are highly experienced in the use of various neuroscientific methods and have state-of-the-art EEG and MRI facilities at their disposal.

5.2 *Selection of participants*

It is planned to include 100 participants between the age of 10 to 18 with ADHD per study, totaling 200 patients. We will consider patients of the inattentive *(≥ 6 inattentive symptoms, but less than 6 hyperactive/impulsive symptoms)*, hyperactive/impulsive *(< 6 inattentive symptoms, but ≥ 6 hyperactive/impulsive symptoms)* and combined subtype *(≥ 6 inattentive symptoms, and ≥ 6 hyperactive/impulsive symptoms)*.

Justification for gender distribution

In ADHD populations, there is a sex distribution in favour of male patients as boys are approximately four times as likely to be affected as girls (Kern et al., 2015). Consequently, the gender distribution in the recruited sample will reflect this population-based imbalance (approximately 4:1).

5.2.1 Inclusion criteria

- ADHD according to DSM-5 (all subtypes)
- age 10 to 18
- the participant and their custodians are able and willing to give written informed assent/consent

5.2.2 Exclusion criteria

- IQ < 80
- Birth weight < 2500 gr.
- Born before the 37th week of pregancy
- Past or present neurological diseases/brain surgery
- Dermatological diseases of the scalp
- All comorbid psychiatric disorders other than oppositional defiant disorder, conduct disorder, elimination disorders, anxiety disorders, learning disorders
- History of craniocerebral injury with loss of consciousness
- Heart disease
- Pregnancy
- Concurrent neurofeedback therapy
- Concurrent pharmacological treatment of ADHD
- Participation in another clinical study

5.2.3 Concomitant medication and therapy

The main aim of the proposed study is to assess the potential effect sizes tDCS can have on impaired neuropsychological performance in ADHD as a possible alternative to stimulant medication. Therefore, it is essential to obtain pre- and postintervention measures devoid of the normalizing stimulant effect. From clinical experience, families often use stimulants flexibly e.g. refrain from medication during the weekends or vacations. Moreover, a considerable number of patients do not use medication to treat their ADHD at all. Participants will be asked to refrain from stimulant use throughout the entire study, at least two weeks prior to V0. Time without stimulant treatment can vary between 7 to 15 weeks

between participants depending mostly on the time windows between screening and V0 and the beginning of the intervention. Beside stimulant treatment, concomitant neurofeedback therapy is not allowed. Other ongoing interventions such as behavior therapy or occupational therapy can be continued.

5.3 Recruitment measures

Local in- and outpatient departments of the participating clinical centers will recruit a sample of 200 ADHD patients (**Study A** = 100 / **Study B** = 100). We will perform an open recruitment strategy expecting to reach the following recruitment rates: Coimbra: N=30/30, Frankfurt: N=20/20, Kiel UKSH: N=12/13, Kiel ZIP: N=13/12, Magdeburg: N=25/25 individuals with ADHD). All sites have access to large clinical databases and in- and outpatient departments, so families and adolescents with ADHD can be approached and specifically invited to participate in the study. Patient families registered at the in-house data bases will be contacted by telephone; further patients will be recruited either via flyers placed at the in- and outpatient departments or by announcements published in the local newspapers and the internet. Local pediatricians and child and adolescent psychiatrists and psychotherapists will be informed about the study.

6 MEDICAL DEVICE

6.1 Device description

The Neuroelectrics product, Starstim 32 is a battery powered, wireless, 32-channel transcranial current stimulation (tCS) and an EEG monitoring class IIa device (Figure 1). It uses different types of current stimulation (tACS, tCS, tRNS) and is capable of acquiring a variety of electrophysiological signals. These signals include EEG, Electrooculogram (EOG), Electrocardiogram (ECG) and Electromyogram (EMG).

The Starstim systems components are primarily made up of the following three items:

- NECbox (amplifier, transmitter and control box)
- the neoprene cap with the electrodes, cables
- the NIC software

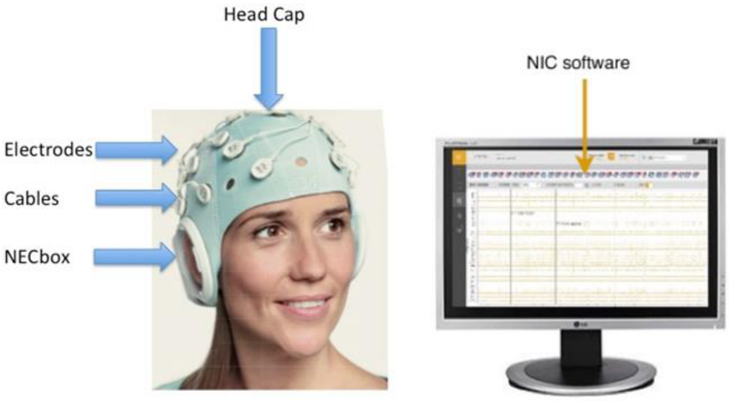

Figure 1. Components of the Starstim system

The used hybrid tCS & EEG electrode is called Pistim. It is a unique electrode due to its dual functionality: it works for stimulation and for EEG monitoring. Thus, it is the ideal electrode when EEG monitoring is required in a stimulation session. Further, due to its reduced size, it is perfect for focal multi-electrode stimulation experiments. The Pistim provides a 3.14 cm^2 circular contact area and a rear-fill aperture for gel supply. It is based on sintered Ag/AgCl pellet of 12 mm diameter.

Device characteristics

- EEG recording is possible before, during and after stimulation
- Multiple independent stimulation channels improve the spatial distribution of the electric field
- Variety of waveforms for stimulation current: tDCS, tACS, tRNS and sham mode

- Custom waveforms can be used for stimulation protocols
- It contains hybrid electrodes that can be used for EEG and tCS
- Ease of use despite of the complexity of the technology
- Safety features such as maximal currents and impedance control

EEG functionality

- Number of channels: up to 32 channels
- Sampling rate: 500 SPS
- Bandwidth: 0 to 125 Hz
- Resolution: 24 bits - 0.05 μV
- Measurement noise: < 1 μV RMS
- Input impedance: 1000 MΩ minimum

Stimulation functionality

- Allows for stimulation and EEG recording simultaneously
- Number of channels: up to 32 channels
- Types of Stimulation: tDCS, tACS, tRNS, sham
- Current: maximum of ± 2mA per electrode
- Current resolution: 1 μA
- Current accuracy: 1%
- Battery-powered
- Maximum voltage: ± 15V per electrode (allows 30V of stimulation potential difference)

6.2 Components of the device

In terms of manufacturing, Starstim NECbox is composed of three main parts: an electronic board, a case, and a battery. The electronic board is manufactured by Lab Circuits and the assembly of the components is subcontracted to one of the main providers of Neuroelectrics, DigiProces. The case is subcontracted to a specialized company in industrial designs, called Ecceleni. Additionally, batteries are purchased at local retailers as they are standard rechargeable 3.7 V lithium ion batteries. Finally, the assembling of the mentioned parts is conducted in Neuroelectrics' factory.

Most of Starstim's electrodes are manufactured in Neuroelectrics' facilities. Pistim electrodes (Ag/AgCl) are subcontracted to Sautel and Sponstim electrodes are subcontracted to Canadian Medical Products LTD. Canadian Medical Products LTD has the Food and Drug Administration (FDA) establishment registration number: 802219. The neoprene headcap and headband manufacturing are subcontracted to a tailoring workshop in Spain, called Proline. Each electrode should be replaced after 10 hours of stimulation because the Ag/AgCl is expected to be consumed.

The electrode gel is a highly conductive and bacteriostatic gel. It is water soluble, non-staining and non-gritty. It is used for EEG monitoring electrodes and for the hybrid EEG/tCS Pistim electrodes. It is used to improve the contact between the electrode and the scalp by decreasing the impedance. The 12 ml curved syringe is used to inject either electrode gel or saline in the electrodes. With the Geltrode and the Pistim, the curved tip of the syringe should be inserted on the small hole of the electrode to inject the electrode gel into the rear-fill aperture.

NIC is the proprietary software. The software manages the basic control of the device and sends the commands to the device and receives the status and the data from the NECbox. NIC controls the Windows API which manages the Starstim-computer pairing process and data reception. Once paired, one can also intentionally revert to the unpaired state by closing the software or pairing to another device. Once the Bluetooth® connection is established, NIC can be used to direct Starstim to record EEG signals or apply neurostimulation protocols. The software does not perform the core functionalities of the device. The NECbox has an internal control that takes the control of the generation of the stimulation waveforms, the impedance control and the EEG monitoring management.The NIC software is fully created and developed in Neuroelectrics, in Spain. NIC Software is in compliance with IEC 62304: 2006. The software is classified as Class B following the IEC 62304:2006. Neuroelectrics periodically inspects their critical suppliers (according to the internal procedure GP-010 - Supplier's Evaluation) to control that their products and services conform to specified requirements.

For blinding the administrator and operator, rules will be assigned. The administrator will be the person who can create and manage the protocols. In a double blind study, the administrator will create a series of protocol templates for stimulation and give them generic names that do not provide montage related information. One protocol will refer to the real stimulation sessions, while a second protocol will correspond to the sham stimulation. The administrator will also prepare a list for the operator assigning a protocol to each subject and session (e.g. Subject01_Session01). The operator will be responsible for selecting the appropriate protocol for the session/subject, setting up the electrodes according to the montage, checking impedances, launching it and annoting the session as needed. During the stimulation session, the operator will have no access to protocol data (currents, sham settings, EEG traces, etc.) and will remain effectively blinded.

6.3 Manufacturer

Neuroelectrics Barcelona S.L.U.
Avenida Tibidabo, 47 bis
08035 Barcelona, Spain

Tel: +34 93 254 03 68
Fax: +34 93 212 64 45
Email: rafal.nowak@neuroelectrics.com

Neuroelectrics is an ISO 13485 and ISO 9001 certified company http://www.neuroelectrics.com/ . Thus, their medical devices are designed and manufactured following the corresponding ISO quality management systems. Neuroelectrics complies with Quality System Regulation 21 CFR 820. Neuroelectrics manufactures all their products in a factory located in Barcelona, Spain. The factory is yearly inspected by the SGS United Kingdom Limited, Notified Body number 0120, 202 Worle Parkway Weston-super- Mare, BS22 6WA UK. Last year, Neuroelectrics was successfully audited on May 23, 2016. Compliance is supported by the CE Certificate number ES12/11935 Production Quality Assurance.

6.4 Serial number or batch label

Each device is marked using the following code: EYYYYMMDD-XX (Figure 2). Where YYYTMMDD indicated the date in the year/month/day format when the device was produced and XX is an incremental counter for the devices in the same day. The devices have also the unique MAC address at the label.

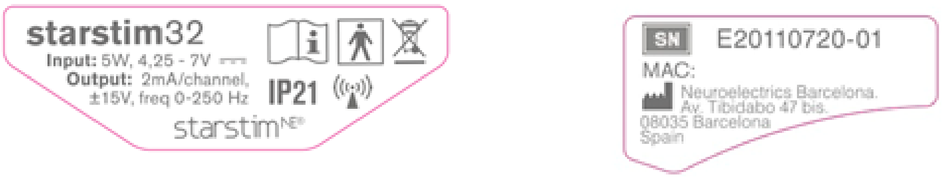

Figure 2. Starstim R32 labels

Each device will have attached a sticker providing the following information: study title (STIPED: E-StimADHD), EUDAMED number, sponsor´s name (KKS Magdeburg), a unique serial number, a contact address of the local physician who is responsible for the clinical trial in case of emergencies, and a warning that the device is “only intended for clinical investigation”.

6.5 Storage, shipping and return as well as documentation of medical devices (device accountability)

6.5.1 Storage

Starstim32 must be used with normal temperature, humidity and pressure conditions:
- Temperature Range: +5 to 40 °C
- Humidity: 15 - 93 %
- Atmospheric Pressure: 700 - 1.000 hPa

The device must be stored inside the box between uses, in the following environmental conditions:
- Temperature Range: -25 to +70 °C
- Humidity: 15 - 93 %

This equipment needs to be installed and put into service in accordance to the information provided in this user manual.

6.5.2 Packaging

The primary package contains the components of the Starstim in a foam box organized in a way to be also a functional box to storage the product during the rest of the life of the product. In the case of Starstim, primary and secondary packaging are the same. The packaging box is common for all the Neuroelectrics' products, and the Label identifies which product it contains. The size of the box is: 350 x 240 x 75 mm in a closed position. The primary and secondary packaging are used for the shipment and storage of other products. There has not been any damage or reported problem related to the storage or shipment. The primary and secondary package containing Starstim are compliant with IEC 60601-1:2005 and IEC 60601-1-11:2010.

For traceability purposes, the primary and secondary packaging contain a Label including the following information:

- Name and address of the manufacturer
- Product model and description
- Serial Number and Mac of the device

6.5.3 Documentation

An investigator shall maintain the following accurate, complete, and current records relating to the device use in an investigation:

1. Records of receipt, use or disposition of a device that relate to:
   - The type and quantity of the device, dates of receipt, and batch numbers or code marks
   - Names of all persons who received, used, or disposed of each device
   - The number of units of the device returned to the manufacturer, repaired, or otherwise, disposed of, and the reason(s) therefore.
2. Records of each subject's case history and exposure to the device, including:
   - All relevant observations, including records concerning adverse device effects (whether anticipated or not), information and data on the condition of each subject upon entering, and during the course of, the investigation, including information about relevant previous medical history and the results of all diagnostic tests
   - A record of the exposure of each subject to the investigational device, including the date and time of each use, and any other therapy.
3. The protocol, with documents showing the dates of and reasons for each deviation from the protocol
4. Any other records that requires to be maintained by regulation or by specific requirement for a category of investigatetions or a particular investigation.

6.5.4 Return

Upon completion of the trial, investigators are required to return to the manufacturer any remaining supply of the device or otherwise dispose of the device as the manufacturer directs. It has to be documented for each trial site.

6.6 Directions for use of medical devices

All sites will be trained and supervised by personnel certified in the use of Starstim32. Relevant information conveyed during training is based and documented in the User Manual (I, II & III).

7 STUDY PROCESS

7.1 Study enrollment

7.1.1 Assignment to study arm / randomisation

After definite inclusion into the study (signed informed consent), each patient will be allocated randomly to one of the two studies (**Study A** or **Study B**) and within each study to one of the two treatment conditions (anodal tDCS stimulation or sham stimulation) using a randomized block design. Each block will consist of four patients. Block randomization will be done with the electronic randomization tool BiAS for Windows (version 11, 2016; see chapter 11.2). The randomisation results will be transmitted to the center through the eCRF system in the form of the file name (file containing protocol templates for stimulation) which only reveals the assigned study (Study A or Study B, necessary to perform the specific tasks of the study), but not the treatment condition (sham or real stimulation).

7.1.2 Blinding

Subjects will be blinded to treatment allocation for the whole duration of the trial as the sham stimulation is indistinguishable from real stimulation. The device Starstim32 (Neuroelectrics, Barcelona, Spain) allows to perform double-blind stimulation. The administrator, the person who can create and manage protocols, will create a series of protocol templates for stimulation prior to randomization and give them names that do not provide stimulation related information. The statistican creates a material file and needs to know the exact data file names of each scenario. During the stimulation session, the operator will have no access to the protocol data (currents, shamsettings) and thus remain effectively blinded.

7.1.3 Unblinding

Unblinding will be done in case of the following emergencies:

- Decision of investigator: unblinding is necessary for reasons of subjects safety and for decision of further medical treatment
- A Serious Adverse Event (SAE) becomes a Suspected Unexpected Serious Adverse Reaction (SUSAR) and has to be unblinded for notification to authorities and ethics committtee.

Unblinding will be performed through emergency envelopes on site, which contain information of sham or stimulation. The participant and his/her parents have to be informed about unblinding. The Sponsor has to be informed in writing about any unblinding process and the process itself has to be documented in detail on site. Unblinding leads to the exclusion of the respective participant.

7.2 Visits

Screening visit

1. Information of participants and their parent(s) and confirmation of consent
2. Assessment of inclusion and exclusion criteria
    - Medical and psychosocial history
    - K-SADS-PL
    - IQ test
    - Social Communication Questionnaire (SCQ)
    - Assessment of MRI criteria
    - Pregnancy test (urine test)
3. Assessment of participants´ characteristics
    - Edinburgh Handedness Inventory (child)
    - Social economic status of the parent(s)
    - Pubertal Developmental Scale (child, parent)
    - Expectations and concerns towards tDCS (child/parent)
    - ADHD rating scale (parent)*
    - Quality of Life (KIDSCREEN-27, child/parent)*

The last two questionnaires can be filled out at home and be returned at baseline.*

Baseline visit (Visit 0, within 4 weeks after screening)

- Assessment of arousal, mood and motivation before and after the session (SAM, VAS, diary)
- Resting state, 64-channel EEG
- Neuropsychological performance (tasks: n-back, Flanker, CPT), 64-channel EEG
- Assessment of adverse events
- Randomization

Baseline visit (Visit 0_MRI, within 4 weeks after screening), if participants are eligible for MRI

- Assessment of arousal, mood and motivation before and after the session (SAM, VAS, diary)
- Structural MRI (T1-weighted, T2-weighted)
- Resting state fMRI
- Diffusion tensor imaging (DTI)

Visit 1 to 10 (V1 within 4 weeks after V0)

- Assessment of arousal, mood and motivation before and after the session (SAM, VAS, diary)
- Multichannel stimulation (sham/real) of either the dlPFC **(Study A)** or the rIFG **(Study B)** during a task
- EEG before and after the multichannel stimulation
- Assessment of adverse events during stimulation (safety questionnaire)
- Assessment of adverse events

Visit 11 (Post assessment within 7 days after V10)

- Assessment of arousal, mood and motivation before and after the session (SAM, VAS, diary)
- Resting state, 64-channel EEG
- Neuropsychological performance (tasks: n-back, Flanker, CPT), 64-channel EEG
- ADHD rating scale (parent)
- Assessment of adverse events

Visit 11_MR (MRI, within 7 days after V10), if participants are eligible for MRI

- Assessment of arousal, mood and motivation before and after the session (SAM, VAS, diary)
- Resting state fMRI
- Assessment of adverse events

Visit 12 (Follow-up, 4 to 5 weeks after Visit 10)

- Assessment of arousal, mood and motivation before and after the session (SAM, VAS, diary)
- Resting state, 64-channel EEG
- Neuropsychological performance (tasks: n-back, Flanker, CPT), 64-channel EEG
- ADHD rating scale (parent)
- Quality of Life (KIDSCREEN-27, child/parent)
- Expectations and concerns towards tDCS (child/parent)
- Assessment of adverse events

7.3 Study related procedures

(I) Pregnancy test

Female participants who already experienced their menarche are required to provide a urine sample during screening. Pregnancy will be determined via standard laboratory procedure at the site.

(II) Verbal assessments

Medical and psychosocial history

Covers developmental milestones, past and present somatic illnesses and treatment (surgery), past und current medication, ADHD treatment history

K-SADS-PL

To inquire about ADHD symptoms and relevant comorbidities, all sites will use the Revised Schedule for Affective Disorders and Schizophrenia for School-Age Children: Present and Lifetime Version (K-SADS-PL) (Kaufmann et al., 1997; Delmo et al., 2000) which generates diagnoses by integrating the child's and parents' reports. This semi-structured interview is especially suited due to its high inter-rater reliability as well as concurrent validity with clinical rating scales (Kaufmann et al., 1997; Kim et al., 2004). Moreover, it is very frequently used and well accepted in international research on ADHD.

(III) Written assessments / questionnaires (all are available in Portugese and German)

Socioeconomic status of the parent(s)

Socioeconomic status will be determined for the parent the child lives with most of the time. It is derived from the net income of the household, school graduation level and the current professional situation of the parent according to Lampert et al. (2014). This approach has been employed in a large German health survey in children and adolescents (Lampert et al., 2014). It allows a differentiation in low, middle, and high socioeconomic status.

Social Communication Questionnaire

We will use the well established Social Communication Questionnaire (SCQ) as an additional diagnostic tool (Rutter et al., 2003). It consists of four scales: social interaction, communication, abnormal language, and stereotyped behavior and has satisfactory internal consistency coefficients (84 to .93) across age groups The SCQ provides a cut-off score indicating the likelihood of autism spectrum disorder in an individual.The SCQ consists of 40 questions and can be applied in less than *10 minutes*.

Intelligence

To screen for general intelligence, the German sites will use the first part of the Culture Fair Intelligence Test – Scale 20 (CFT-20-R) (Weiss, 2008) which is a basic non-verbal measurement of general cognitive abilities particularly of cognitive flexibility and problem solving (*duration: 20 min.,* retest reliability: .72 to .91, average correlation with other intelligence assessments e.g. Wechsler Scales: .64). Coimbra will use the performance IQ of the WISC-III which also focusses on non-verbal intellectual skills (*duration: 30 min,* retest reliability: .74 to .77). In case IQ assessments using the CFT-20 R (Germany) or the WISC-III (Portugal) are obtainable that are not older than a year, IQ tests do not need to be redone.

Edinburgh Handedness Inventory (EHI)

To control for participants´ handedness we will use the Edinburgh Handedness Inventory (Oldfield, 1971). The EHI is a short, 10-item scale used to assess the dominance of a person's right or left hand in everyday activities and is widely used in research studies *(duration: 5 min.).*

Pubertal Development Scale (PDS)

Using only three items of the *Pubertal Development Scale (*PDS, *(Watzlawik, 2009)* allows to measure the pubertal stage without visual inspection by a clinician. The single sum score differentiates five different pubertal stages for boys and girls (*duration: 3 min.*; Cronbach´s alpha: 59 to .71 self-ratings, .64 to .81 parental ratings.).

ADHD rating scale

The ADHD rating scale comprises all 18 diagnostic criteria (9 inattentive / 9 hyperactive/impulsive) for ADHD according to the DSM-V. Parents and participants rate the frequency of each symptom on a four-step scale. The rating scale allows for separate calculation of overall symptom load, as well as for severity of inattentive and hyperactive/impulsive symptoms *(duration: 10 min).*

KIDSCREEN-27

The KIDSCREEN-27 allows to assess health-related quality of life in children and adolescents (age: 8 to 18) and has been widely used in international European health surveys (Ravens-Sieberer et al., 2005; Ravens-Sieberer and Europe, 2006). It measures quality of life in different domains, namely *Physical Well-Being*, *Psychological Well-Being*, *Autonomy & Parents*, *Peers & Social Support* and *School Environment* (*duration: 10-15 min*., reliability of each domain > .7).

Expectations and concerns of parents and participants towards tDCS

In order to investigate motivation/intention towards application of tDCS in pediatric population, a special questionnaire was developed based on the well-established theory of planned behavior (TPB) (Ajzen, 1991). The TPB aims to predict and understand why an individual may perform certain behaviors. Behavioral intention is a product of distal constructs such as attitude, normative beliefs, and perceived behavioral control. Research supports the usefulness of the TPB in the prediction of behavioral intention and performance for a wide variety of health behaviors and behavioral interventions (McEachan et al., 2011). The TPB enables not only an assessment of the strength of motivation/behavioral intention (for example towards application of tDCS) but also a differentiation which of the mentioned constructs is essential for explaining attitudinal changes and needs to be considered to improve adherence to a specific intervention (McDermott et al., 2016) *(duration: 5 minutes).* In addition, four open questions regarding expectations and concerns towards tDCS will be posed to children and parents in the course of the clinical interview.

Safety questionnaire

All sites will use the questionnaire of sensations related to transcranial electrical stimulation (TES) to assess discomfort during the stimulation (Poreisz et al., 2007). Participants are asked to rate a variety of sensations e.g. itching, burning on a four-step scale *(duration: 5 min).*

Self Assessment Manikin (SAM)

The Self-Assessment Manikin (SAM) scales are a well-established, non-verbal (pictorial) rating system to obtain self-assessments of experienced emotions on the dimensions affective valence, dominance and arousal in adults and children *(Bradley and Lang, 1994)*. All sites will use this method (three 9-point scales) to measure the emotional state/mood (*duration: 1 min*; Cronbach´s alpha: .63 to .98).

Visual analogue scale (VAS) for motivation and alertness

On two 10-cm VASs we will assess the current motivation (0 = not at all; 10 = highly motivated" and alertness (0 = very tired; 10 = very alert) in the beginning and in the end of all visits (*duration: 1 min*).

Diary

Participants are asked for acute consumption of caffeine, energy drinks or intake of medication, occurrence of significant events in school or at home in the beginning of visits 0-12 (*duration: 3 min*).

(IV) Neurobiological assessments/measures

MRI measures (structural T1 and T2, resting state fMRI, DTI) are collected at all sites using 3T scanners (Coimbra: Siemens TRIO; Frankfurt: Siemens TRIO, Kiel: Philips Achieva; Magdeburg: Siemens PRISMA), which have been employed at all sites for the investigation of children and adolescents. Acquisition settings were developed and defined to provide sufficient data amount and quality to develop biologically-driven models for the prediction of inter-individual variability in response to tDCS as well as to apply individual head modeling and electrical current estimation. The MRI session (V0_MRI) will last approximately *one hour*, V11_MRI about 30 minutes.

EEG during baseline, post-intervention and follow-up will be collected from 64-channels to identify electrophysiological changes due to the intervention. A larger array of electrodes is used to be able to investigate connectivity features *(duration: about 2 hours)*

SOPs are specified for all measures to ensure homogenity of data quality at all sites.

(V) Intervention: tDCS

- All sites will use an optimized multi-channel montage to target the dlPFCD **(Study A)** or the rIFG **(Study B)** (see Figure 3)

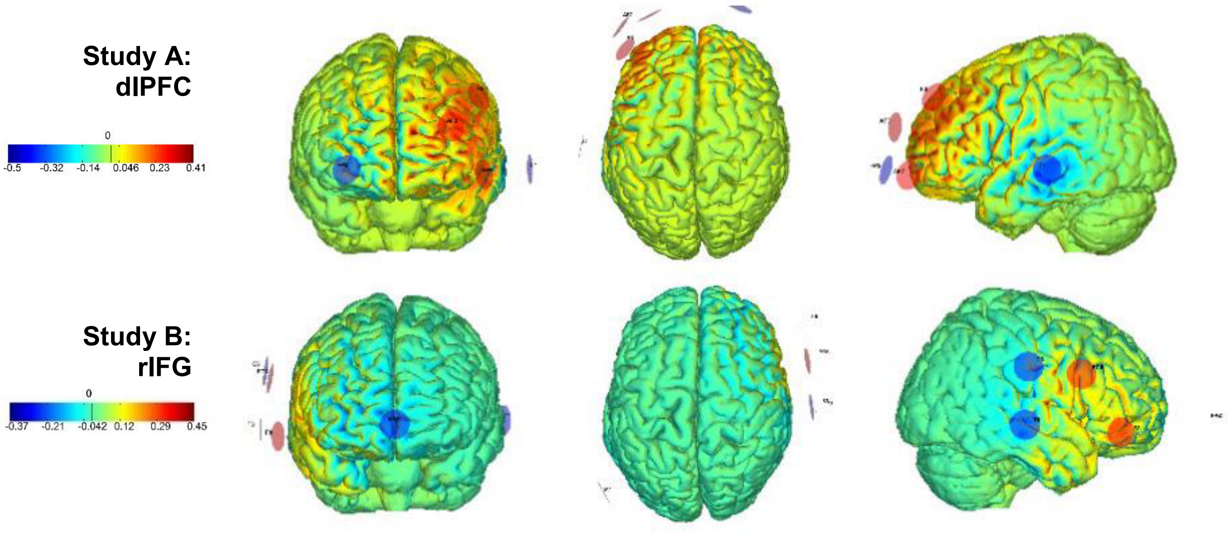

Figure 3: Optimized multi-channel montage for (upper row) dlPFC (Study A) and (lower row) rIFG (Study B). Red circles indicate locations of anodal electrodes, blue circles indicate locations of return electrodes.

- EEG will be recorded prior and post to real/sham stimulation in all patients to control for stimulation effects on brain activation. EEG during stimulation can require time-intensive readjustment of electrodes before starting stimulation. Concurrent EEG will therefore only be recorded in patients where no substantial readjustment is necessary to keep strain during the intervention at a minimum.
- Real stimulation: 1 mA, stimulation duration 20 min.
- Control intervention: sham stimulation (30 s ramp up – 30 s ramp down)
- Participants will take part in 10 sessions within a time interval of three weeks. We will aim to schedule 2 x 5 consecutive days with a 2-day break in-between. However, to minimize missed intervention dates of out-patients e.g. due to sickness or other significant events, we will allow continuation of treatment if the 10 sessions can be realized within three weeks.

(VI) Neuropsychological tasks

Flanker task
In the **Flanker task** (Figure 4, upper panel), stimuli consist of five arrows. The middle arrow is the target stimulus and participants should indicate via button press with the right or with the left hand if the target arrow points to the right or to the left. The outer arrows serve as distractors and can point in the same direction *(congruent trial)* or in the opposite direction *(incongruent trial)*. Each block consists of 184 trials and has a duration of 4.5 minutes, with 50% of the trials being congruent and incongruent. Stimuli are presented for 60 ms and participants have 1360 ms to respond. The interstimulus interval is 1460 ms. During the intervention (V1-V10) the task will be presented for four blocks. However, during baseline (V0), post-stimulation (V11) and follow up (V12) sessions, the task will be presented for two blocks (about 10 minutes) to minimize strain for the participants, since the CPT and the n-back task will be also presented during these visits to test for transfer effects.

N-back task
We will use the **n-back task** (Figure 4, right lower panel) with n=2. For this purpose, a series of objects will be presented with 25% of the trials being target trials meaning that the current stimulus is identical with the stimulus two trials earlier. Thus, the task requires continuous processes of encoding, retrieving and updating contents of working memory. The presented objects in each block consist of 10 different photographs that are presented 10 times each and serve as a target twice. Each trial has a duration of 2400 ms of which each object is displayed for 500 ms, preceded by a fixation cross for 1900 ms. The task will be conducted for 3 runs (5 minutes per run) with 1 min. pauses in between.

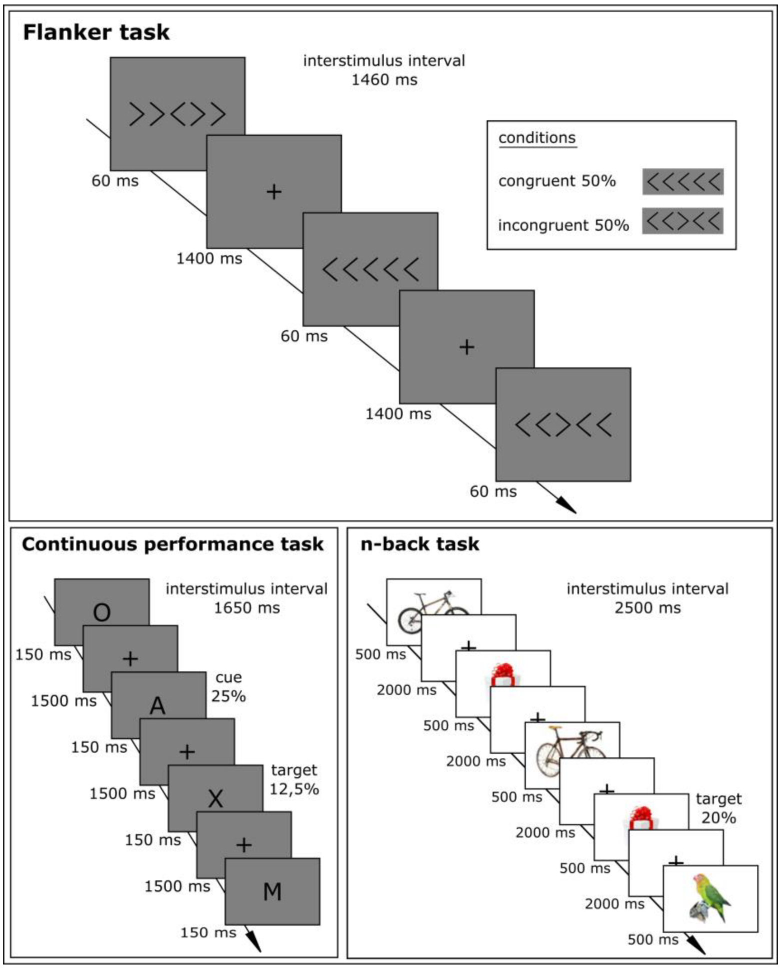

Figure 4. Examples of single trials of the Flanker task, the Continuous Performance task (V0, V11, V12) and the n-back working memory task

Continuous performance task
In the **Continuous performance task** (CPT) (Figure 4, left lower panel), various upper case letters will be presented. One of the letters will be defined as a cue and one as a target stimulus. Whenever the cue is followed by the target stimulus, participants are instructed to press a button. However, participants should withhold their response if the cue is followed by another stimulus. The task will consist of 3 runs á 4.5 minutes (80 stimulus sequences per run, 20 target sequences, 20 non-target sequences) with short breaks in between. For each trial the stimulus is presented for 150 ms and participants have 1400 ms to give their response (interstimulus interval = 1650 ms).

Task performance during each intervention will be assessed (accuracy, commission and omission errors, reaction time and reaction time variability, **Study A:** n-back task, **Study B**: Flanker task) to control for potential learning and motivational effects.

7.4 End of study and termination

7.4.1 Regular end of study

The study ends with the last follow-up visit of the last patient.

7.4.2 Premature end of study

7.4.2.1 Withdrawal of study subject

Decision of study subject
Children and adolescents as well as their caregivers can refrain at any time and without providing reasons from study participation. They will be informed that their concurrent or subsequent treatment

will not be affected or compromised because of the patient's decision. All participants will be offered a final physical examination once they participated in at least one intervention session and regardless after which visit they decided to stop participation.
All randomised patients will be asked to provide their consent to be assessed for the primary outcome, in particular, those who discontinue treatment prematurely to keep missing data due to loss to follow-up at a minimum.

Decision of investigator or Sponsor
Moreover, the investigator can decide to end study participation if there is reason to assume that the intervention is harmful for the child/adolescent or that study participation is mainly motivated by the parent and is not wished for by the child/adolescent. Reasons for exclusion from the study will be documented in the electronic Case Report Form (eCRF).

Compliance
Patients who are non-compliant to a degree that either their safety or the integrity of data is at risk, may be excluded from further treatment at the investigator's discretion. This includes intake of medication during the study, aiming to destroy stimulation equipment, and voluntary random responses to the tasks. If possible, assessment of primary outcome will be done.

7.4.2.2 Termination of study at a study site
Both the investigator and the Sponsor reserve the right to terminate the trial at any time. Should this become necessary the procedures will be agreed upon after consultation between the two parties. In terminating the trial, the Sponsor and the investigator will ensure that adequate consideration is given to the protection of the best interests of the patients. Regulatory authority and EC will be informed.

The clinical trial can be terminated prematurely at the trial site by the responsible investigator, if

- the continuation of the clinical trial is considered as ethically and/or medically not acceptable
- the resources to continue the clinical trial are not sufficient anymore

7.5 Continued treatment and medical care of subject after end of study

Patients who are in care at the investigating sites will be able to continue their previous treatment after the end of the study. Participants who were referred to our study from other sources will receive councelling or intervention, as needed.

8 EFFICACY AND SAFETY

8.1 Assessment of efficacy

In both studies, accuracy in a neuropsychological task is employed to assess treatment efficacy. In **Study A** an n-back task is used to investigate working memory performance, in **Study B** accuracy in a Flanker task provides information on cognitive interference control. Presentation of task-relevant stimuli is realized in Presentation© (version 19, Neurobehavioral Systems Inc., www.neurobs.com). Presentation© offers the opportunity to receive a detailed logfile of the nature and occurrence of all events during the experiment (stimuli, responses, correction of responses). Using e.g. MatlabR2009b (TheMathWorks, Natick, MA), overall accuracy for congruent and incongruent trials will be determined. Participants will obtain time to practice the task. From the practice trials the investigator can judge whether the participant has understood and is able to perform the task. The tasks will be also employed during the intervention session. To avoid fatigue and boredom, the employed stimuli per task will change across the course of the ten sessions. The n-back task for example will use set of different pictures, in the Flanker task arrows and arrow background will change in color. Stimuli during baseline (T2), post (T3) and follow-up (T4) assessment, however, will be identical.

8.2 Assessment of safety

Safety and tolerability of tDCS are assessed by (1) a standardized safety questionnaire (Poreisz et al., 2007) as well as (2) EEG recordings before and after tDCS. Moreover, participants are asked at each visit (following the screening) whether they have experienced AE.

8.3 Adverse events

8.3.1 Adverse event

Adverse events (AE) are defined as any untoward medical occurrence in a clinical investigation subject after contact with the investigational medical device (IMD) which does not necessarily have a causal relationship with this treatment. This includes unfavourable signs and/or symptoms, excacerbation of the investigated condition and newly diagnosed conditions during trial participation.

8.3.1.1 Documentation and evaluation

The investigator must record all AE in the Adverse Event Log provided in the eCRF with information about the following aspects (as described):

Adverse event

AE should be recorded as diagnoses, if available. If not, separate signs and symptoms should be recorded. One diagnosis/symptom should be entered per record. If a patient suffers from the same adverse event more than once and the patient recovers in between the events, the AE should be recorded separately. If an adverse event changes in intensity, a worst-case approach should be used when recording the event, i.e. the highest intensity and the longest duration of the event.

Date and time of onset

The date of onset is the date when the first sign(s) or symptom(s) were first noted. If the adverse event is an abnormal clinically significant laboratory test or outcome of an examination, the onset date is the date the sample was taken or the examination was performed. For pre-existing clinically significant conditions (diagnosed or observed as a result of the screening procedures) becoming worse after IMD application the date of onset is the date the worsening began.

Intensity

The intensity of an adverse event must be classified using the following 3-point scale:

Mild: Awareness of signs or symptoms, but no disruption of usual activity.

Moderate: Event sufficient to affect usual activity (disturbing).

Serious: Inability to work or perform usual activities (unacceptable).

Causal Relationship to IMP

The possibility of whether the IMD application caused the adverse event must be classified as one of the following:

Reasonable possibility:

There is evidence or argument to suggest a causal relationship between the IMD application and the adverse event. The adverse event may occur as part of a physiological or psychological effect of the IMD or may be unpredictable in its occurrence.

No reasonable possibility:

There is no reasonable evidence or argument to suggest a causal relationship between the IMD application and the adverse event.

Action Taken to IMD

The action taken to the IMD application in response to an adverse event must be classified as one of the following:

- No change (application schedule maintained or no action taken)
- Withdrawn
- Interrupted

Other Action Taken

AE requiring therapy must be treated with recognised standards of medical care to protect the health and well-being of the patient. Appropriate resuscitation equipment and medicines must be available to ensure the best possible treatment of an emergency situation. If medication is administered to treat the adverse event, this medication should be entered in the Concomitant Medication Log.

Date of Outcome

The date the patient recovered or died.

Outcome

The outcome of an adverse event must be classified as one of the following:

- Recovered (fully recovered or the condition has returned to the level observed at initiation of trial treatment)
- Recovered with sequelae
- Recovering
- Not recovered
- Fatal

8.3.1.2 Reporting pathways, responsibilities and deadlines

According to national law it is the Sponsor's responsibility to provide AE-listings to Competent Authorities.

8.3.2 Serious adverse events

Any untoward medical occurrence in a clinical investigation experienced by the subject, the person applying the medical device or a third party after contact with the IMD, which does not necessarily have a causal relationship with this treatment, which directly or indirectly could have led or could lead to:

- death,
- a life-threatening situation,
- inpatient hospitalisation or prolongation of existing hospitalisation,
- persistent or significant disability/incapacity or a congenital anomaly/birth defect.

8.3.2.1 Documentation and evaluation

When documenting SAEs the following points are mandatory: SAEs must be documented in the Adverse Event Log in the eCRF as well as on the SAE form. All AEs /SAEs have to be evaluated by the investigator.

8.3.2.2 Reporting pathways, responsibilities and deadlines

Reporting of serious adverse events (SAE) by the Investigator

All SAEs must be reported **immediately** to the Sponsor as soon as they become known to the investigator and not later than within 24 hours of their knowledge of the occurrence of the SAE. The SAE Report Form is included in the ISF, and must be completed and submitted according to the instructions provided on the form. The investigator is responsible for submitting the completed SAE Report. In case initial reports are incomplete, a follow-up report must be sent to the Sponsor within 8 calendar days.

Additional information relevant to the SAE such as hospital records, results from investigations, e.g. laboratory parameter, invasive procedures, scans and x-rays, and autopsy results can be faxed or scanned and e-mailed to the Sponsor. In any case this information must be supplied by the investigator upon request from the Sponsor. On any copies provided, details such as patient's name, address, and hospital ID number should be concealed and instead patient identification numbers should be provided.

Reporting of serious adverse events by the Sponsor

All SAE-Reporting will be performed by the Sponsor according to the national law(s). The detailed processes of SAE-reporting and responsibilities are recorded in a separate SAE-Manual. ***SAEs with a causal relationship*** have to be reported **immediately** by the Sponsor to the Competent Authority.

8.4 Effects

8.4.1 Adverse effects of medical devices

An adverse effect is any untoward and unintentional reaction to the IMD application. An adverse event will be categorised as an adverse effect if a causal relationship between the event and the IMD application is at least deemed possible or cannot be excluded all together. The most common AE associated with the employed device or rather tDCS in general are sensations under the electrodes (tingling, burning, itching), fatigue and redness or skin irritation under the electrode.

8.4.2 Serious adverse device effect (SADE)

A serious adverse effect is any untoward and unintentional reaction to the IMD application, which fulfills the typical criteria for SAE as listed in section 8.3.2. (Serious Adverse Events). A serious adverse event will be categorised as a serious adverse device effect if a causal relationship between the event and the IMD application is at least deemed possible or can not be excluded all together. So far, no SAE have been reported during or subsequent to the use of the employed device or tDCS in general.

8.4.2.1 Reporting pathways, responsibilities and deadlines

SADEs have to be reported immediately by the Sponsor to the Competent Authority as stated above (8.3.2.2).

8.4.3 Incidence

An incident is a malfunction, failure or a modification of the features or performance or an inaccurate label or instruction manual for a medical device, which directly or indirectly caused, may have caused in the past, or may cause in the future, death or a serious aggravation of the state of health of a patient, a user or another person. Incidences are reported to the manufacturer (Neuroelectrics) by the principal investigator.

8.5 Medical history (concomitant diseases)

Conditions, syndromes and symptoms that are present at the screening visit as well as those that have been diagnosed in the past must be documented in the source data as well as in the medical history section of the eCRF as specified in the eCRF. These include chronic conditions, surgical history and mental health history.

8.6 Handling of pregnancies

If a pregnancy occurs at any time after signing the informed consent form, the application of the IMD should be immediately stopped and the Sponsor must be informed using the Pregnancy Form or an SAE Report:

Koordinierungszentrum für Klinische Studien (KKS) Magdeburg
Medical Faculty, Otto-von-Guericke University Magdeburg
Leipziger Str. 44
39120 Magdeburg
Germany
Tel.: +49(0)391 6724780
Fax: +49(0)391 6715898

Note that pregnancy itself is not an SAE, and should not be reported via the eCRF. Contact details for the follow up on the pregnancy should be provided with the report. The Sponsor may request additional pregnancy-specific follow-up information once the pregnancy has been notified.

The mother and the foetus must be followed-up at least until the birth of the infant and one month after the birth of the infant. In general, the follow-up will include the course; duration and the outcome of the pregnancy as well as neonatal health. If a pregnancy results in an abnormal outcome (birth defect/congenital anomaly), this must be reported as an SAE to the Sponsor according to the procedure described in Section **Serious Adverse Event**. Any outcome which the investigator and/or Sponsor consider to be related to the IMP will be treated as an expedited report.

8.7 Measures for protection against imminent danger

The Starstim32 stimulation device already has a number of safety features that limit the possibility of physiologically harmful application of the electric current: the current that can be maximally applied per electrode does not exceed 2 mA (maximal total injected current by all electrodes: 4 mA), stimulus duration cannot be extended over an hour, stimulation sessions need to be pre-programmed, impedances of all electrodes are checked before and during stimulation, and the device can be aborted at any time. Moreover, Starstim will not operate if the contact impedance is above 20 kOhm. This limitation is achieved through the stimulation circuit that is a current sink / source circuit.

Therefore, the current and the voltage are measured constantly to calculate the value of the impedance. The impedance is shown at the software as a bar, which turns green (0 - 5KOhm), orange (5-15K) and red (15-20K). If the impedance measured in one (or more) electrodes is higher than 20KOhm, the device stops the stimulation and warns the user accordingly. Starstim is also constantly assessing the communication between PC (NIC software) and Starstim. If the system encounters the communication between Starstim and the PC is failed, Starstim will disconnect and then the NIC software will inform the user accordingly. Additionally, Starstim incorporates an internal buffer that safeguards the information sent from the device to the PC. So, even if there is a problem of the communication during a certain time, the device will save the data in the internal buffer to ensure that no data (for example an EEG sample) is lost.

8.8 Emergency measures

In case, a serious adverse event occurs during or right after the stimulation, participants will be immediately transfered to emergency care at the participating site. In case of suspected relationship to medical device/treatment unblinding will be performed.

8.9 Data safety monitoring board (DSMB)

A DSMB with three independent members will be installed who will monitor safety data in fixed time intervals. The first meeting will be held after the tenth patient has been included in the trial. When 50% of the participants have been recruited within Study A and Study B, the second meeting will take place. Additional meetings will be organized on demand e.g. in case of emergency, an unscheduled meeting can be performed. If necessary, the DSMB has access to the unblinded data to judge whether the risk-benefit ratio changes throughout the study.

The DSMB will monitor the following data:

- demographic data
- AEs
- SAEs
- safety questionnaire

In case the DSMB deems it necessary to view other original data (e.g. EEG) the data will be provided as well. If the board decides that the safety of participants is significantly endangered, they can recommend stopping the study. The DSMB will prepare protocols of their meetings and provide written recommendations to the Sponsor. Details on the performance of the DSMB are specified in a separate document.

8.10 Other possible study related complications and/or risks

If participants should encounter increased problems in school due to their participation in the study and the need to omit stimulant medication for the duration of the study participation, investigators at each site will offer to issue a letter confirming their participation in the study or to get in touch with school representatives.
In case of allergic reactions to the electrode gel, patients will be referred to a pediatrician or dermatologist.

9 DOCUMENTATION

9.1 Trial master file (TMF)

A trial master file contains all essential documents for a clinical trial that may be subject to regulatory agency supervision. It also contains all relevant information with regards to the qualification of sites where the clinical trial is conducted. All reports during the study will be kept here. It is the responsibility of the Sponsor to keep the records.

9.2 Investigator site file (ISF)

The investigator should maintain the essential trial documents for the conduct of the clinical trial and as required by the applicable regulations. The investigator should take measures to prevent accidental or premature destruction of these documents. The ISF will be provided by the Sponsor.

9.3 Documentation of study data

All documentation has to be transparent and traceable, this also includes any corrections that are made. These should be done in a way that the original entry is still visible, as well as signed and dated. Documentation should only be done by authorized personnel.

The investigator should maintain adequate and accurate source documents and trial records that include all pertinent observations on each of the sites' trial subjects. Source data should be attributable, legible, contemporaneous, original, accurate, and complete. Changes to source data should be traceable, should not obscure the original entry and should be explained if necessary (e.g.,via an audit trail).

The investigator should ensure the accuracy, completeness, legibility, and timeliness of the data reported to the Sponsor in the Case Report Forms (CRF) and in all required reports. Data reported on the CRF, that are derived from source documents, should be consistent with the source documents or the discrepancies should be explained. It is the investigators responsibility that all clinical data will be documented in the CRF after patients visit without any delay.

9.3.1 Source documents

Source Data
Source data are defined as all information in original records and certified copies of original records of clinical findings, observations, or other activities in a clinical trial necessary for the reconstruction and evaluation of the trial. Source data are contained in source documents (original records or certified copies).

Source Documents
Source documents are defined as original documents, data, and records (e.g. hospital records, clinical and office charts, laboratory notes, memoranda, patients' diaries or evaluation checklists, pharmacy intervention records, recorded data from automated instruments, copies or transcriptions certified after verification as being accurate copies, microfiches, photographic negatives, microfilm or magnetic media, x-rays, patient files, and records kept at third parties and at medico-technical departments involved in the clinical trial).

Trial-specific Source Data Requirements
For in- and out-patients treated at the recruiting trial site and allocated to stimulation treatment, the Investigator will indicate in the hospital/medical source records that the patient participates in this trial and the date of obtaining the informed consent. In addition, the following information will also be recorded in the hospital/medical source records for each patient:

- Date of first and last treatment with medical device
- Occurrence of any adverse events/SAEs (including description and duration)

Out-patients from other sources will receive a letter with the respective information for their family physician.

9.3.2 Case report form (CRF)

A validated eCRF software will be used, all study related setups will be done by ZKS Kiel.

Only authorized and trained personnel will get a password/username and is allowed to enter data. All entries and changes will be logged via audit trail. A user's password/username equates to their normal signature and is to be treated as such and not to be shared with other people.

Tas Log Files have to be forwarded by the site directly to the IMIS.

9.4 Data management

Data management of eCRF data by ZKS

Data management of clinical study data will be handled by ZKS Kiel. This includes eCRF construction, training and support of study sites regarding the eCRF system, query management, data cleaning, and database lock. A validated study data base system will be used.

The data management plan (DMP) will be issued by ZKS and will describe all functions, processes, and specifications for data collection, cleaning and validation. The DMP will describe captured methods, who is authorised to enter the data, decisions about ownership of data, source data storage, which data will be transferred (including timing of transfers), the origin and destination of the data and who will have access to the data at all times.

Data management of other data not included in eCRF
All physiological data that are not included in the eCRF (EEG, structural and functional MRI, DTI) will be stored locally at each site and not be handled, processed or archived by the ZKS. In addition, all these data will be saved centrally at the electronic data processing center in the Christian-Albrechts-University in Kiel on a common research server. The data will be transferred to this server after pseudonymisation which will be performed in each site. Each site will keep the pseudonymization code for request in case, for example, of AE or SAE. No personal data will be stored in Kiel. The electronic data processing center takes care of service, regular parallel data storage on a second safety server and of data safety. The data server is protected by VPN surrounding. All sites (Magdeburg, Frankfurt, Coimbra, Barcelona, Lissabon and Kiel) will have access to the server to up- or download pseudonymized physiological data via VPN by Software Package Data Exchange (SPDX). The data can be uploaded by request after permission by the coordinator, leads of WPs and/or the steering committee of the STIPED consortium.

9.5 Archiving

All essential study documents will be archived for ten years.

Trial Master File
The Sponsor will archive the TMF in accordance with applicable regulatory requirements for 10 years.

Investigator Site File
The investigator is responsible for maintaining all the records, which enable the conduct of the trial at the site to be fully understood. The trial documentation including all relevant correspondence should be kept by the investigator for at least 10 years after the completion or discontinuation of the trial, if no further instructions are given by the Sponsor.

The investigator is responsible for the completion and maintenance of the confidential patient identification code which provides the sole link between named patient source records and anonymous CRF data for the Sponsor. The investigator must ensure separate archiving of this Patient Identification Log and signed Informed Consent Documents for at least 10 years after the completion or discontinuation of the trial.

eCRF
The database will be archived electronically by the ZKS Kiel and the KKS Magdeburg for 10 years.

Other data (EEG, MRI, Task Log Files etc.)
All other data such as EEG, MRI, log files from the tasks during V0-V12 will be archived for at least 10 years at the site where they were collected.

10 QUALITY ASSURANCE

10.1 Standard Operating Procedures (SOP)

Standard operating procedures (SOP) aim to minimise variations in outcomes, readings or results. They will be in place for procedures and processes that lend themselves to individualised operation (e.g. application of the Starstim cap, imaging procedures etc.) or individualised interpretation of results or readings.

10.2 Protocol deviations

Deviations from the protocol should not occur. Protocol deviations and their escalation pathways are defined and listed in the monitoring plan. If deviations occur, the investigator must inform the monitor, and the implications of the deviation must be reviewed and discussed. Any deviation must be documented. A log of protocol deviation reports will be maintained by the monitor and periodically forwarded to the Sponsor. Deviation reports and supporting documentation must be kept in the ISF and in the TMF.

10.3 Monitoring

Clinical monitoring of all German sites will be provided by the Center for Clinical Trials (ZKS) Kiel. The site in Portugal will be monitored by a local monitor to ensure that Portuguese regulations regarding the conduct of clinical trials are adhered to. In order to maintain consistency with regards to monitoring procedures, the ZKS Kiel will survey all monitoring reports from Portugal and, if necessary, take corrective action, before forwarding them to the sponsor.

The monitor will contact and visit the trial sites periodically to ensure adherence to applicable regulatory and ethical requirements, the protocol, Good Clinical Practice (GCP), SOPs and, maintenance of trial-related source records, completeness, accuracy, and verifiability of eCRF entries compared to source data. The investigator will permit the monitor direct access to all source data, including medical records, and/or documents in order to facilitate data verification. The investigator will co-operate with the monitor to ensure that any discrepancies that may be identified are resolved. The investigator is expected to be able to meet the monitor during these visits. A source data verification (SDV) will be performed at each visit to ensure data integrity.

The SDV process and definition of key variables to be monitored as well as the extent and frequency of the monitoring visits will be described in detail in the Monitoring Plan for the trial.

10.4 Audits and inspections

It is the Sponsor's right to conduct audits at trial sites and other departments or institutions involved in the clinical trial for the purpose of quality assurance. The main purposes of an audit or inspection are to assess compliance with the trial protocol and the principles of GCP including the Declaration of Helsinki and all other relevant regulations.

Federal, local as well as foreign authorities have the right to conduct inspections at any point in the clinical trial at the trial site, the Sponsor's and/or contract research organisation's (CRO's) facilities or at other establishments deemed appropriate by the regulatory authority.

The investigator will make all the trial-related source data and records available at any time to quality assurance auditor(s) or to domestic/foreign regulatory inspectors who may audit/inspect the trial. The investigator should notify the Sponsor without any delay of any inspection by a regulatory authority.

11 STATISTICS

All statistical methods described in this chapter refer to clinical variables only (i.e., documented in the eCRF). The IMIS will analyze these clinical variables but not other data. Thus, data from all substudies (e.g. raw MRI or EEG data, qualitative data from substudy 1) are excluded from this analysis section. In particular, the IMIS will not perform additional analyses that are only relevant for doctoral theses. However, statistical guidance may be possible in some cases.

A detailed statistical analysis plan (SAP) will be written prior to any data analysis that describes all planned statistical analyses.

11.1 Sample size calculation

The sample size for **Study A** and **Study B** is calculated separately for each study using a two-sided t-test (significance level α=0.05) with a power 1-β=80% based on a medium expected effect size of d=0.6. Previous studies in ADHD have shown similar or larger effect sizes for tDCS. This result leads

to a required sample size of 45 patients per group (G*Power3.1); assuming 10% drop-out, the total sample size consists of 200 randomized patients (100 patients for each study). In **Study A**, 50 patients will be allocated randomly to Arm A, whereas the remaining 50 patients will be randomized to Arm B. The same procedure will be repeated for **Study B**.

11.2 Randomisation

Randomisation will be carried out with respect to the randomisation protocols. Each patient will be allocated randomly to one of the two studies (**Study A** or **Study B**) and to one of the two treatment conditions (anodal tDCS stimulation or sham stimulation). Randomisation will be stratified for the variables clinical center (Coimbra, Frankfurt, Kiel/ZIP, Kiel/UKSH or Magdeburg), and gender (male or female). Experimental conditions will be block randomized. Each block will have a length of four and, thus, will assign a total of four patients to the different treatment conditions (**Study A** + tDCS, **Study A** + sham, **Study B** + tDCS, and **Study B** + sham). Block randomization will be done with the electronic randomization tool BiAS for Windows (version 11, 2016). Randomisation will be performed by IMIS/ZKS.

11.3 Statistical methods

11.3.1 Objectives

The current sham-controlled study aims to investigate effect sizes of changes in neuropsychological performance and study safety and tolerability of multi-channel anodal tDCS targeting the left dorsolateral prefrontal cortex (dlPFC, **Study A**) or the right inferior frontal gyrus (rIFG, **Study B**) in children and adolescents with ADHD.

Three secondary objectives will be examined. First, the potential benefit of multi-channel anodal tDCS targeting left dlPFC (**Study A**) or the rIFG (**Study B**) for clinical measures as well as quality of life in children and adolescents with ADHD will be investigated. Second, individual factors derived from MRI and/or EEG measures as well as clinical measures predicting general responsivity to stimulation or differential responsivity to different tDCS settings will be identified in a substudy. Third, expectations and concerns of parents and participants towards tDCS will be assessed and evaluated in a substudy.

11.3.2 Analysis population

The primary analysis will be based on the intention-to-treat (ITT) set, including all randomized patients irrespective of the amount of treatment actually received. A per-protocol (PP) analysis will be performed as sensitivity analysis. All participants will be asked to participate in the end-of-treatment and follow-up assessment, even if they drop out of treatment, to minimize the number of those who are lost to follow-up. Deviations from the protocol that lead to exclusion from the PP-set will be detailed in the SAP.

11.3.3 Data analysis

Primary Outcome

We will test for a difference in the primary outcome measure at T3 (Visit 11; **Study A**: overall accuracy in an *n-back* task at the end of of the intervention; **Study B:** overall accuracy in the *Flanker* task at the end of of the intervention) using an analysis of covariance with covariates center, sex, puberty status, age, IQ, and the baseline value of accuracy in the respective tasks at T2 (Visit 0). Emphasis will be on the 95% confidence intervals of the effect size estimates; additionally, p-values will be calculated. Analyses will be performed separately for **Study A** and **Study B**.

Secondary Outcomes

Secondary outcomes relating to further clinical measures as well as quality of life and the additional outcomes at the follow-up assessment (T4, Visit 12) will be analyzed in a similar way. Analysis of functional brain related outcome measures and all outcomes form substudies are the responsibility of the investigators named in 2.3.

Handling of missing data

In the primary analysis of the primary endpoint, we will use multiple imputations to account for missing data. Multiple imputations are currently the preferred method for dealing with missing values (Graham et al., 2003).

Sensitivity analyses will be performed to investigate the potential impact of missing data, in particular, by using data of both the end-of-treatment and follow-up assessments in a mixed-effects model repeated measures analysis, and a complete case analysis.

Statistical Software
Statistical analyses will be performed using the statistical software *IBM SPSS Statistics* which will be used for descriptive statistics and all main analyses.

11.3.4 Interim analysis
There will be no interim analysis.

12 REPORTS AND PUBLICATIONS

12.1 Reports

12.1.1 Safety reports
Safety reports will be reported by the Sponsor according to national requirements. Quarterly reports of SAEs have to be submitted to the Competent Authority in Germany. In Portugal, the Competent Authority receives annual safety reports.

12.1.2 Final report
It is the Sponsor’s responsibility to inform the authorities and ethics committes about the end of the clinical trial according to national law (Germany: at least 90 days after regular ending of the trial; after interruption of the trial: at least 15 days under provision of reasons).

A final report to summarize the conduct and results will be prepared at the end of the study by the coordinating investigator (CI) and submitted to all relevant institutions (Competent Authorities and ethics committees). The report will contain the statistical analysis and the medical assessment of the results. National laws have to be taken into account (Germany: reports will have to be submitted at least one year after trial completion).

12.2 Publications

All results will be published according to rules defined in the consortium agreement (CA) of STIPED and in agreement with the Sponsor/coordinator of the clinical trials and the Steering Committee of STIPED. Contributions of all participating parties will be acknowledged and order of authors will be decided according to good scientific practice

13 ETHICAL, LEGAL AND ADMINISTRATIVE ASPECTS

13.1 Ethics committees and authorities

The study will be conducted in accordance with the study protocol, the principles of the Declaration of Helsinki, as well as the Good Clinical Practice, relevant national laws and applicable federal and local regulatory requirements for data privacy protection. All essential documents will be archived at each participating site.

The protocol and local Informed Consent (IC) forms must be reviewed and approved in writing by an Independent Ethic Committee (IEC) and the federal authority prior to the initiation of subject recruitment. Each of the IEC and the federal authorities must be notified of all subsequent protocol amendments. According to German law all local authorities and institutions will be notified.

13.2 Insurance

On behalf of the Sponsor an insurance policy (*in Germany*: according to § 20 (1) Nr. 9 und (3) MPG, *in Portugal:* according to Decree-Law 145/2009, 17 June (Transposition of Directives 93/42/CEE, 90/385/CEE and2007/47/CE) for all participants in this clinical trial has been taken out with the following provider:

Name of Insurance Company: HDI Global SE
Insurance Number: 57 010330 03010
Address: Riethorst 2, 30659 Hannover, Germany
Telephone: +49 (0) 511 645-0
Fax: +49 (0) 511 645-4545

One copy of the insurance police and the conditions and terms of the insurance will be handed out to the participants and their custodians, the other copy remains in the ISF. This process will be documented in the source data.

13.3 Informed consent

Before the first visit, all participants and their families will receive comprehensive written information about the aim, procedure and potential risks of the study, data protection and their right to withdraw at any time from their participation without any impacts on their care. Separate, age-appropriate information will be provided for participants and their parents/custodians. During the first visit, a physician will verbally inform participants and parents/custodians about the study and will allow ample time to discuss questions and concerns. In case participants and parents/custodians feel sufficiently informed and are willing to take part in the study, they will receive and sign an assent (minors) or consent form (parents/custodians). In general, consent forms need to be signed by both parents.
The written information as well as the assent and consent form was translated to Portugese by the professional provider CSOFT International Ltd.

13.4 Data protection

All terms of national data protection regulations have to be obeyed. All materials/documents/data will be pseudonymised before leaving the participating site for scientific analysis.
Participants and their custodians will be adequately informed about the dissemination of personal data. Subjects who will not consent to this dissemination will not be included into the clinical trial.

Parents and participants are moreover asked to give their consent for the analysis of the collected data in the described sub-studies (see 2.3).

13.5 Study protocol

13.5.1 Adherence

The protocol must be strictly adhered to. Each deviation has to be documented and justified in written form.

13.5.2 Amendments

Any change to this protocol will be documented in a protocol amendment and agreed upon by the investigators, trial statistican and the Sponsor prior to its implementation.

Protocol amendments will be submitted for notification of EC and federal authority, in accordance with local regulations. An approval by the EC and federal authority is required for a substantial amendment, e.g. one which could affect the safety of the subjects, or which entails a change to the scope/design of the trial.

13.6 Responsibilities

The Sponsor is responsible for the vigilance and control of the clinical trial according to §3 paragraph 23-25, §§ 20-24 MPG and DIN EN ISO 14155:2011. The Sponsor supports the investigator in the administration of the received funding throughout the course of the clinical trial.

The investigator is indebted to consider all current legislation considered relevant by the Sponsor for conducting the clinical trial. This concerns adherence to the MPG, MPKPV, MPSV, DIN EN ISO 14155:2011, European Guidelines 2007/47/EG including implementary regulations (90/385/EWG annex 7 Nr. 2.3, 93/42/EWG annex X Nr. 2.3 as well as the MEDDEV-guidelines 2.7.1.

The investigator is responsible for ensuring adherence to the study protocol and the PZ-SOPs of the Sponsor at their participating site and for communicating amendments. The investigator is obligated to guarantee that all members of the investigating team are familiar with the Investigator's brochure and

are trained in the use of the medical device. Moreover, the investigator takes care that the storage as well as the accountability for the medical device is done according to good clinical practice.

The sponsor is in charge of providing and maintaining insurance protection for all participants of the study. The sponsor and the coordinating investigator will perform an ongoing risk-benefit evaluation of the study. It is every investigator's duty to support audits initiated by the Sponsor.

13.7 Study registration

According to the Declaration of Helsinki and ICMJE guidelines for publications, this trial will be registered before start of the trial by the Sponsor in a public, clinical trials registry, namely the German Clinical Trials Register (DRKS). The DRKS is an approved primary register in the WHO network. It is the responsibility of the Sponsor to register the trial in the DRKS before the first patient is included.

14 FINANCING

Financing of the Trial
This trial is a part of the EU-Project STIPED (Horizon2020) and is therefore financed through the Grant Agreement Nr. 731827. The multi-channel tDCS device employed in the proposed study is provided by Neuroelectrics as part of their obligations within the STIPED consortium.

This trial does not receive financial support from the industry.

Compensation of trial sites
All sites receive conpensation for recruitment of patients as instalments as described in the Annex of Consortium Agreement.

Reimbursement for subjects
At the German sites, children and adolescents will receive a reimbursement in form of shopping vouchers equaling the value of 10€ per hour for the screening and all visits except the intervention visits (V1 - V10). Even though we do not anticipate from our previous experience that children and adolescents would feel obliged to return to the intervention visits if they felt uncomfortable during the intervention, we wanted to exclude this possibility. The screening, V0, V11, and V12 will last about two hours, thus participants will receive an overall sum of 80€, if they do not participate in the MRI session. MRI sessions will be reimbursed with 10€.

In Coimbra, it is not allowed to financially reimburse study participants, thus, they will not receive any payment for their participation in the study. However, to acknowledge the time and effort spent by the families, participants and their families are reimbursed for costs with traveling, meals and accommodation through payment of daily allowances.

Compensation of travel expenses for subjects
Parents will obtain reimbursement of their travel costs at all sites according to the local specifications.

15 LITERATURE

Ajzen I (1991) The theory of planned behavior. Organizational Behavior and Human Decision Processes 50:179-211.

Alon G, Syron SC, Smith GV (1998) Is transcranial electrical stimulation (TCES) a safe intervention for children with cerebral palsy? Journal of Neurologic Rehabilitation 12:65-71.

American-Psychiatric-Association (2013) Diagnostic and Statistical Manual of Mental Disorders: American Psychiatric Association.

Andrade AC, Magnavita GM, Allegro JV, Neto CE, Lucena Rde C, Fregni F (2014) Feasibility of transcranial direct current stimulation use in children aged 5 to 12 years. Journal of child neurology 29:1360-1365.

Aron AR, Poldrack RA (2005) The cognitive neuroscience of response inhibition: relevance for genetic research in attention-deficit/hyperactivity disorder. Biological psychiatry 57:1285-1292.

Bikson M et al. (2016) Safety of Transcranial Direct Current Stimulation: Evidence Based Update 2016. Brain stimulation 9:641-661.

Boggio PS, Ferrucci R, Rigonatti SP, Covre P, Nitsche M, Pascual-Leone A, Fregni F (2006) Effects of transcranial direct current stimulation on working memory in patients with Parkinson's disease. Journal of the neurological sciences 249:31-38.

Bokor G, Anderson PD (2014) Attention-Deficit/Hyperactivity Disorder. Journal of pharmacy practice 27:336-349.

Bourzac K (2016) Neurostimulation: Bright sparks. Nature 531:S6-8.

Bradley MM, Lang PJ (1994) Measuring emotion: the Self-Assessment Manikin and the Semantic Differential. J Behav Ther Exp Psychiatry 25:49-59.

Breitling C, Zaehle T, Dannhauer M, Bonath B, Tegelbeckers J, Flechtner H-H, Krauel K (2016) Improving interference control in ADHD patients with transcranial direct current stimulation (tDCS). Frontiers in Cellular Neuroscience 10.

Brunoni AR, Vanderhasselt MA (2014) Working memory improvement with non-invasive brain stimulation of the dorsolateral prefrontal cortex: a systematic review and meta-analysis. Brain and cognition 86:1-9.

Brunoni AR, Amadera J, Berbel B, Volz MS, Rizzerio BG, Fregni F (2011) A systematic review on reporting and assessment of adverse effects associated with transcranial direct current stimulation. The international journal of neuropsychopharmacology 14:1133-1145.

Brunoni AR, Valiengo L, Baccaro A, Zanao TA, de Oliveira JF, Goulart A, Boggio PS, Lotufo PA, Bensenor IM, Fregni F (2013) The sertraline vs. electrical current therapy for treating depression clinical study: results from a factorial, randomized, controlled trial. JAMA psychiatry 70:383-391.

Bunge SA, Dudukovic NM, Thomason ME, Vaidya CJ, Gabrieli JD (2002) Immature frontal lobe contributions to cognitive control in children: evidence from fMRI. Neuron 33:301-311.

Clavenna A, Bonati M (2014) Safety of medicines used for ADHD in children: a review of published prospective clinical trials. Archives of disease in childhood 99:866-872.

Cohen Kadosh R, Soskic S, Iuculano T, Kanai R, Walsh V (2010) Modulating neuronal activity produces specific and long-lasting changes in numerical competence. Current biology : CB 20:2016-2020.

Cosmo C, Ferreira C, Miranda JG, do Rosario RS, Baptista AF, Montoya P, de Sena EP (2015) Spreading Effect of tDCS in Individuals with Attention-Deficit/Hyperactivity Disorder as Shown by Functional Cortical Networks: A Randomized, Double-Blind, Sham-Controlled Trial. Frontiers in psychiatry 6:111.

Delmo C, Weiffenbach O, Gabriel M, Bölte S, Marchio E, Poustka F (2000) Fragebogen für Affektive Störungen und Schizophrenie für Kinder im Schulalter (6-18 Jahre). Frankfurt: Klinik für Psychiatrie und Psychotherapie des Kindes- und Jugendalters.

Ditye T, Jacobson L, Walsh V, Lavidor M (2012) Modulating behavioral inhibition by tDCS combined with cognitive training. Experimental brain research 219:363-368.

Durston S, Hulshoff Pol HE, Schnack HG, Buitelaar JK, Steenhuis MP, Minderaa RB, Kahn RS, van Engeland H (2004) Magnetic resonance imaging of boys with attention-deficit/hyperactivity disorder and their unaffected siblings. Journal of the American Academy of Child and Adolescent Psychiatry 43:332-340.

Elsner B, Kugler J, Pohl M, Mehrholz J (2016) Transcranial direct current stimulation (tDCS) for improving activities of daily living, and physical and cognitive functioning, in people after stroke. The Cochrane database of systematic reviews 3:Cd009645.

Evans SW, Owens JS, Bunford N (2014) Evidence-based psychosocial treatments for children and adolescents with attention-deficit/hyperactivity disorder. Journal of clinical child and adolescent psychology : the

official journal for the Society of Clinical Child and Adolescent Psychology, American Psychological Association, Division 53 43:527-551.

Fredriksen M, Dahl AA, Martinsen EW, Klungsoyr O, Faraone SV, Peleikis DE (2014) Childhood and persistent ADHD symptoms associated with educational failure and long-term occupational disability in adult ADHD. Attention deficit and hyperactivity disorders 6:87-99.

Gajria K, Lu M, Sikirica V, Greven P, Zhong Y, Qin P, Xie J (2014) Adherence, persistence, and medication discontinuation in patients with attention-deficit/hyperactivity disorder - a systematic literature review. Neuropsychiatric disease and treatment 10:1543-1569.

Gandiga PC, Hummel FC, Cohen LG (2006) Transcranial DC stimulation (tDCS): a tool for double-blind sham-controlled clinical studies in brain stimulation. Clinical neurophysiology : official journal of the International Federation of Clinical Neurophysiology 117:845-850.

Göder R, Baier PC, Beith B, Baecker C, Seeck-Hirschner M, Junghanns K, Marshall L (2013) Effects of transcranial direct current stimulation during sleep on memory performance in patients with schizophrenia. Schizophrenia Research 144:153-154.

Graham JW, Cumsille PE, Elek-Fisk E (2003) Methods for handling missing data. In: Research Methods in Psychology (Schinka JA, Velicer WF, eds), pp 87-114. New York: John Wiley & Sons.

Heimrath K, Sandmann P, Becke A, Muller NG, Zaehle T (2012) Behavioral and electrophysiological effects of transcranial direct current stimulation of the parietal cortex in a visuo-spatial working memory task. Frontiers in psychiatry / Frontiers Research Foundation 3:56.

Hsu TY, Tseng LY, Yu JX, Kuo WJ, Hung DL, Tzeng OJ, Walsh V, Muggleton NG, Juan CH (2011) Modulating inhibitory control with direct current stimulation of the superior medial frontal cortex. NeuroImage 56:2249-2257.

Iyer MB, Mattu U, Grafman J, Lomarev M, Sato S, Wassermann EM (2005) Safety and cognitive effect of frontal DC brain polarization in healthy individuals. Neurology 64:872-875.

Jacobson L, Javitt DC, Lavidor M (2011) Activation of inhibition: diminishing impulsive behavior by direct current stimulation over the inferior frontal gyrus. Journal of cognitive neuroscience 23:3380-3387.

Kamp-Becker I, Schroder J, Muehlan H, Remschmidt H, Becker K, Bachmann CJ (2011) Health-related quality of life in children and adolescents with autism spectrum disorder. Zeitschrift fur Kinder- und Jugendpsychiatrie und Psychotherapie 39:123-131.

Kaufmann J, Birmaher B, Brent D, Rao U, Flynn C, Moreci P, Williamson D, Ryan N (1997) Schedule for affective disorders and schizophrenia for school-age children - Present and lifetime version (K-SADS-PL): Initial reliability and validity data. Journal of the American Academy of Child and Adolescent Psychiatry 36:980-988.

Keeser D, Meindl T, Bor J, Palm U, Pogarell O, Mulert C, Brunelin J, Moller HJ, Reiser M, Padberg F (2011) Prefrontal transcranial direct current stimulation changes connectivity of resting-state networks during fMRI. J Neurosci 31:15284-15293.

Kekic M, Boysen E, Campbell IC, Schmidt U (2016) A systematic review of the clinical efficacy of transcranial direct current stimulation (tDCS) in psychiatric disorders. Journal of psychiatric research 74:70-86.

Kern JK, Geier DA, Sykes LK, Geier MR, Deth RC (2015) Are ASD and ADHD a Continuum? A Comparison of Pathophysiological Similarities Between the Disorders. Journal of attention disorders 19:805-827.

Kim YS, Cheon KA, Kim BN, Chang SA, Yoo HJ, Kim JW, Cho SC, Seo DH, Bae MO, So YK, Noh JS, Koh YJ, McBurnett K, Leventhal B (2004) The reliability and validity of Kiddie-Schedule for Affective Disorders and Schizophrenia-Present and Lifetime Version- Korean version (K-SADS-PL-K). Yonsei medical journal 45:81-89.

Krause B, Cohen Kadosh R (2013) Can transcranial electrical stimulation improve learning difficulties in atypical brain development? A future possibility for cognitive training. Developmental cognitive neuroscience 6:176-194.

Krishnan C, Santos L, Peterson MD, Ehinger M (2015) Safety of noninvasive brain stimulation in children and adolescents. Brain stimulation 8:76-87.

Kuo MF, Paulus W, Nitsche MA (2014) Therapeutic effects of non-invasive brain stimulation with direct currents (tDCS) in neuropsychiatric diseases. NeuroImage 85 Pt 3:948-960.

Lampert T, Muters S, Stolzenberg H, Kroll LE (2014) [Measurement of socioeconomic status in the KiGGS study: first follow-up (KiGGS Wave 1)]. Bundesgesundheitsblatt, Gesundheitsforschung, Gesundheitsschutz 57:762-770.

Lee YS, Han DH, Lee JH, Choi TY (2010) The effects of methylphenidate on neural substrates associated with interference suppression in children with ADHD: A preliminary study using event related fMRI. Psychiatry investigation 7:49-54.

Lopez-Alonso V, Cheeran B, Rio-Rodriguez D, Fernandez-Del-Olmo M (2014) Inter-individual variability in response to non-invasive brain stimulation paradigms. Brain stimulation 7:372-380.

Luks TL, Oliveira M, Possin KL, Bird A, Miller BL, Weiner MW, Kramer JH (2010) Atrophy in two attention networks is associated with performance on a Flanker task in neurodegenerative disease. Neuropsychologia 48:165-170.

Mannuzza S, Klein RG, Moulton JL, 3rd (2003) Persistence of Attention-Deficit/Hyperactivity Disorder into adulthood: what have we learned from the prospective follow-up studies? Journal of attention disorders 7:93-100.

Mattai A, Miller R, Weisinger B, Greenstein D, Bakalar J, Tossell J, David C, Wassermann EM, Rapoport J, Gogtay N (2011) Tolerability of transcranial direct current stimulation in childhood-onset schizophrenia. Brain stimulation 4:275-280.

McDermott MS, Oliver M, Iverson D, Sharma R (2016) Effective techniques for changing physical activity and healthy eating intentions and behaviour: A systematic review and meta-analysis. British journal of health psychology 21:827-841.

McEachan RRC, Conner M, Taylor NJ, Lawton RJ (2011) Prospective prediction of health-related behaviours with the Theory of Planned Behaviour: a meta-analysis. Health Psychology Review 5:97-144.

Moliadze V, Lyzhko E, Boecher L, Freitag C, Siniatchkin M (2014) Neuronal mechanisms of error monitoring in motivational context in healthy children and adolescents. In: OHBM 2014. Hamburg.

Moliadze V, Andreas S, Lyzhko E, Schmanke T, Gurashvili T, Freitag CM, Siniatchkin M (2015) Ten minutes of 1mA transcranial direct current stimulation was well tolerated by children and adolescents: Self-reports and resting state EEG analysis. Brain Res Bull 119:25-33.

Munz MT, Prehn-Kristensen A, Thielking F, Molle M, Goder R, Baving L (2015) Slow oscillating transcranial direct current stimulation during non-rapid eye movement sleep improves behavioral inhibition in attention-deficit/hyperactivity disorder. Front Cell Neurosci 9:307.

Muszkat D, Polanczyk GV, Dias TG, Brunoni AR (2016) Transcranial Direct Current Stimulation in Child and Adolescent Psychiatry. Journal of child and adolescent psychopharmacology.

Nitsche MA, Paulus W (2000) Excitability changes induced in the human motor cortex by weak transcranial direct current stimulation. The Journal of physiology 527 Pt 3:633-639.

Nitsche MA, Paulus W (2011) Transcranial direct current stimulation--update 2011. Restorative neurology and neuroscience 29:463-492.

Nitsche MA, Liebetanz D, Lang N, Antal A, Tergau F, Paulus W (2003) Safety criteria for transcranial direct current stimulation (tDCS) in humans. Clinical neurophysiology : official journal of the International Federation of Clinical Neurophysiology 114:2220-2222; author reply 2222-2223.

Nitsche MA, Niehaus L, Hoffmann KT, Hengst S, Liebetanz D, Paulus W, Meyer BU (2004) MRI study of human brain exposed to weak direct current stimulation of the frontal cortex. Clinical neurophysiology : official journal of the International Federation of Clinical Neurophysiology 115:2419-2423.

Nitsche MA, Cohen LG, Wassermann EM, Priori A, Lang N, Antal A, Paulus W, Hummel F, Boggio PS, Fregni F, Pascual-Leone A (2008) Transcranial direct current stimulation: State of the art 2008. Brain stimulation 1:206-223.

Oldfield RC (1971) The assessment and analysis of handedness: the Edinburgh inventory. Neuropsychologia 9:97-113.

Polanczyk G, de Lima MS, Horta BL, Biederman J, Rohde LA (2007) The worldwide prevalence of ADHD: a systematic review and metaregression analysis. The American journal of psychiatry 164:942-948.

Polania R, Nitsche MA, Paulus W (2010) Modulating functional connectivity patterns and topological functional organization of the human brain with transcranial direct current stimulation. Human brain mapping 32:1236-1249.

Poreisz C, Boros K, Antal A, Paulus W (2007) Safety aspects of transcranial direct current stimulation concerning healthy subjects and patients. Brain Res Bull 72:208-214.

Prehn-Kristensen A, Munz M, Goder R, Wilhelm I, Korr K, Vahl W, Wiesner CD, Baving L (2014) Transcranial oscillatory direct current stimulation during sleep improves declarative memory consolidation in children with attention-deficit/hyperactivity disorder to a level comparable to healthy controls. Brain stimulation 7:793-799.

Ravens-Sieberer U, Europe KG (2006) The Kidscreen questionnaires: quality of life questionnaires for children and adolescents; handbook: Pabst Science Publ.

Ravens-Sieberer U, Erhart M, Wille N, Bullinger M (2008) Health-related quality of life in children and adolescents in Germany: results of the BELLA study. European child & adolescent psychiatry 17 Suppl 1:148-156.

Ravens-Sieberer U, Gosch A, Rajmil L, Erhart M, Bruil J, Duer W, Auquier P, Power M, Abel T, Czemy L, Mazur J, Czimbalmos A, Tountas Y, Hagquist C, Kilroe J, Group EK (2005) KIDSCREEN-52 quality-of-life measure for children and adolescents. Expert Review of Pharmacoeconomics & Outcomes Research 5:353-364.

Rubia K, Halari R, Cubillo A, Smith AB, Mohammad AM, Brammer M, Taylor E (2011) Methylphenidate normalizes fronto-striatal underactivation during interference inhibition in medication-naive boys with attention-deficit hyperactivity disorder. Neuropsychopharmacology : official publication of the American College of Neuropsychopharmacology 36:1575-1586.

Rutter M, Bailey A, Lord C (2003) Social Communication Questionnaire. Los Angeles: Western Psychological Services.

Schneider HD, Hopp JP (2011) The use of the Bilingual Aphasia Test for assessment and transcranial direct current stimulation to modulate language acquisition in minimally verbal children with autism. Clinical linguistics & phonetics 25:640-654.

Shaw P, Eckstrand K, Sharp W, Blumenthal J, Lerch JP, Greenstein D, Clasen L, Evans A, Giedd J, Rapoport JL (2007) Attention-deficit/hyperactivity disorder is characterized by a delay in cortical maturation. Proc Natl Acad Sci U S A 104:19649-19654.

Siniatchkin M, Glatthaar N, von Muller GG, Prehn-Kristensen A, Wolff S, Knochel S, Steinmann E, Sotnikova A, Stephani U, Petermann F, Gerber WD (2012) Behavioural treatment increases activity in the cognitive neuronal networks in children with attention deficit/hyperactivity disorder. Brain topography 25:332-344.

Soff C, Sotnikova A, Christiansen H, Becker K, Siniatchkin M (2017) Transcranial direct current stimulation improves clinical symptoms in adolescents with attention deficit hyperactivity disorder. Journal of neural transmission (Vienna, Austria : 1996) 124:133-144.

Sotnikova A, Soff C, Tagliazucchi E, Becker K, Siniatchkin M (2017) Transcranial Direct Current Stimulation Modulates Neuronal Networks in Attention Deficit Hyperactivity Disorder. Brain topography.

Sowell ER, Thompson PM, Welcome SE, Henkenius AL, Toga AW, Peterson BS (2003) Cortical abnormalities in children and adolescents with attention-deficit hyperactivity disorder. Lancet 362:1699-1707.

Stagg CJ, Nitsche MA (2011) Physiological basis of transcranial direct current stimulation. The Neuroscientist : a review journal bringing neurobiology, neurology and psychiatry 17:37-53.

Tadini L, El-Nazer R, Brunoni AR, Williams J, Carvas M, Boggio P, Priori A, Pascual-Leone A, Fregni F (2011) Cognitive, mood, and electroencephalographic effects of noninvasive cortical stimulation with weak electrical currents. The journal of ECT 27:134-140.

Tortella G, Casati R, Aparicio LV, Mantovani A, Senco N, D'Urso G, Brunelin J, Guarienti F, Selingardi PM, Muszkat D, Junior Bde S, Valiengo L, Moffa AH, Simis M, Borrione L, Brunoni AR (2015) Transcranial direct current stimulation in psychiatric disorders. World journal of psychiatry 5:88-102.

Varga ET, Terney D, Atkins MD, Nikanorova M, Jeppesen DS, Uldall P, Hjalgrim H, Beniczky S (2011) Transcranial direct current stimulation in refractory continuous spikes and waves during slow sleep: a controlled study. Epilepsy research 97:142-145.

Watzlawik M (2009) Assessing pubertal status with the Pubertal Development Scale: First steps towards an evaluation of a German translation. Diagnostica 55:55-65.

Weiss RH (2008) Grundintelligenztest Skala 2 - Revision (CFT 20-R). Goettingen: Hogrefe.

Woods AJ, Antal A, Bikson M, Boggio PS, Brunoni AR, Celnik P, Cohen LG, Fregni F, Herrmann CS, Kappenman ES, Knotkova H, Liebetanz D, Miniussi C, Miranda PC, Paulus W, Priori A, Reato D, Stagg C, Wenderoth N, Nitsche MA (2016) A technical guide to tDCS, and related non-invasive brain stimulation tools. Clinical neurophysiology : official journal of the International Federation of Clinical Neurophysiology 127:1031-1048.

Zaehle T, Rach S, Herrmann CS (2010) Transcranial alternating current stimulation enhances individual alpha activity in human EEG. PloS one 5:e13766.

Zaehle T, Sandmann P, Thorne JD, Jancke L, Herrmann CS (2011) Transcranial direct current stimulation of the prefrontal cortex modulates working memory performance: combined behavioural and electrophysiological evidence. BMC neuroscience 12:2.

Zhu DC, Zacks RT, Slade JM (2010) Brain activation during interference resolution in young and older adults: an fMRI study. NeuroImage 50:810-817.

Overview of protocol changes

(clinical trial E-StimADHD, EUDAMED-Nr: CIV-17-09-021424)

The following section gives an overview over the changes that were made in the subsequent three versions of the original protocol. All changes were minor and mostly addressed organizational or administrative issues.

Amendment 1 Protocol 1.1, March 4th, 2020

Amendment 2 Protocol 1.2, June 2nd, 2020

Amendment 3 Protocol 1.3, June 10th, 2021

Amendment 1 Protocol 1.1, March 4th, 2020

1. Michael Siniatchkin was appointed head of the Department of Child and Adolescent Psychiatry and Psychotherapy, Evangelical Hospital Bethel in Bielefeld, Germany. The former site (Institute of Medical Psychology and Medical Sociology, University of Kiel, Germany Kiel) was closed and substituted by the Department of Child and Adolescent Psychiatry and Psychotherapy, Evangelical Hospital Bethel in Bielefeld, Germany.
2. The planned recruitment period was extended from Q3/2019 to Q3/2020
3. The following changes/additions to the protocol were required after an inspection of the sponsor by a representative of the State authority (Saxony-Anhalt, Germany).

   Request (1): "The central details of the experiment must be fully and clearly defined in the protocol in accordance with Section 20 (1) sentence 4 no. 6 MPG in conjunction with DIN EN ISO 14155 Annex A, points A2 and A 6.2 must be defined completely and clearly in the protocol. An I/t diagram (…) must be attached to the written descriptions for better understanding."
   The required information, particularly a more detailed description of the sham stimulation, was added to the protocol (p. 34) and an I/t diagram (Figure 5A and B, p. 35) was inserted:
   Study related procedures (V) Intervention: tDCS (p.34):
   - Real stimulation: Current intensity per electrode = 0.5 mA and total injected current = 1.0 mA, stimulation duration 20 min, additionally 30 s ramp up in the beginning and 30 s ramp down in the end (see Figure 5A).
   - Control intervention: The sham stimulation consists of two short stimulation intervals at the beginning and the end of the regular stimulation duration (see Figure 5B).
     - Beginning: 30 s ramp up to a current intensity of 0.5 mA per electrode, 5 s stimulation, 30 s ramp down
     - End: 30 s ramp up to a current intensity of 0.5 mA per electrode, 30 s ramp down
   - Stimulation type: tDCS
     - Target: left dorsolateral prefrontal cortex (A), right inferior frontal gyrus (B)
     - Electric field in target area: 0.25 V/m excitatory
     - Electrode type: PITRODE (3.14 cm2 Ag/AgCl/gel electrode)
     - Maximum current at any electrode: 0.5 mA
     - I/A ratio: 0.159 mA/cm2
     - Total injected current: 1.0 mA

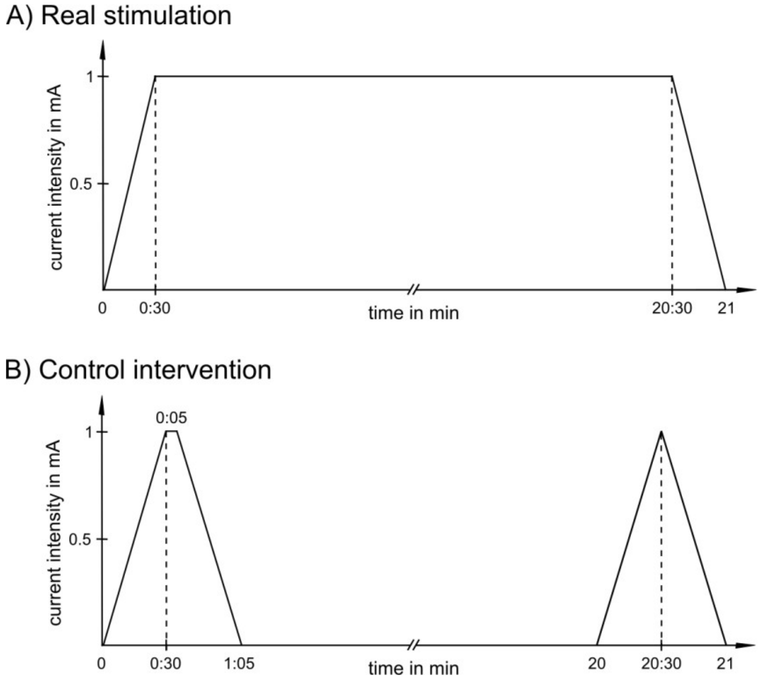

Figure 5: Diagram of the current intensity applied over the time course of stimulation during A) the real stimulation and B) the control intervention.

Request (2): “All accessories used in the procedure must be reproducibly specified.“
The electrode types NG Geltrode NE032 and NG Pistim NE029 used in the study were described in more detail in the study protocol (6.1.) and in the Investigator’s brochure:
Device description (p.25): A hybrid tCS & EEG electrode is used at all channels, where tDCS is applied. This electrode is called NG Pistim (NE029) and is unique due to its dual functionality: it works for stimulation and for EEG monitoring. Thus, it is the ideal electrode when EEG monitoring is required in a stimulation session. Further, due to its reduced size, it is perfect for focal multi-electrode stimulation experiments. The NG Pistim provides a 3.14 cm2 circular contact area and a rear-fill aperture for gel supply. It is based on a sintered Ag/AgCl pellet of 12 mm diameter. On channels, where EEG is recorded but no tDCS is applied the NG Geltrode (NE032) is used. The difference of this electrode is that it is based on sintered Ag/AgCl pellet of 4 mm diameter and has a contact area of about 1 cm2. Two electrodes of the type Sticktrode (NE025) are placed on the bare skin mastoid and work as an electrical reference of the EEG system. This electrode type is pre-gelled with an adhesive side with non-irritating gel and a 24 mm diameter. Figure 2 shows the different types of electrode that are used.

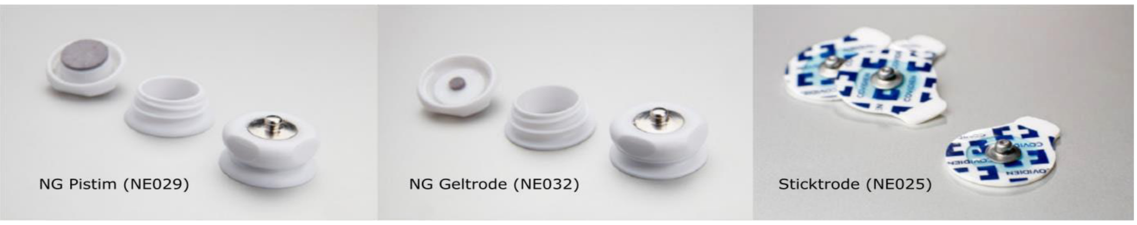

Figure 2. Electrode types of the Starstim system

4. It was added that the clinical trial was conducted according to the DIN EN ISO 14155.

Amendment 2 Protocol 1.2, June 2nd, 2020

1. The planned recruitment period was further extended to Q3/2021
2. Due to the appointment of Michael Siniatchkin in Bielefeld, the central server for all physiological data that were not included in the eCRF (EEG, structural and functional MRI, DTI) was provided by the Evangelical Hospital Bethel, Bielefeld through the Bechtle Hosting & Operations GmbH & Co. KGAG (previous server: electronic data processing center at the University of Kiel).

Amendment 3 Protocol 1.3, June 10th, 2021

1. Change of sponsor representative (dean) to Prof. Dr. rer. Nat. Daniela C. Dietrich, change of data management to Markus B. Schilhabel
2. The planned recruitment period was further extended to Q4/2021
3. Changes due to new legislatives, namely the Medical Device Implementation Act (MPDG, Medizinprodukterecht-Durchführungsgesetz), the Medical Device Regulation (EU) 2017/745 (MDR) and the Medical Device User Notification and Information Ordinance (MPAMIV, Medizinprodukte-Anwendermelde- und Informationsverordnung):
    - 8.3.2 Serious adverse events (p.38): As of 05/26/2021, the Clinical Trial will be considered as "Other Clinical Trials" according to the requirements of the Medical Device Regulation (MDR) 2017/745, amending ... (MDR= Medical Device Regulation) in Article 82 and the Medical Device Implementation Act (MPDG) Chapter 4, Subsection 2, §§ 47-70.

      Article 82 Requirements for other clinical trials are:

      (1) Clinical trials that are not conducted for one of the purposes referred to in Article 62(1) shall comply with the provisions of Article 62(2) and (3), (4)(b), (c), (d), (f), (h) and (1), and (6).

      In order to protect the rights, safety, dignity and well-being of subjects and to ensure compliance with scientific and ethical principles in clinical investigations which are not conducted for one of the purposes referred to in Article 62(1), each member state concerned shall lay down additional requirements appropriate for such investigations.

      According to MPDG Chapter 1, §2, No. 4, an "other clinical trial" of a device is a clinical trial that

        a. is not part of a systematic and planned process for product development or product

surveillance of a current or future manufacturer,

b. is not conducted with the aim of demonstrating the conformity of a device with the requirements of Regulation (EU) 2017/745,

c. serves to answer scientific or other questions; and

d. is conducted outside of a clinical development plan as defined in Annex XIV, Part A, point 1(a) of Regulation (EU) 2017/745.

Therefore, as of this date, SAE management will be conducted in accordance with the newly applicable requirements.

Any untoward medical occurrence in a clinical investigation experienced by the subject, the person applying the medical device or a third party after contact with the IMD, which does not necessarily have a causal relationship with this treatment, which directly or indirectly could have led or could lead to:

- death,
- a life-threatening situation,
- inpatient hospitalisation or prolongation of existing hospitalisation,
- persistent or significant disability/incapacity or a congenital anomaly/birth defect.

- <u>8.3.2.2 Reporting pathways, responsibility and deadlines (p.39):</u>

According to MPDG §63, the principal investigator or other investigators have the following duties. The investigator or principal investigator shall report the following to the sponsor of a clinical trial or other clinical investigation

1. without delay
   (a) any serious adverse event within the meaning of Article 2(58) of Regulation (EU) 2017/745; and
   (b) any device defect within the meaning of point 59 of Article 2 of Regulation (EU) 2017/745 that could have led to serious adverse events in the absence of appropriate measures or intervention or under less favorable circumstances,
2. according to the timing of the protocol, any type of adverse event as defined in Article 2(57) of Regulation (EU) 2017/745. Furthermore, § 66 "Self-responsible corrective measures" of the MPDG is now relevant.
   (1) lf circumstances arise during a clinical trial or other clinical investigations that may affect the safety of subjects, users or third parties, the sponsor and the investigator or principal investigator conducting the clinical trial or other clinical investigation shall immediately take all necessary safety measures to protect subjects, users or third parties from direct or indirect danger.
   (2) The sponsor shall immediately inform the competent higher federal authority, the regulatory authority responsible for him, the authorities responsible for the trial sites and the competent ethics committee of these new circumstances via the German Medical Devices Information and Database System (DMIDS) in accordance with Section 86 MPDG.

Each SAE related to the product or procedure and each product defect is analyzed and, if necessary, communicated to the BfArM as an individual case and/or as a collective notification (see quarterly safety report).

The sponsor maintains an SAE line listing and is responsible for further communication to the BOB in compliance with all legally required deadlines and channels as well as the specifications on the BfArM website incl. the use of the reporting forms available there.

- <u>8.4.2.1 Reporting pathways, responsibility and deadlines (p.40):</u>

The reporting period for SA-Es corresponds to the period between the first study-specific action (consent) and the four weeks follow-up period.

For patients who prematurely discontinue the clinical trial unscheduled, SADEs will be documented and reported until at least one week after treatment or until the principal investigator and sponsor agree that the event has been satisfactorily followed up, which includes:

- Symptoms have resolved and a plausible explanation for the SAE has emerged.
- The event is no longer considered serious.
- The subject's death has occurred.

In order to provide a detailed description of the corrective measures taken after the event, as weil as the assessment and justification of the causality for the examination with yes, possible or no

regarding:

- Medical procedure,
- Medical device,

an additional sponsor evaluation is to be provided by the sponsor.
The Sponsor Evaluation form and the SAE to be evaluated will be emailed for evaluation. Processing must be completed within 48 hours.
The sponsor evaluation will be faxed back to the KKS. The original will remain with the sponsor evaluator for now and will be collected by the monitor no later than Close out visit for filing in the TMF.

- 13.6 Responsibilities (p. 48):
  The Sponsor is responsible for the vigilance and control of the clinical trial, and will conduct the clinical study according to this protocol, all applicable national regulations, the principles of Good Clinical Practice according to the DIN EN ISO 14155, and the Medical Device Implementation Act (MPDG), the Medical Device Regulation (EU) 2017/745 (MDR) and the Medical Device User Notification and Information Ordinance (MPAMIV).

Statistical Analysis Plan

Improving neuropsychological functions and clinical course in children and adolescents with ADHD with anodal transcranial direct current stimulation (tDCS) of the prefrontal cortex: a randomized, double-blind, sham-controlled, parallel group trail using an uncertified class IIa device

E-StimADHD

Version Nr. 1.0, 01.07.2021

Sponsor

Dean: Prof. Dr. Daniela C. Dieterich

Otto-von-Guericke University Magdeburg

Medical Faculty

Leipziger Str. 44

39120 Magdeburg

Coordinating Investigator

Prof. Dr. Kerstin Krauel

Department of Child and Adolescent

Psychiatry and Psychotherapy

Otto-von-Guericke University Magdeburg

Medical Faculty

Leipziger Str. 44

39120 Magdeburg

Confidentiality

The information in this statistical analysis plan is strictly confidential. It is only to be viewed by the investigator, further individuals participating in the conduct of this study, ethics committees.

This statistical analysis plan must not be passed on to third parties without the sponsor's or the coordination investigator's permission.

Signatures

The persons listed here agree to the clinical study as outlined in this protocol by their signature and agree to conduct the clinical study according to this protocol, all applicable national regulations and the principles of Good Clinical Practice.

Sponsor's Representative
Martina Beckmann

Magdeburg, 06.07.21
Location, Date

Signature

Coordinating Investigator
Prof. Dr. Kerstin Krauel

Magdeburg, 08.07.21
Location, Date

Signature

Statistician
Prof. Dr. Astrid Dempfle

Kiel, 1.7.2021
Location, Date

Signature

Table of Contents

1. Introduction 4

2. Study objectives 4

2.1 Primary objective 4

2.2 Secondary objectives 5

3. Study design 5

4. Participating sites 5

5. Analysis population 5

5.1 Definitions of data sets 6

5.2 Protocol violations 7

6. Variables and study endpoints 8

6.1 Demographic and baseline characteristics 8

6.2 Primary endpoints 8

6.3 Secondary endpoint(s) 9

7. Statistical analyses 12

7.1 Outliers 12

7.2. Demographic and baseline characteristics 13

7.3. Primary endpoints 13

7.4. Secondary endpoints 14

7.5. Safety analyses 14

7.6. Missing values 15

7.7. Interim analysis 15

8. Software 15

9. References 16

10. Appendices 17

10.1 Appendix 1 17

10.2 Appendix 2 19

10.3 Appendix 3 20

10.4 Appendix 4 21

10.5 Appendix 5 22

1. Introduction

The EU research project "Stimulation in pediatrics" (STIPED) consists of ten different work packages (WP1 to WP10), working closely together and covering all important aspects concerning the investigation of transcranial direct current stimulation (tDCS) as a treatment method for patients with Attention Deficit Hyperactivity Disorder (ADHD) and Autism Spectrum Disorder (ASD).

This statistical analysis plan (SAP) provides detailed information about planned data analyses in **WP4 (E-StimADHD)**. It describes general statistical considerations and strategies that will be used to address specific objectives and aims, the statistical methods to be employed in specific types of analyses, and the statistical analyses to be conducted to assess each specific study outcome. The present SAP is based on the final study protocol of E-StimADHD (Version Nr. 1.3, 10.06.2021) and provides additional details beyond those specified in the protocol.

All statistical methods described in this SAP refer to neuropsychological performance measures (i.e. output of computerized neuropsychological tasks) and clinical variables only (i.e., documented in the eCRF). The IMIS is responsible for the analysis of these variables but not for other data (in particular raw EEG or MRI data). Moreover, other data from substudies (e.g., raw MRI or EEG data) are not covered by analyses described in this SAP. In particular, the IMIS will not perform additional analyses that are only relevant for doctoral theses. Hence, these additional analyses are not described in the current SAP.

2. Study objectives

The E-StimADHD is a phase-IIa, randomized, double-blind, sham-controlled, parallel group trial. The study investigates whether repeated multi-channel tDCS stimulation of the left dorsolateral prefrontal cortex (dlPFC, **Study A**) or the right inferior gyrus (rIFG, **Study B**) significantly improves relevant neuropsychological parameters in young patients aged 10 to 18 years with ADHD. In particular, it is assumed that tDCS stimulation improves interference control, response inhibition, and working memory short- and long-term after 4 weeks. Results of the E-StimADHD study will inform about potential effect sizes and the therapeutic potential of tDCS in ADHD. This general objective is divided into one primary and three secondary objectives as described in the following sections.

2.1 Primary objective

The primary objective of the E-StimADHD study is to investigate effect sizes of changes in neuropsychological performance, study safety and tolerability of multi-channel anodal tDCS (anodal tDCS vs. sham stimulation) targeting the specific brain region left dlPFC (**Study A**) or rIFG (**Study B**) in children and adolescents with ADHD.

2.2 Secondary objectives

The three secondary objectives of the E-StimADHD study are:

(1) to investigate the potential benefit of multi-channel anodal tDCS targeting dlPFC (**Study A**) or rIFG (**Study B**) on behavior and quality of life in children and adolescents with ADHD.
(2) to identify individual factors derived from MRI and/or EEG measures as well as clinical measures to predict responsivity to stimulation.
(3) to assess expectations and concerns of parents and patients towards tDCS.

3. Study design

Both **Study A** and **Study B** are randomized, double-blind, sham-controlled clinical trials with two parallel arms (1:1 randomization: anodal tDCS vs. sham stimulation) and four measurement points (T1: Screening, T2: Baseline, T3: Post, T4: Follow-up, see Figure 1). The intervention is a repeated anodal tDCS over the left dlPFC (**Study A**) or the rIFG (**Study B**) during an ongoing task related to neuropsychological performance (10 days of stimulation 20 min / day, with 1mA intensity). Blinded sham stimulation is applied in the same setting without an effective electrical current. The studies will be conducted in the laboratory setting.

4. Participating sites

Participating sites are (1) the Clinical and Academic Center (ICNAS), University of Coimbra, (2) the Department of Child and Adolescent Psychiatry, Psychosomatics, and Psychotherapy, Goethe-University Frankfurt am Main, (3) the Clinic of Child and Adolescent Psychiatry and Psychotherapy, Evangelisches Klinikum Bethel (EvKB), (4) the Department of Child and Adolescent Psychiatry, Center for Integrative Psychiatry Kiel, and (5) the Department of Child and Adolescent Psychiatry and Psychotherapy, Otto-von-Guericke-University Magdeburg. All sites are highly experienced in the use of various neuroscientific methods and have state-of-the-art EEG and MRI facilities at their disposal.

5. Analysis population

For both **Study A** and **Study B** three patient populations will be defined. The statistical analyses of the trial results will be performed on both the *intention-to-treat* (ITT) and on the *per-protocol* (PP) data set. The PP analysis will be performed as sensitivity analysis. Safety data will be summarized using the *safety analysis* (SA) data set.

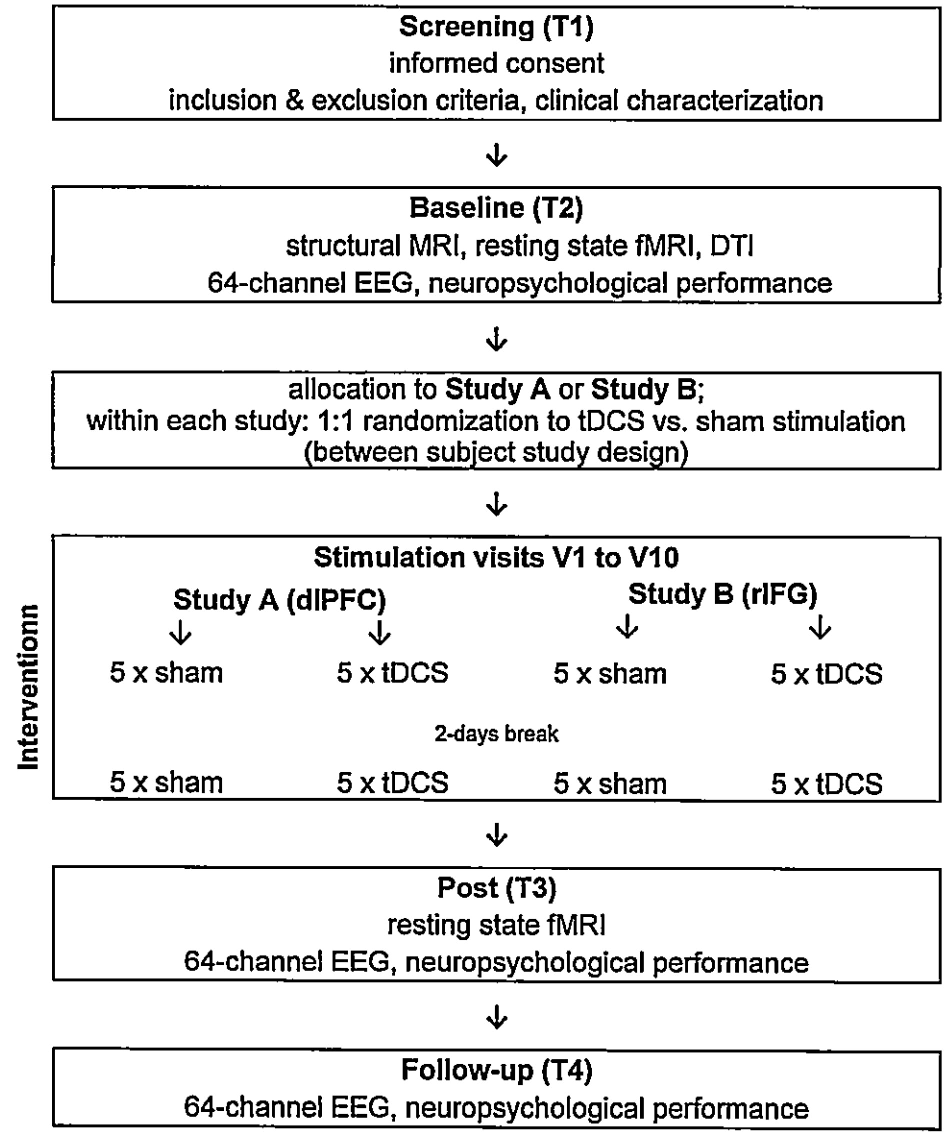

Figure 1: Flow-Chart of the E-StimADHD study. Each subject will attend four measurement points (T1: Screening, T2: Baseline, T3: Post, T4: Follow-up). Allocation to study arms (**Study A** or **Study B**) as well as electric brain stimulation (anodal tDCS vs. sham stimulation) happens between Baseline (T2) and Post (T3).

5.1 Definitions of data sets

(1) The **intention-to-treat (ITT)** data set includes all patients as originally randomized to anodal tDCS or sham stimulation (see Figure 1), irrespective of the total amount of stimulation actually received. Hence, in the ITT analysis a participant is analyzed in the stimulation condition allocated even if he or she received no stimulation at all, interrupted the stimulation, prematurely discontinued the stimulation, received the wrong stimulation condition or in any other way violated the study protocol (for detailed information about possible protocol violations see chapter 5.2). The primary analysis for the E-StimADHD study will be based on the ITT data set which will provide unbiased comparisons among anodal tDCS and sham stimulation.

(2) The participants who completed the study without any major protocol violations (see chapter 5.2) will be analyzed in the **per protocol (PP)** data set. A PP analysis includes only those participants who adhered to all important aspects of the study protocol and, thus, provides an estimate of the optimal efficacy of anodal tDCS in comparison to sham stimulation.

(3) The **safety (SA)** data set includes all randomized subjects who started at least 1 double-blind stimulation session (stimulation visits V1 to V10 between Baseline (T2) and Post (T3), see Figure 1). It will be the main data set for the analysis of safety and tolerability of anodal tDCS stimulation. Patients will be analyzed according to the actually received treatment.

5.2 Protocol violations

In general, a major protocol violation is defined as a relevant non-compliance to the study protocol that leads to exclusion of participants from the PP data set (Bhatt, 2012).

In both studies, E-StimADHD **Study A** and **Study B**, relevant protocol violations are:

(1) Protocol violations that lead to **exclusion** of the participant **from the per protocol analysis** (sham vs. verum at T3/V11):

- V0 or V11 is missing
- missing two or more of the ten stimulation sessions
- on two or more of the ten stimulation sessions, the total duration of stimulation was below 18 minutes for any reason including malfunction or incorrect use of the device
- the placement of tDCS electrodes was incorrect on two or more of the ten stimulation sessions
- stimulation medication at V0 and/or during stimulation (V1-V10) and/or at V11
- occurrence of a neurological or psychiatric disorder included in the exclusion criteria after screening
- start of participation in a clinical trial or neurofeedback after screening

(2) Further important protocol violations are **delayed visits** (e.g., due to sickness):

- time period between V1 and V10 exceeds 3 weeks
- time period between V10 and V11 is longer than 8 days

Participants with one or more protocol violations are excluded form PP analyses. The motivation is to ensure that comparisons are made between those participants only who adhered in important aspects to the study protocol.

The primary analysis will be based on the ITT set including all randomized participants. A PP analysis will be performed as sensitivity analysis. Occurring differences between both sets will be documented in the result report.

Further protocol violations regard only a subset of data:

Protocol violations relevant to the **analysis of V12:**

- V12 is missing
- the participant has been excluded from the analysis of V11

- period shorter than 4 weeks between V10 and V12
- Participant took **stimulant medication** between V11 and V12

Protocol violations relevant to the **analysis of questionnaire data:**

- questionnaires are returned more than 7 days after the respective visit

6. Variables and study endpoints

6.1 Demographic and baseline characteristics

For the full ITT data set and separately for the two study arms (**Study A**: left dlPFC; **Study B**: rIFG), demographic and baseline characteristics will be summarized using descriptive summary statistics. Absolute and relative frequencies will be reported for categorical and dichotomous variables. For metric variables the mean value, standard deviation, median, the interquartile range, minimum, and maximum will be calculated. Results will be presented using tables (see Table 1 in Appendix 1) and boxplots where appropriate.

Information about patients with ADHD and their parent(s) will be recorded at Visit 1.0 (T1 – Screening). Demographic data of ADHD patients will include the variables *gender*, *age*, *ethnicity*, *height*, *weight*, *Body Mass Index* (*BMI*), and *BMI percentiles* (based on KIGGS reference data) and other variables related to their sociodemographic background (see Table 1). In addition, inclusion and exclusion criteria will be saved and documented as dichotomous variables (1=fulfilled, 0=not fulfilled, see Table 2 in Appendix 2). Dichotomous variables of inclusion and exclusion criteria will be summarized in absolute and relative frequencies and will be visualized in a CONSORT-Flow diagram.

Furthermore, patients' characteristics before any stimulation session will be summarized with descriptive statistics. This will include the variables *handedness* (Edinburgh Handedness Inventory, child rating), *socioeconomic status of the parents* (e.g., net income of the household, school graduation level, and current professional situation of the parents), *pubertal status* (Pubertal Development Scale, child and parent rating), *expectations and concerns towards tDCS* (survey of child and parent), *severity of ADHD symptoms* (ADHD rating scale, child and parent rating), and *quality of life* (KIDSCREEN-27, child and parent rating).

6.2 Primary endpoints

To measure effect sizes of changes in neuropsychological performance, study safety, and tolerability of multi-channel anodal tDCS (anodal tDCS vs. sham stimulation) in children and adolescents with ADHD, primary endpoints of the EStimADHD study were adapted to the specific stimulated brain area (**Study A**: left dlPFC; **Study B**: rIFG) and are presented separately below.

Study A:

The **n-back working memory task** is a continuous performance task that is commonly used as an assessment in cognitive neuroscience to measure working memory capacity (Gazzaniga, Ivry, & Mangun, 2009). In the n-back working memory task, a series of pictures is presented, with 25% of the n-back trials being target trials, which means that the currently

presented stimulus is identical with the stimulus n trails earlier (Kirchner, 1958). ADHD patients will be instructed to decide by button press if the current stimulus is a target or not.

The n-back working memory task is used in **Study A** to assess patients' working memory capacity. Hence, the primary endpoint is the ***overall accuracy*** (i.e., sum of correct hits, correct rejections, correct rejections 1lure, and correct rejections 2lure in percent, variable *nback_oa*) in the **n-back working memory task** (Gazzaniga, Ivry, & Mangun, 2009) which assesses patients' working memory. The n-back task will be used throughout **Study A** and effectiveness of anodal tDCS stimulation will be evaluated by analyzing patients' performance at Visit V11 (T3: Post; see Figure 1), adjusted for performance at Visit V0 (T2: Baseline).

Study B:
In the **flanker task** (target task), stimuli consist of five arrows. The middle arrow is the target stimulus and participants should indicate via button press with the right or with the left hand if the target arrow points to the right or to the left. The outer arrows serve as distractors and can point in the same direction (congruent trial) or in the opposite direction (incongruent trial). The flanker task provides information on cognitive interference control.

The primary endpoint for Study B is the ***overall accuracy*** (i.e., correct hits for congruent and incongruent trails in percent, variable *flanker_oa*) in the flanker task. Effectiveness of anodal tDCS stimulation will be evaluated by analyzing patients' performance at Visit V11 (T3: Post; see Figure 1), adjusted for performance at Visit V0 (T2: Baseline).

For both **study A** and **study B**, safety and tolerability of tDCS assessed by (1) a standardized safety questionnaire as well as (2) EEG recordings before and after tDCS (EEG abnormalities such as slowing under the stimulating electrode or in remote brain areas, polarization asymmetries, spike-and-wave dischanges) and (3) number, type, and severity of adverse events (AEs, variables *ad*, *ad_type*, and *ad_sev*) and severe adverse events (SAEs, variables *sae*, *sae_type*, and *sae_sev*) are defined as primary endpoints.

Note: Statistical analyses for raw EEG recordings are not included in this SAP. No raw EEG data will be analyzed by the IMIS. However, defined aggregated EEG data (defined abnormalities coded e.g., in SPSS files) as relevant for safety will be analyzed.

As an overall safety analysis of all STIPED studies, the safety endpoints ((S)AEs and safety questionnaire) will also be analyzed for Study A and Study B together (and furthermore also together with the safety data of the other studies in WP3, WP4 and WP5, details of this analysis will be specified elsewhere) in order to get a global safety assessment of tDCS in children and adolescents.

6.3 Secondary endpoint(s)

In addition to the primary endpoints, specific measures of the neuropsychological tasks will be used as secondary endpoints, such as accuracy, commission and omission errors, reaction time, and reaction time variability as secondary endpoints. Each of these secondary endpoints will be measured before (V1) and after (V11/V12) verum or sham stimulation and

effectiveness will be assess by comparing the values after stimulation between study arms (tDCS vs sham) adjusted for baseline values. In addition, the measures of the primary outcome (overall accuracy in n-back or flanker task) at the follow-up visit (V12) will be secondary endpoints.

For both **Study A** and **Study B**, severity of **ADHD symptoms** (inattention, hyperactivity/impulsivity) and **quality of life** (KIDSCREEN-27) as assessed by questionnaire at V11 and V12 will be secondary endpoints.

Secondary endpoints at V11 and V12 for **Study A** in the **target task** (n-back working memory task) are:

- The ***reaction time*** (variable *nback_rt*) for the overall accuracy in the n-back working memory task.
- The ***variability of the reaction time*** for the overall accuracy (i.e., standard error, variable *nback_rtSD*) in the n-back working memory task.
- All ***omission errors*** (i.e., sum of all missed target trials, variable *nback_omiss*) in the n-back working memory task.
- All ***commission errors*** (i.e., sum of all false alarms, false alarms 1lure, and false alarms 2lure, variable *nback_comiss*) in the n-back working memory task.

Secondary endpoints at V11 and V12 for **Study A** in the **first non-target task** (flanker task) are:

- The ***overall accuracy*** (i.e., sum of correct responses for congruent and incongruent trails in percent, variable *flanker_oa*) in the flanker task.
- The ***reaction time*** (variable *flanker_rt*) for the overall accuracy in the flanker task.
- The ***variability of the reaction time*** for the overall accuracy (i.e., standard error, variable *flanker_rtSD*) in the flanker task.
- All ***omission errors*** (i.e., sum of all omission errors for congruent and incongruent trials, variable *flanker_omiss*) in the flanker task.
- All ***commission errors*** (i.e., sum of all commission errors for congruent and incongruent trials, variable *flanker_comiss*) in the flanker task.

The Continuous Performance Task (CPT) is the **second non-target task** in Study A. In the CPT, various upper case letters will be presented. One of the letters will be defined as a cue and one as a target stimulus. Whenever the cue is followed by the target stimulus, participants are instructed to press a button. However, participants should withhold their response if the cue is followed by another stimulus. Secondary endpoints at V11 and V12 for Study A in the CPT are:

- The ***overall accuracy*** (i.e., sum of correct neutral, correct cues, correct non-targets, and correct targets, variable *CPT_oa*) in the CPT.
- The ***reaction time*** (variable *CPT_rt*) for the overall accuracy in the CPT.
- The ***variability of the reaction time*** for the overall accuracy (i.e., standard error, variable *CPT_rtSD*) in the CPT.
- All ***omission errors*** (i.e., sum of all missed targets, variable *CPT_omiss*) in the CPT.
- All ***commission errors*** (i.e., sum of all false alarms, false alarms for cues, and false alarms for non-targets, variable *CPT_comiss*) in the CPT.

Secondary endpoints at V11 and V12 for **Study B** in the **target task** (flanker task) are:

- The ***reaction time*** (variable *flanker_rt*) for the overall accuracy in the flanker task.
- The ***variability of the reaction time*** for the overall accuracy (i.e., standard error, variable *flanker_rtSD*) in the flanker task.
- All ***omission errors*** (i.e., sum of all omission errors for congruent and incongruent trials, variable *flanker_omiss*) in the flanker task.
- All ***commission errors*** (i.e., sum of all commission errors for congruent and incongruent trials, variable *flanker_comiss*) in the flanker task.

Secondary endpoints at V11 and V12 for **Study B** in the **first non-target task** (n-back working memory task) are:

- The ***overall accuracy*** (i.e., sum of correct hits, correct rejections, correct rejections 1lure, and correct rejections 2lure in percent, variable *nback_oa*) in the n-back working memory task.
- The ***reaction time*** (variable *nback_rt*) for the overall accuracy in the n-back working memory task.
- The ***variability of the reaction time*** for the overall accuracy (i.e., standard error, variable *nback_rtSD*) in the n-back working memory task.
- All ***omission errors*** (i.e., sum of all missed target trials, variable *nback_omiss*) in the n-back working memory task.
- All ***commission errors*** (i.e., sum of all false alarms, false alarms 1lure, and false alarms 2lure, variable *nback_comiss*) in the n-back working memory task.

Secondary endpoints at V11 and V12 for **Study B** in the **second non-target task** (CPT) are:

- The ***overall accuracy*** (i.e., sum of correct neutral, correct cues, correct non-targets, and correct targets, variable *CPT_oa*) in the CPT.
- The ***reaction time*** (variable *CPT_rt*) for the overall accuracy in the CPT.
- The ***variability of the reaction time*** for the overall accuracy (i.e., standard error, variable *CPT_rtSD*) in the CPT.
- All ***omission errors*** (i.e., sum of all missed targets, variable *CPT_omiss*) in the CPT.
- All ***commission errors*** (i.e., sum of all false alarms, false alarms for cues, and false alarms for non-targets, variable *CPT_comiss*) in the CPT.

In addition to secondary endpoints resulting from target and non-target tasks, **readout measures** common for **both Study A and B** will be assessed as additional secondary endpoints.

- Functional brain related outcome measures based on EEG: (1) functional and effective connectivity in the resting state EEG; (2) event related potentials: amplitude, latency variability, neuronal sources of task relevant components, power changes before and after stimuli in the single-trial analysis.

- Structural and functional and structural brain related outcome measures based on MRI: (1) gray and white matter thickness based on voxel based morphometry, cortical thickness and folding, gyrification index; (2) structural connectivity based on parameters of functional anisotropy and fiber tractography (DTI measures); (3) resting state functional connectivity as well as Graph theoretical measures (connectivity density; local and global efficacy e.c.) based on BOLD fMRI.

  Note: The IMIS is not responsible for the above mentioned readout measures. Statistical analyses for these readout measures are not included in this SAP. No raw EEG or MRI data will be analyzed by the IMIS. However, aggregated EEG/MRI data (e.g., in SPSS files) may be analyzed in some cases.

7. Statistical analyses

Each of the two studies (**Study A**: left dlPFC; **Study B**: rIFG) will be analyzed separately. Before the statistical analyses of primary and secondary outcomes in **Study A** and **Study B**, empirical data will be checked for plausibility (e.g., detecting unrealistic values). This step also includes outlier detection.

7.1 Outliers

An *outlier* is an observation that lies an abnormal distance from other values in a random sample from a population. Any kind of outliers (i.e., mild or extreme) will be identified before primary analyses. Without breaking the blinding, we will calculate descriptive statistics for all primary and secondary endpoints. Outliers will be visualized with scatter plots and box plots. Without breaking the blind, any outliers (in both study arms) will be handled in the same way: for data that is available in other records (e.g. demographic or clinical data available in clinical records), a query will be sent to the center to verify and confirm or correct the outlier. For values only available as source data (e.g. neuropsychological performance measures), it will be determined together with the coordinating investigator whether outliers may represent extreme but true values (which will be analyzed as such), or are so implausible that they represent measurement error (which will be removed from the data set). In particular for the primary outcome measures only clear measurement errors will be removed.

The box plot is a useful graphical display for describing the behavior of the data in the middle as well as at the ends of the distributions. The box plot uses the median and the lower and upper quartiles (defined as the 25th and 75th percentiles). If the lower quartile is Q1 and the upper quartile is Q3, then the difference (Q3 - Q1) is called the interquartile range or IQ. A box plot is constructed by drawing a box between the upper and lower quartiles with a solid line drawn across the box to locate the median. The following quantities (called fences) are needed for identifying outliers in the tails of the distribution:

1. lower inner fence: Q1 - 1.5*IQ

2. upper inner fence: Q3 + 1.5*IQ
3. lower outer fence: Q1 - 3*IQ
4. upper outer fence: Q3 + 3*IQ

A point beyond an inner fence on either side is considered a mild outlier. A point beyond an outer fence is considered an extreme outlier.

As an example, a SPSS syntax code for outlier detection is shown in Appendix 3. Here, outlier detection is conducted for the primary outcome in **Study A** (overall accuracy in the n-back working memory task, variable *nback_oa*).

7.2. Demographic and baseline characteristics

Demographic and baseline characteristics recorded at Visit 1.0 (before any stimulation, see Figure 1) will be analyzed with descriptive statistics. Sample characteristics will be reported for the full study sample as well as for each study arm separately.

For the categorical variables *gender, ethnicity, substance consumption, socioeconomic status, handedness, pubertal development, and pregnancy* we will report absolute and relative frequencies in tables and figures (see Table 1 in Appendix 1). For metric variables with a continuous scale (*age, height, weight, psychiatric assessment, social responsiveness, intelligence, arousal, and motivation)* we will report means, medians, standard deviations, and interquartile ranges. We will visualize empirical results with box plots and scatter plots, where appropriate.

7.3. Primary endpoints

To test potential effects of tDCS (anodal tDCS vs. sham stimulation) on working memory (**Study A**), or response inhibition (**Study B**), statistical analyses will be performed separately for each study. Primary endpoints will be the overall accuracy in the n-back working memory task (**Study A**), the overall accuracy in the flanker task (**Study B**).

For both study arms, the primary outcome of interest will be measured at the end of the intervention at T3 (Visit 11, variables with suffix _T3). Regarding these outcome variables, possible differences between anodal tDCS and sham stimulation will be tested using an analysis of covariance. In general, this is a regression model with one dependent outcome variable and several independent variables. Analyses will be performed separately for **Study A** and **Study B.** For **Study A**, the outcome variable in this model will be the overall accuracy in the n-back working memory task (*nback_oa_T3*). For **Study B**, it will be the overall accuracy in the Flanker task (*flanker_oa_T3*). In both models, we use the binary variable *stimulation* (coded as 1=anodal tDCS stimulation and 0=sham stimulation) as independent variable. Additionally, we will enter the (dummy-coded) variables *center, sex (1=female, 0=male), puberty status, age, IQ*, and the *baseline value* of accuracy in the respective tasks at T2 (Visit 0, variables with suffix _T2) as covariates into this model. Hence, we control for their possible effects on the primary outcome variables and calculate adjusted regression coefficients. In this study, randomisation was stratified by center, thus it is necessary to account for the center-effects in the analysis as well. This is because stratified randomisation leads to correlation between treatment arms, making it necessary to adjust for the

stratification factors in the analysis to obtain correct confidence intervals and p-values, maintain the type I error rate at its nominal level (usually set at 5%), and avoid a reduction in power (Kahan & Morris 2013 and ICH E9 guideline). Therefore *center* will be used as a fixed effect in the analysis. Similarly, since the baseline value is strongly related to outcome for the neuropsychological measures used here, this *baseline value* will be used as a covariate in the analysis. Further, it seems plausible that *sex (1=female, 0=male)*, *puberty status*, *age* and *IQ* are related to neuropsychological performance, however this effect might already be sufficiently captured by the baseline values of performance and additionally, *puberty status* and *age* can be expected to be correlated. Thus the effects of these covariates will first be investigated in a blinded manner in a model without *stimulation* before a decision is made whether to include them in the final model.

As an example, a SPSS syntax code for the planned statistical analyses is shown in Appendix 4. Here, the regression analysis is conducted for the primary outcome in **Study A** (overall accuracy in the n-back working memory task, variable *nback_oa_T3*).

Results (e.g., standardized and unstandardized regression coefficients, standard errors, confidence intervals, and p-values) will be presented and summarized in tables and figures (for an example see Table 3 in Appendix 5). If the regression coefficient for *stimulation* is positive and has a p-value <0.05, superiority of tDCS over sham will be concluded. As this is a phase II trial, emphasis will be on effect size estimates and corresponding 95% confidence intervals. Empirical results based on the ITT set will be assured with sensitivity analyses (i.e., validation in the PP set).

7.4. Secondary endpoints

Statistical analyses for all secondary outcome variables listed in chapter 6.3 including quality of life and the additional outcomes at the follow-up assessment (T4, Visit 12) will be analogous to the primary outcome analyses. We will use the same regression model as described in chapter 7.3 but will replace the dependent variable in the SPSS syntax code (see Appendix 4) with all secondary outcomes, successively and include the baseline value of the secondary outcome as a covariate. Hence, we calculate for each secondary outcome variable a separate regression model. Presentation of model results will be identical to primary outcome analyses (see Appendix 5).

Note, however, that all analyses of functional brain related outcome measures and all outcomes from sub-studies are the responsibility of the respective investigators. The statisticians of the IMIS will not perform any statistical analyses located in this field.

7.5. Safety analyses

For safety evaluations, we will use descriptive statistics for each study (**Study A** and **Study B**) separately and combined. We will tabulate absolute and relative frequencies of adverse events (AEs) and severe adverse events (SAEs) by study arm (tDCS and sham), severity and investigator-assessed relation to investigational treatment. No statistical tests will be performed for (S)AEs which are expected to be rare. In addition, safety and tolerability of tDCS stimulation will be evaluated with the standardized safety questionnaire and differences between study arms (tDCS and sham) will be analyzed in an analogous way to endpoint analysis. We will visualize empirical findings with bar charts and box plots.

7.6. Missing values

In the primary analysis of the primary endpoint, we will use multiple imputations to account for missing data. Multiple imputations are currently the preferred method for dealing with missing values (Graham et al., 2003) under the assumption of missing at random (MAR).

Sensitivity analyses will be performed to investigate the potential impact of missing data, in particular, by using data of both the end-of-treatment (V11) and follow-up (V12) assessments in a mixed-effects model repeated measures analysis to estimate the treatment contrast at V11, a complete case analysis and specification of a range of plausible values for missing data (worst-best case scenarios under a missing-not-at-random (MNAR) assumption) to evaluate the potential departure of statistical estimates and inference from those obtained in the primary analysis under an MAR assumption.

7.7. Interim analysis

There will be no interim analysis.

8. Software

Descriptive as well as inferential statistics will be calculated in *IBM SPSS Statistics* for Windows (Version 25). For transparency reasons, all SPSS data and syntax files will be saved electronically. Comments in SPSS syntax files will guide the reader through every step of the analyses.

9. References

Bhatt A. (2012). Protocol deviation and violation. *Perspectives in clinical research*, *3*(3), 117. doi:10.4103/2229-3485.100663

Graham J.W., Cumsille P.E., Elek-Fisk E. (2003). Methods for handling missing data. In: Research Methods in Psychology (Schinka J.A., Velicer W.F., eds), pp 87-114. New York: John Wiley & Sons.

IBM (2017). IBM SPSS Statistics (version 25 for Windows) [Computer software]. Armonk, NY: IBM.

Kahan BC, Morris TP. Analysis of multicentre trials with continuous outcomes: when and how should we account for centre effects? Stat Med. 2013;32(7):1136–1149. doi: 10.1002/sim.5667.

International Council for Harmonisation of Technical Requirements for Registration of Pharmaceuticals for Human Use (ICH) guideline E9: statistical principles for clinical trials (1998)

10. Appendices

10.1 Appendix 1

Table 1: Participants' Baseline Characteristics at Visit 1.0 (T1 - Screening)

Variables	full sample (n=xx)	Study A (n=xx)	Study B (n=xx)
Gender (female), n (%)			
Ethnicity			
White/Caucasian, n (%)			
Oriental/North African/Latin America, n (%)			
Black African, n (%)			
Asian, n (%)			
Other, n (%)			
Age (years), mean (SD), Min, Max			
Height (cm), mean (SD), Min, Max			
Weight (kg), mean (SD), Min, Max			
Body Mass Index (BMI), mean (SD), Min, Max			
Body Mass Index (BMI), percentile, mean (SD), Min, Max			
Substance consumption (yes), n (%)			
Socioeconomic status			
1, n (%)			
2, n (%)			
3, n (%)			
4, n (%)			
5, n (%)			
Social responsivesness, mean (SD), Min, Max			
Handedness			
total left side, n (%)			
total right side, n (%)			
retrained, n (%)			
Intelligence (IQ), mean (SD), Min, Max			
Pubertal development			
Body hair growth			
has not yet begun to grow, n (%)			
has barely started to grow, n (%)			
is definitely underway, n (%)			
seems completed, n (%)			
Breasts/Facial hair			
has not yet begun to grow, n (%)			
has barely started to grow, n (%)			
is definitely underway, n (%)			
seems completed, n (%)			
Girls: Menstruation (yes), n (%)			
Boys: Change of voice (yes), n (%)			
Assessment of arousal/motivation (before stimulation)			
Did something exciting happen (yes), n (%)			
Caffeine consumption (yes), n (%)			
Smoking last 48 hours (yes), n (%)			
Smoking last 4 hours (yes), n (%)			
Alcohol last 24 hours (yes), n (%)			
Amount of sleep last night [hh], mean (SD), Min, Max			

SAM Item Mood, mean (SD), Min, Max			
SAM Item Arousal, mean (SD), Min, Max			
SAM Item Dominance, mean (SD), Min, Max			
Tiredness, mean (SD), Min, Max			
Motivation, mean (SD), Min, Max			
Concentration, mean (SD), Min, Max			
Time to last menstruation [days], mean (SD), Min, Max			

Note: This table contains information about participants' baseline characteristics at Visit 1.0 (T1 – Screening) for the full ITT data set as well as for each of the two study arms (**Study A**: left dlPFC; **Study B**: rIFG).

10.2 Appendix 2

Table 2: Inclusion and exclusion criteria in the E-StimADHD study and corresponding variable names

Inclusion criteria	Variable name
- ADHD according to DSM-5 (all subtypes)	*inclusion_1*
- age 10 to 18 years	*inclusion_2*
- the participant and their custodians are able and willing to give written informed assent/consent	*inclusion_3*
**Exclusion criteria**	**Variable name**
- IQ < 80	*exclusion_1*
- birth weight < 2500 gr.	*exclusion_2*
- born before the 37th week of pregnancy	*exclusion_3*
- past or present neurological diseases/brain surgery	*exclusion_4*
- dermatological diseases of the scalp	*exclusion_5*
- all comorbid psychiatric disorders other than oppositional defiant disorder, conduct disorder, elimination disorders, anxiety disorders, learning disorders	*exclusion_6*
- history of craniocerebral injury with loss of consciousness	*exclusion_7*
- heart disease	*exclusion_8*
- pregnancy	*exclusion_9*
- concurrent neurofeedback therapy	*exclusion_10*
- concurrent pharmacological treatment of ADHD	*exclusion_11*

Note: Variables listed in the second column of this table will be dichotomous (1=fulfilled, 0=not fulfilled).

10.3 Appendix 3

```
DESCRIPTIVES VARIABLES= nback_oa
 /STATISTICS=MEAN STDDEV MIN MAX.

FREQUENCIES VARIABLES= nback_oa
 /NTILES=4
 /STATISTICS=STDDEV VARIANCE RANGE MINIMUM MAXIMUM MEAN MEDIAN
 /ORDER=ANALYSIS.

EXAMINE VARIABLES= nback_oa
 /PLOT NONE
 /PERCENTILES(5,10,25,50,75,90,95) HAVERAGE
 /STATISTICS DESCRIPTIVES EXTREME
 /CINTERVAL 95
 /MISSING LISTWISE
 /NOTOTAL.

EXAMINE VARIABLES= nback_oa
 /PLOT=BOXPLOT
 /STATISTICS=NONE
 /NOTOTAL
 /ID=vp_nr.
```

Note: This is an example for outlier detection conducted in the statistical software SPSS for Windows. The SPSS syntax code checks the primary outcome in **Study A**, the overall accuracy in the n-back working memory task (variable *nback_oa*), with descriptive statistics and boxplots for plausibility reasons.

10.4 Appendix 4

```
REGRESSION
 /DESCRIPTIVES MEAN STDDEV CORR SIG N
 /MISSING LISTWISE
 /STATISTICS COEFF OUTS CI(95) R ANOVA
 /CRITERIA=PIN(.05) POUT(.10)
 /NOORIGIN
 /DEPENDENT nback_oa_T3
 /METHOD=ENTER
stimulation
center
sex
*puberty status
age
IQ
nback_oa_T2
.
```

Note: This is an example for the primary outcome analyses in the statistical software SPSS for Windows. The SPSS syntax code shows a regression analysis with the primary outcome in **Study A** (overall accuracy in the n-back working memory task, variable *nback_oa_T3*) as dependent variable. The variables *stimulation* (coded as 1=anodal tDCS stimulation and 0=sham stimulation), *center*, *sex*, *puberty status (only to be included if correlation with age is not too large)*, *age*, *IQ*, and the baseline value of accuracy in the respective tasks at T2 (Visit 0, *nback_oa_T2*) enter the regression model as independent variables.

10.5 Appendix 5

Table 3 below illustrates how empirical results of primary outcome analyses will be summarized. Here, some fake results of **Study A** are presented. Model results of **Study B** will be presented in a similar way.

Table 3

Model results for ***Study A***

Model	IV	*b*	S.E.	CI [*b*]	*β*	*p*
**Study A**	stimulation	xxx	xx	xxx	xxx	xxx
	center A	xxx	xx	xxx	xxx	xxx
	sex	xxx	xx	xxx	xxx	xxx
	*puberty status	xxx	xx	xxx	xxx	xxx
	age	xxx	xx	xxx	xxx	xxx
	IQ	xxx	xx	xxx	xxx	xxx
	overall accuracy n-back at T2	xxx	xx	xxx	xxx	xxx

Notes. N = [*insert sample size*]; stimulation (1=anodal tDCS stimulation, 0=sham stimulation), center A (1=yes, 0=no), sex (1=female, 0=male), IV = independent variable; b = unstandardized regression weight; S.E. = standard error; CI [b] = confidence interval for unstandardized regression weight; *β* = standardized regression weight; p=p-value.
