# Supplementary material for: Prefrontal Transcranial Direct Current Stimulation in Pediatric Attention-Deficit/Hyperactivity Disorder: A Randomized Clinical Trial
Source: JAMA Netw Open. 2025 Feb 21;8(2):e2460477. doi: 10.1001/jamanetworkopen.2024.60477 (PMC11846015; doi:10.1001/jamanetworkopen.2024.60477)
Supplement: Supplement 2. — eTable 1. Ethical votes eTable 2. Additional sample characteristics eTable 3. Results of health related quality of life eTable 4. Safety and tolerability eTable 5. Unpleasant sensations during stimulation eFigure 1. Procedure eFigure 2. Raw means for working memory performance (d-prime) and flanker effect accuracy eMethods 1. Participants eMethods 2. Sample size eMethods 3. Optimized montages eMethods 4. Cognitive tasks eMethods 5. Randomization/sequence generation/allocation concealment eMethods 6. Blinding eMethods 7. Statistical methods eResults. Recruitment [file jamanetwopen-e2460477-s002.pdf]

Supplementary Online Content

Krauel K, Brauer H, Breitling-Ziegler C, et al. Prefrontal transcranial direct current stimulation in pediatric ADHD: a randomized clinical trial. *JAMA Netw Open*. 2025;8(2):e2460477. doi:10.1001/jamanetworkopen.2024.60477

eTable 1. Ethical votes
eTable 2. Additional sample characteristics
eTable 3. Results of health related quality of life
eTable 4. Safety and tolerability
eTable 5. Unpleasant sensations during stimulation
eFigure 1. Procedure
eFigure 2. Raw means for working memory performance (d-prime) and flanker effect accuracy
eMethods 1. Participants
eMethods 2. Sample size
eMethods 3. Optimized montages
eMethods 4. Cognitive tasks
eMethods 5. Randomization / sequence generation / allocation concealment
eMethods 6. Blinding
eMethods 7. Statistical methods
eResults. Recruitment
eReferences.

This supplemental material has been provided by the authors to give readers additional information about their work.

© 2025 Krauel K et al. *JAMA Network Open*.

eTable 1. Ethical votes

Involved ethics committees	Reference
Ethik-Kommission der Otto-von-Guericke-Universität an der Medizinischen Fakultät und am Universitätsklinikum Magdeburg, Germany	177/17
Ethik-Kommission der Medizinischen Fakultät der Christian-Albrechts-Universität zu Kiel, Germany	B299/17
Ethik-Kommission des Fachbereichs Medizin der Goethes-Universität Frankfurt am Main, Germany	17/18B
Comissão de Ética para a Investigação Clinica (CEIC), Portugal	1811HB959e
Ethikkommission der Ärztekammer Westfalen-Lippe und der Westfälischen Wilhelms-Universität Münster, Germany	2019-264-b-M

© 2025 Krauel K et al. *JAMA Network Open*.

eTable 2. Additional sample characteristics

	Study A (N=35)		Study B (N=34)	
	Verum (N=16)	Sham (N=19)	Verum (N=17)	Sham (N=17)
**Smoker**	0 (0%)	0 (0%)	1 (6%)	1 (6%)
**Puberty status (N%)**				
pre-pubescent	5 (31%)	3 (16%)	3 (18%)	1 (6%)
beginning puberty	3 (19%)	5 (26%)	1 (6%)	2 (12%)
in the middle of puberty	4 (25%)	7 (37%)	8 (47%)	8 (47%)
advanced puberty	4 (25%)	3 (16%)	5 (29%)	5 (29%)
post-pubescent	0 (0%)	1 (5%)	0 (0%)	1 (6%)
**Ethnicity (N%)**				
White/Caucasian	16 (100%)	16 (84%)	17 (100%)	17 (100%)
Oriental/NorthAfrican/ Latin American	0 (0%)	2 (11%)	0 (0%)	0 (0%)
African/Asian	0 (0%)	1 (5%)	0 (0%)	0 (0%)
**Socioeconomic status (N%)**				
1 (low)	2 (13%)	3 (16%)	4 (24%)	2 (12%)
2 (lower medium)	7 (44%)	5 (26%)	3 (18%)	2 (12%)
3 (medium)	3 (19%)	4 (21%)	0 (0%)	3 (18%)
4 (upper medium)	1 (6%)	3 (16%)	3 (18%)	2 (12%)
5 (high)	3 (19%)	4 (21%)	6 (35%)	7 (41%)
unknown	0 (0%)	0 (0%)	1 (6%)	1 (6%)
**Handedness (N%)**				
left	4 (25%)	4 (21%)	0 (0%)	4 (24%)
right	11 (69%)	15 (79%)	17 (100%)	13 (76%)
unknown	1 (6%)	0 (0%)	0 (0%)	0 (0%)
**D-prime**				
Primary endpoint in study A, secondary endpoint in study B	0.95 (0.89)	0.92 (0.72)	1.24 (0.68)	1.14 (1.02)
**Flanker effect accuracy**				
Primary endpoint in study B, secondary endpoint in study A	-6.41 (9.05)	-18.39 (14.32)	-18.36 (14.35)	-9.81 (7.56)
**Secondary Endpoints M (SD)**				
**N-back**				
Hit rate %	64.1 (23.23)	55.52 (24.57)	56.28 (18.7)	60.31 (18.74)
RT hits	616.4 (275.8)	589.9 (188.5)	602.4 (179.3)	633.4 (262.3)
SD-RT targets	250.9 (159.3)	217.3 (91.7)	269.7 (154)	274.8 (100.8)
**Flanker**				
Commission errors incongruent %	29.49 (12.64)	36.95 (13.4)	30.98 (15.34)	18.25 (12)
RT incongruent	699.8 (304.4)	658.7 (159)	631.5 (239.9)	583.4 (84.33)
SD-RT incongruent	674 (1301.3)	381.9 (228.8)	274.6 (172)	238.1 (133.9)
**ADHD Ratingscale**				
Inattention	17.33 (6.61)	19.32 (3.48)	17.71 (5.6)	17.71 (6.03)
Hyperactivity	6.94 (5.2)	7.58 (3.75)	5.65 (3.72)	6.12 (4.22)
Impulsivity	5.56 (3.61)	6.68 (3.65)	5.76 (3.58)	7.18 (3.1)
Total	29.83 (13.03)	33.58 (8.97)	29.12 (10.27)	31 (11.69)
**Continuous performance task (CPT)**				
Accuracy targets	85.35 (22.84)	90.75 (13.51)	95.17 (8.01)	95.62 (6.1)
False alarms %	9.06 (16.48)	3.85 (6.88)	1.32 (2.86)	1.16 (1.72)

© 2025 Krauel K et al. *JAMA Network Open*.

	Study A (N=35)		Study B (N=34)	
	Verum (N=16)	Sham (N=19)	Verum (N=17)	Sham (N=17)
RT targets	440.9 (115.6)	443.5 (108.9)	430.8 (100.6)	502.7 (129.3)
SD-RT targets	154.7 (65.2)	152.6 (65.9)	133.2 (56.7)	158.6 (65.6)
**Kidscreen-27 Self-Rating**				
Physical Well-Being	48.9 (9.16)	46.98 (5.15)	48.21 (5)	49.86 (8.64)
Psychological Well-Being	46.69 (9.17)	48.09 (8.46)	47.88 (6.75)	50.62 (10.21)
Autonomy & Parents	48.12 (8.55)	49.07 (7.99)	46.93 (6.42)	53.9 (8.91)
Social Support & Peers	46.95 (12.66)	51.14 (7.54)	41.01 (15)	51.26 (9.83)
School Environment	48.1 (7.6)	45.68 (7.58)	49.88 (13.37)	43.51 (7.37)
**Kidscreen-27 Parent-Rating**				
Physical Well-Being	44.05 (6.28)	46.09 (8.64)	40.79 (8.49)	46.08 (7.99)
Psychological Well-Being	43.95 (7.64)	43.55 (7.77)	41.8 (11.49)	44.36 (9)
Autonomy & Parents	48.99 (8.87)	51.23 (8.88)	47.22 (5.95)	49.06 (6.4)
Social Support & Peers	43.08 (10.68)	49.6 (9.03)	44.99 (9.69)	48.52 (12.03)
School Environment	41.75 (8.25)	44.2 (9.01)	40.98 (10.72)	40.17 (7.31)

Baseline characteristics are reported for the verum and sham group in each study arm. Nominal variables are reported as numbers and percentages (n (%)).

© 2025 Krauel K et al. *JAMA Network Open*.

eTable 3. Results of health related quality of life

	Screening	Follow-up					
	Verum & Sham	Verum	Sham	Effects			
	mean (SE)	emmean (SE)	emmean (SE)	Effect size	Lower CL	Upper CL	*p* value
**Study A**							
**Kidscreen-27 Self-rating**							
Physical Well-Being	47.86 (1.22)	47.54 (2.27)	48.77 (2.19)	-0.15	-0.88	0.57	0.668
Psychological Well-Being	47.45 (1.47)	48.98 (2.35)	49.07 (2.22)	-0.01	-0.73	0.71	0.976
Autonomy & Parents	48.64 (1.38)	51.76 (2.00)	54.62 (1.85)	-0.41	-1.15	0.32	0.254
Social Support & Peers	49.22 (1.73)	46.07 (4.03)	49.55 (3.79)	-0.25	-0.99	0.49	0.496
School Environment	46.75 (1.28)	44.40 (3.01)	45.63 (2.62)	-0.14	-0.95	0.67	0.726
**Kidscreen-27 Parent-Rating**							
Physical Well-Being	45.16 (1.29)	43.63 (2.09)	44.58 (1.93)	-0.14	-0.92	0.65	0.722
Psychological Well-Being	43.73 (1.29)	42.90 (2.30)	47.59 (2.12)	-0.60	-1.37	0.17	0.114
Autonomy & Parents	50.21 (1.49)	49.11 (1.52)	49.22 (1.39)	-0.02	-0.80	0.75	0.955
Social Support & Peers	46.54 (1.73)	44.07 (2.89)	46.69 (2.61)	-0.30	-1.22	0.63	0.509
School Environment	43.09 (1.46)	43.35 (2.88)	45.18 (2.87)	-0.21	-1.10	0.67	0.621
**Study B**							
**Kidscreen-27 Self-rating**							
Physical Well-Being	49.01 (1.19)	46.41 (2.77)	48.58 (2.62)	-0.27	-1.13	0.58	0.513
Psychological Well-Being	49.21 (1.47)	49.96 (2.61)	47.84 (2.55)	0.28	-0.57	1.12	0.506
Autonomy & Parents	50.42 (1.45)	50.37 (2.52)	53.79 (2.27)	-0.49	-1.37	0.39	0.257
Social Support & Peers	46.14 (2.32)	48.49 (2.81)	48.23 (2.91)	0.03	-0.86	0.93	0.944
School Environment	46.60 (1.89)	48.06 (2.66)	47.81 (2.67)	0.04	-1.07	1.14	0.943
**Kidscreen-27 Parent-Rating**							
Physical Well-Being	43.43 (1.47)	46.36 (2.40)	44.00 (2.49)	0.34	-0.59	1.28	0.452
Psychological Well-Being	43.08 (1.76)	46.03 (3.29)	45.73 (3.23)	0.04	-0.82	0.89	0.930
Autonomy & Parents	48.17 (1.06)	49.68 (2.16)	48.01 (2.11)	0.27	-0.59	1.13	0.521
Social Support & Peers	46.81 (1.88)	52.25 (3.54)	51.88 (3.29)	0.04	-0.83	0.91	0.925
School Environment	40.56 (1.54)	44.68 (3.46)	44.64 (3.45)	0.00	-1.04	1.05	0.993

© 2025 Krauel K et al. *JAMA Network Open*.

eTable 4. Safety and tolerability

	Severity	sham	verum
AE by Severity	mild	55	51
	moderate	31	22
	severe	1	1
	**Relationship to intervention**	**sham**	**verum**
AE by likelihood of a relationship between AE and intervention	likely	7	3
	reasonable possibility	20	19
	no resonable possibility	61	48
	not assessable	6	2
	**System Organ Class**	**sham**	**verum**
AE by System Organ Class	Cardiac disorders	1	0
	Ear and labyrinth disorders	3	3
	Eye disorders	0	3
	Gastrointestinal disorders	14	16
	Immune system disorders	1	0
	Infections and infestations	15	7
	Injury, poisoning and procedural complications	16	13
	Musculoskeletal and connective tissue disorders	3	1
	Nervous system disorders	31	23
	Psychiatric disorders	3	3
	Reproductive system and breast disorders	1	0
	Respiratory, thoracic and mediastinal disorders	7	7
	**AE**	**sham**	**verum**
Detailed list of Adverse Events	Abdominal discomfort	2	0
	Abdominal pain	2	0
	Abdominal pain upper	3	6
	Accident	1	0
	Aggression	0	1
Detailed list of Adverse Events (continued)	Anxiety	1	0
	Back pain	2	1
	Buttock injury	0	1
	Catarrh	1	2
	Constipation	0	1
	Contusion	0	2
	Cough	1	1
	Diarrhoea	1	0
	Dizziness	0	2
	Dysmenorrhoea	1	0
	Ear pain	2	0
	Feeling of electric discharge	5	3
	Emetophobia	0	1
	Epistaxis	1	0
	Eye pain	0	1
	Fall	0	2
	Foot fracture	1	0
	Gastroenteritis	0	1
	Gastrointestinal infection	2	1
	Gastrooesophageal reflux disease	1	0
	Headache	30	20
	Herpes zoster	1	0
	Hypoacusis	0	1
	Influenza	1	0
	Injury	1	0
	Insomnia	1	0
	Joint injury	2	1
	Knee injury	0	1
	Limb injury	5	3
	Musculoskeletal stiffness	1	0
	Nasal congestion	2	1
	Nasopharyngitis	11	5
	Nausea	1	8
	Oropharyngeal pain	2	3
	Palpitations	1	0
	Paraesthesia	1	1
	Photopsia	0	1
	Seasonal allergy	1	0
	Sleep disorder	1	1
	Sunburn	1	0
	Thermal burn	0	1
	Tinnitus	0	1
	Vertigo	1	1
	Vomiting	4	1

© 2025 Krauel K et al. *JAMA Network Open*.

eTable 5 . Unpleasant sensations during stimulation

**Sensation**	**estimated marginal mean (95% CI)**		**p-value**
	verum	sham	
**Itching**	0.87 (0.58, 1.16)	1.04 (0.77, 1.31)	0.33
**Pain**	0.31 (0.07, 0.54)	0.43 (0.21, 0.64)	0.1
**Burning**	0.14 (0.01, 0.28)	0.20 (0.07, 0.33)	0.49
**Warmth**	0.22 (0.09, 0.35)	0.19 (0.07, 0.31)	0.70
**Metal taste**	0.026 (0.00, 0.05)	0.015 (0.01, 0.04)	0.41
**Fatigue**	0.56 (0.30, 0.82)	0.85 (0.61, 1.09)	0.07

Unpleasant sensations during stimulation (combined from Study A and B), estimated marginal means from a linear mixed model across all stimulation sessions; sensations were reported on an ordinal scale from 0=none to 3= strong.

© 2025 Krauel K et al. *JAMA Network Open*.

eFigure 1. Procedure

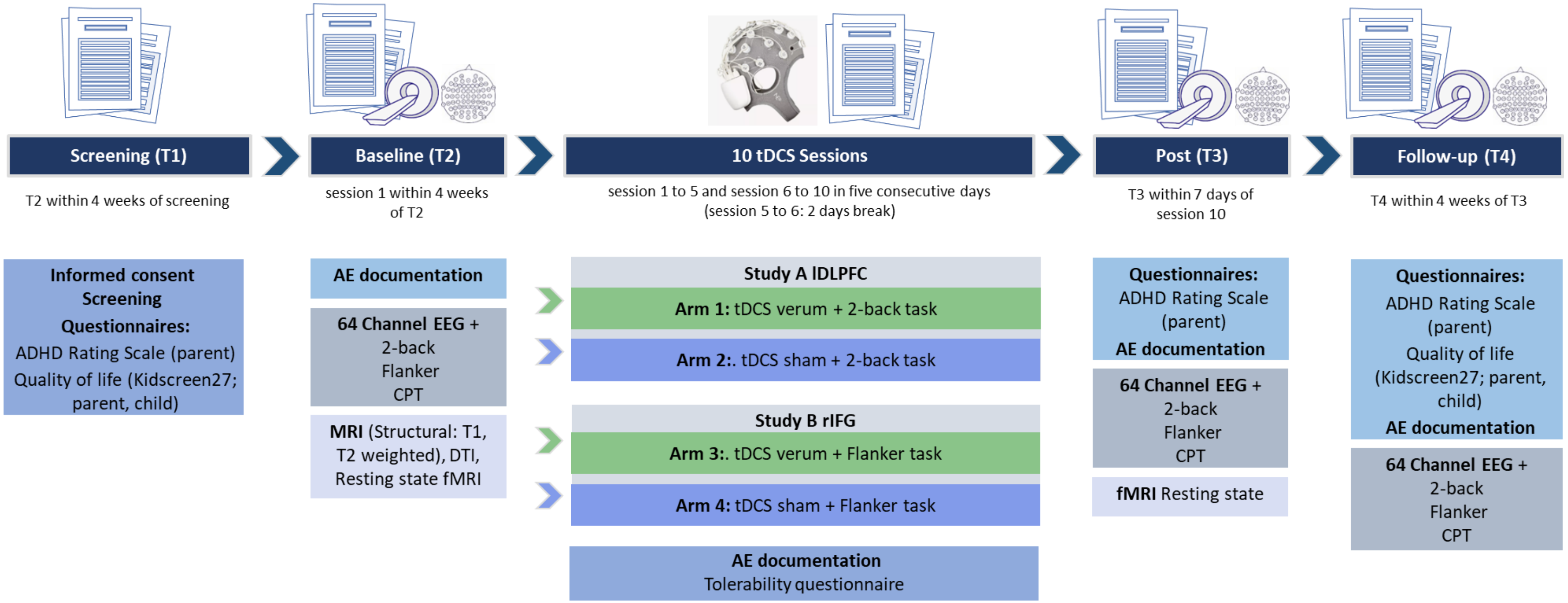

Verum stimulation is marked in green and sham stimulation in violet. Questionnaires are framed in blue, neurophysiological assessments in grey. Additional questionnaires that were assessed but are not reported in the current paper were a diary and a questionnaire about expectations towards tDCS stimulation. The diary was a questionnaire about arousal, mood and motivation of the patient on the day of the visit. It was assessed at the beginning and the end of each baseline, post and follow-up visit as well as every tDCS session. It also included questions about medication, caffeine, and nicotine intake. The questionnaire about expectations towards tDCS stimulation was completed by patients and parents at the screening and follow-up visit.

© 2025 Krauel K et al. *JAMA Network Open*.

eFigure 2. Raw means for working memory performance (d-prime) and flanker effect accuracy

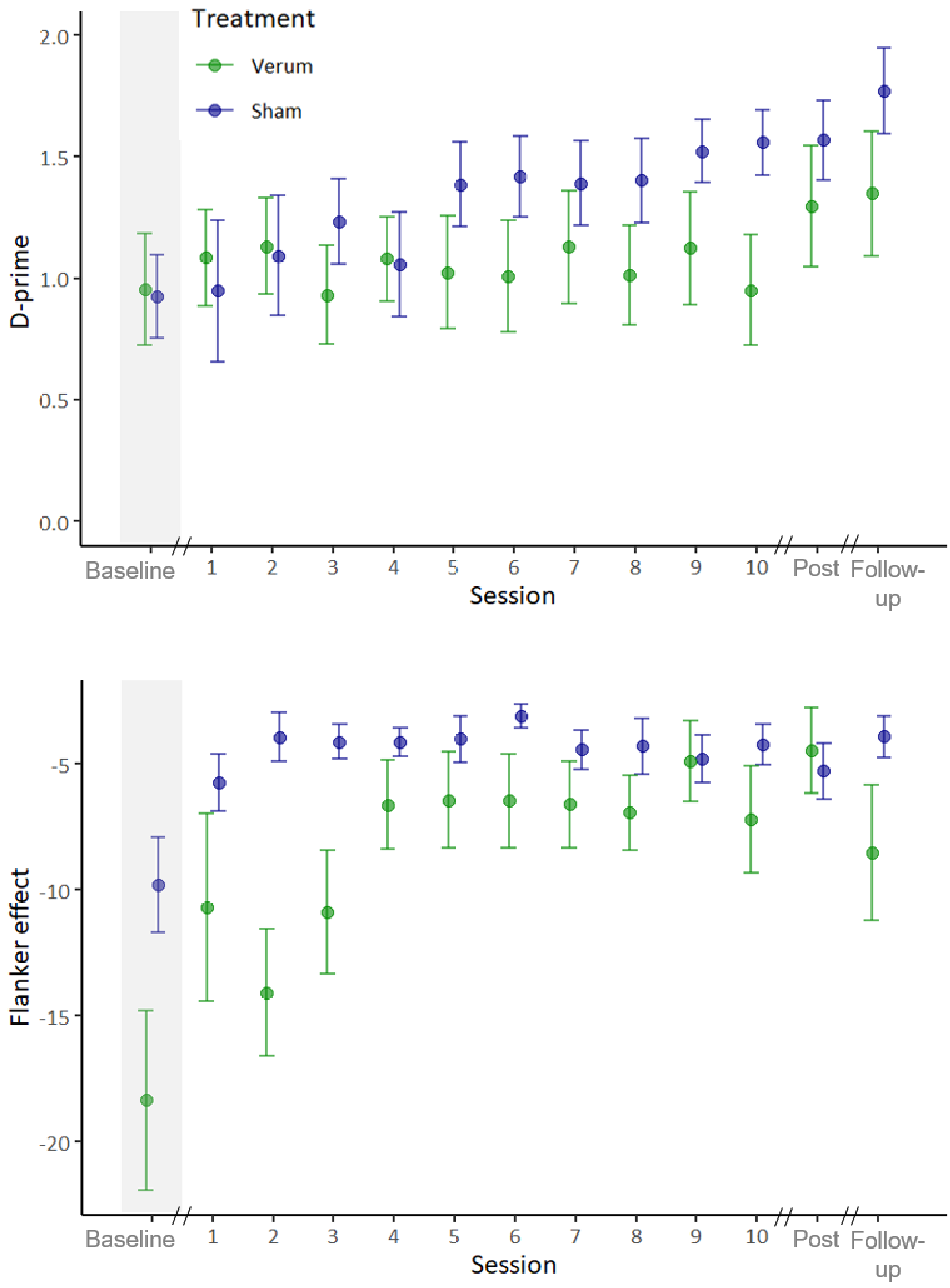

Raw means for working memory performance (d-prime) in study A and flanker effect accuracy in study B during tDCS intervention in sessions 1-10 and during EEG in pre-, post - and follow-up visits. Whiskers indicate standard errors (SEs). The grey bar marks the baseline measurement.

© 2025 Krauel K et al. *JAMA Network Open*.

eMethods 1. Participants

Contraindications for tDCS and therefore exclusion criteria for the study were past or present neurological diseases or brain surgery, dermatological diseases of the scalp, cochlear implants, a history of craniocerebral injury with loss of consciousness and heart disease.
At the screening visit non-verbal intelligence quotient (IQ) was measured with the Culture Fair Intelligence Test – Scale 20[1] at German sites and the performance IQ of the WISC-III[2] in Coimbra. In case IQ assessments using the CFT-20 R (Germany) or the WISC-III (Portugal) not older than a year were available these were obtained. In addition to the clinical outcomes, all sites assessed socioeconomic status of the parents/custodian[3], pubertal development[4], laterality (Edinburgh handedness inventory[5]) and social communication skills[6]. Moreover, all female participants took a urine pregnancy test during the screening.

eMethods 2. Sample size

Initial sample size planning was based on statistical testing principles, assuming a medium expected effect size of Cohen's d=0.6 for the primary outcome measures, similar to tDCS effects in ADHD in previous studies[7]. For a power of 80% (two-sided significance level α=0.05), this leads to a sample size of 45 patients per treatment arm (G*Power3.1). Assuming a 10% drop-out, the planned sample size was 100 randomized patients for each study (50 per arm), resulting in a total of 200 patients for studies A and B together. However, the main objective was the estimation of effect sizes, by calculating 95% confidence intervals (CI) for the difference between verum and sham in the primary outcome measures at post assessment. With the initially planned sample size of 100 patients per study, and an effect size of d=0.6, such a 95% CI was expected to have a width of 2*0.42 (standard deviation units, SDU). Due to delays in study conduct and difficulties in recruitment (among others due to the Sars-CoV2 pandemic), it became clear that this sample size could not be reached. It needs to be noted that the reached sample size of 34 (study A) or 31 (study B) patients available for analysis, only allowed for a broader, less precise CI (expected width of 95% CI: 2*0.72 SDU). Using up to 13 assessments of the primary outcomes (performance in target task, which was measured at baseline, at each intervention session and at post-intervention visit and follow-up visit) in a linear mixed model increases statistical precision (as evidenced by smaller 95% CIs) and power.

eMethods 3. Optimized montages

3.1 ***Montage optimization algorithm***

The optimization of the positions and currents of the electrode montage was performed using the Stimweaver algorithm[8]. Stimweaver is based on the premise that it is the normal component of the E-field normal to the cortical surface (E_n) that leads to the concurrent effects of stimulation on cortical excitability, as this component of the E-field optimally polarizes the soma of large pyramidal cells in the cortex[9, 10]. The inputs to Stimweaver are maps of the cortical surface containing information of the target E_n (E_n^{Trg}) field (positive/negative for a desired excitatory/inhibitory effect) and the weights (scalars, w, between 1 and 10 indicating the importance of each point for the optimization) associated to each node (vertex) i of the cortical surface mesh. The objective function of the minimization is the error with respect to no intervention ($ERNI$), defined as:

$$ERNI = \sum_{i=1}^{N_{mesh}} \frac{\left(w_i E_n^i - w_i E_n^{Target,i}\right)^2 - \left(w_i E_n^{Target,i}\right)^2}{\frac{1}{N_{mesh}} \sum_{j=1}^{N_{mesh}} w_j} \qquad (1)$$

where N_{mesh} is the number of mesh nodes. The solution is constrained by maximum current per electrode ($I_{Max} = \max_i \{I_i\}$, where $\{I_i\}$ is the set of all the currents for all the electrodes in the montage) and total injected current ($I_{Total} = \frac{1}{2}\sum_{i=1}^{N_{elecs}} |I_i|$). Furthermore, a genetic algorithm is employed to restrict the number of channels in the montage (usually a maximum of 6-8 channels is deemed adequate for most targets). The algorithm takes advantage of the linearity principle to generate the E-field distribution for any multichannel montage by linearly combining a pre-calculated set of lead vectors. These vectors contain the E_n for each mesh point in the cortical mesh induced by all possible bipolar combinations of the electrodes available with one common cathode (Cz, -1 mA). These calculations therefore consider the geometry and electrical properties of all head tissues represented in the computational head model used in the optimization.
The head model used for optimization was based on the Colin27 head model and the methods used to create the volume conductor model are described in[11]. In short the head model contains tissues representing the scalp, skull (representing both the spongy and compact bone), CSF (including the ventricles), grey-matter and white-matter. These were represented as homogeneous and isotropic tissues with electrical conductivities of 0.33 S/m, 0.008 S/m, 1.79 S/m, 0.40 S/m and 0.15 S/m, respectively. This model additionally contains models of Ag/AgCl PiStim electrodes (represented as 1 cm cylinders) placed in the 64 positions of Neuroelectric's headcap pro (subset of the 10/10 EEG system[12]).

© 2025 Krauel K et al. *JAMA Network Open*.

3.2 Study A optimization

For this optimization the target was defined as the left dorsolateral prefrontal cortex (lDLPFC), defined as Brodman area 46 in the left-hemisphere. The target E_n values in the lDLPFC region were set to +0.25 V/m and the weights to 10 (see figure S1, columns 2 and 3, respectively for the target E_n and target weights). In the rest of the cortical surface, the target E_n was set to 0.0 V/m with weights of 1. The current constraints were set to 0.5 mA max per electrode (in absolute value) and 1.0 mA max total injected current. Solutions with a maximum of 2, 4, 6 and 8 electrodes were tested. The 4 electrodes solution was found to be a good compromise between simplicity of set-up, average E_n on target and fitness value (ERNI). The montage employs electrodes F3, AF3 (anodes, with a current of 0.5 mA) and TP7, Oz (cathodes, with a current of -0.5 mA). The distribution of the E_n-field induced by the optimized montage is shown in figure S1 (1st column), and a summary of the more relevant figures of merit of the montage are shown in table S2.

Table S1

Average, max and min values of En as well as ERNI scores for the region defined in the target map.

Solution	Goodness of fit metrics	Target to stimulate		
	ERNI (mV^2/m^2)	Avg. E_n (V/m)	Max E_n (V/m)	Min E_n (V/m)
**Montage (4 channel)**	-1.1025×10^4	0.060	0.225	-0.093

© 2025 Krauel K et al. *JAMA Network Open*.

Figure S1
Optimized montage, IMax = 0.5mA/, 4 electrodes montage.

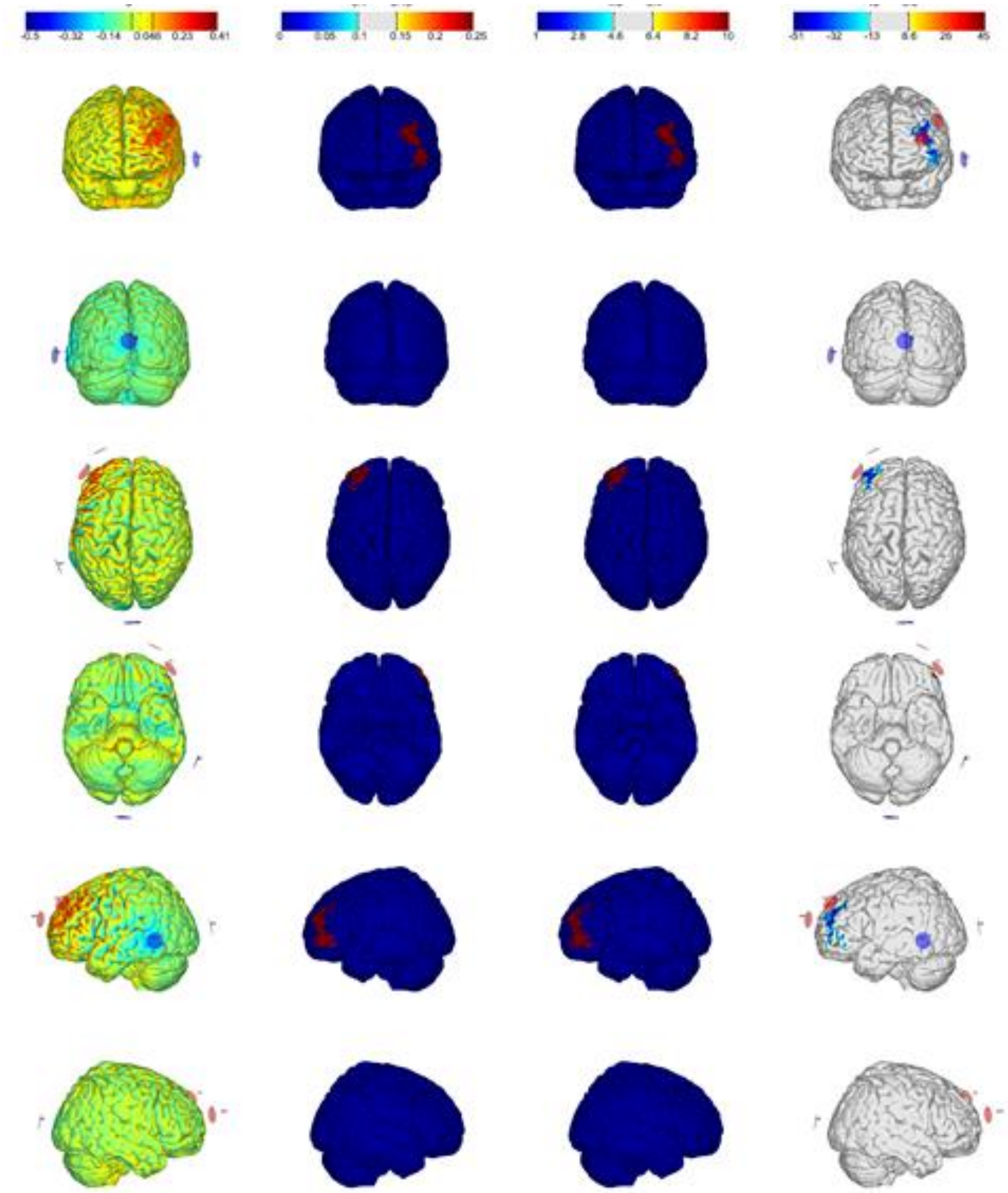

From Left to right: Normal component of the E-field En (V/m), target E-field (V/m), target weight and ERNI[13] (mV2/m2) for grey matter.

3.3 Study B optimization
The rIFG target was defined as BA 44, 45 and 47 (right hemisphere), with the pars triangularis region manually added. These regions were assigned to a positive target En-field (+0.25 V/m) with maximum weight (10). The rest of the right hemisphere, and the frontal cortex of the left hemisphere were assigned to a no-stimulation condition with weight 4. Finally, the equivalent to rIFG in the left hemisphere was assigned to a no-stimulation condition (target E_n set to 0.0 V/m) with maximum weight (10). The target E_n and weights map are shown in figure S2, columns 2 and 3, respectively. The current constraints and maximum number of channels were set to the same values as mentioned in the previous section. As before, the 4 electrodes solution was found to be a good compromise between simplicity of set-up, average E_n on target and fitness value (ERNI). The montage employs electrodes F6, F8 (anodes, with a current of 0.5 mA) and AFz, P7 (cathodes, with a current of -0.5 mA). The distribution of the E_n-field induced by the optimized montage is shown in figure S2 (1st column), and a summary of the more relevant figures of merit of the montage are shown in table S3.

© 2025 Krauel K et al. *JAMA Network Open*.

Figure S2
Optimized montage, IMax = 0.5mA/, 4 electrodes montage.

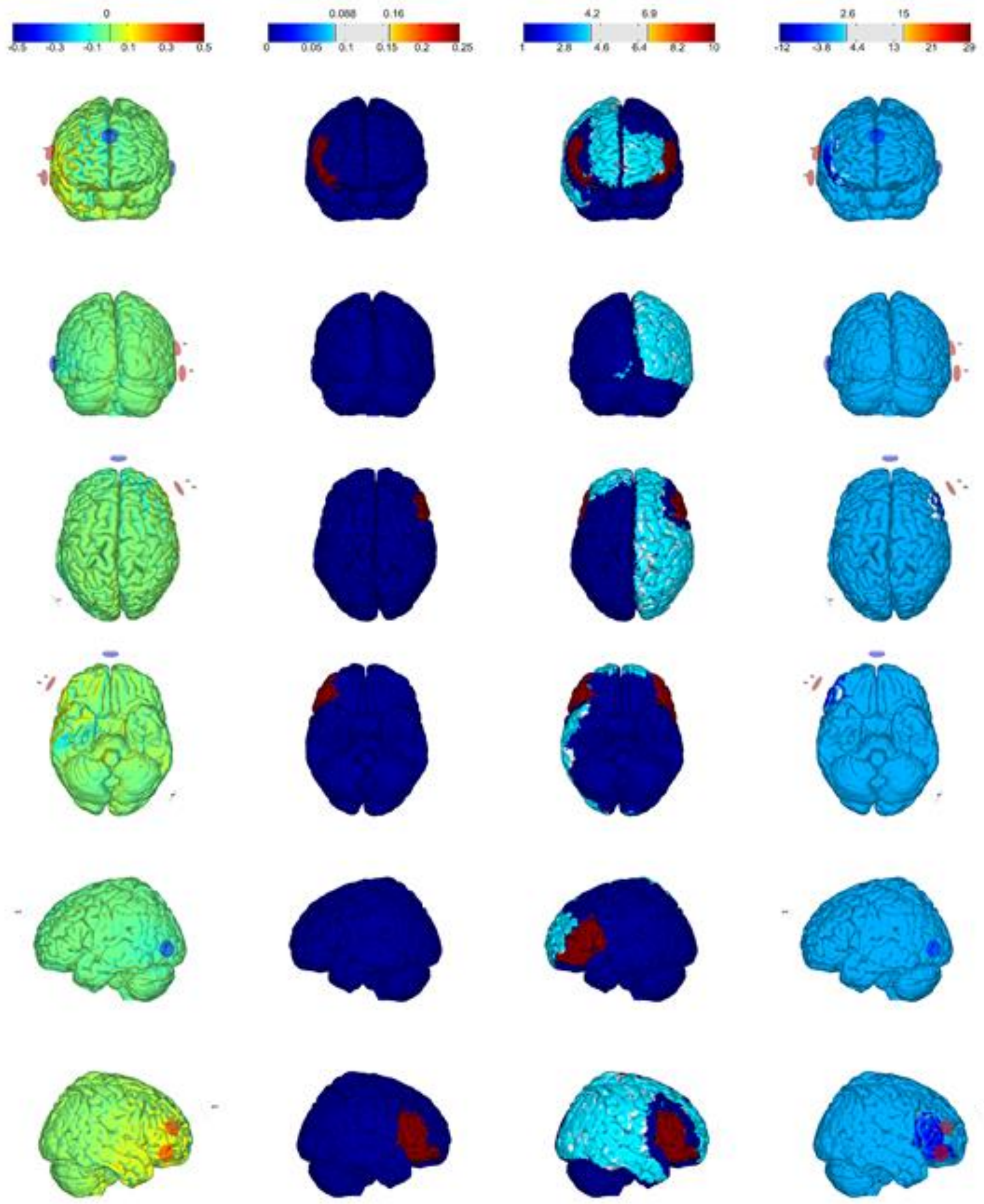

From Left to right: Normal component of the E-field En (V/m), target E-field (V/m), target weight and ERNI[13] (mV2/m2) for grey matter.

© 2025 Krauel K et al. *JAMA Network Open*.

Table S2
Goodness of fit metrics for each montage in the region defined in the target map.

Solution	Goodness of fit metrics	Target to stimulate		
	ERNI (mV^2/m^2)	Avg. E_n (V/m)	Max E_n (V/m)	Min E_n (V/m)
**Montage (4 channel)**	-3.860×10^3	0.038	0.28	-0.21

eMethods 4. Cognitive tasks

During tDCS and as primary endpoint for working memory performance (Study A) we used the 2-back task[14, 15], for interference control (Study B) the Flanker task[16, 17] was applied – both tasks are standard paradigms in assessing neurocognitive deficits in ADHD and have been employed in our previous studies[18, 7]. To assess transfer or adverse effects, we included the continuous performance task A-X which is widely used in ADHD research (CPT A-X) and measures attentional and inhibitory control[19]. Task performance was assessed during each intervention session (accuracy, commission and omission errors, reaction time and reaction time variability, Study A: n-back task, Study B: Flanker task).
Cognitive tasks were implemented using Presentation® software (Version 20.0, Neurobehavioral Systems, Inc., Berkeley, CA, www.neurobs.com) and performance measures were calculated via Matlab (The MathWorks Inc., 2020b) and R version 4.2.3 (R Foundation) in a blinded manner.

4.1 N-back task

We used the n-back task (Figure S3) with n=2. In the n-back task a series of pictures are presented, with 30% of the n-back trials being target trials, which means that the currently presented stimulus is identical to the stimulus presented two trials earlier (2-back task). Thus, the task requires continuous processes of encoding, retrieving and updating contents of working memory. Participants were instructed to decide by mouse button press with the forefinger and middle finger of their right hand if the current stimulus is a target or not. In order to make the task more difficult, some pictures were similar but not identical, and therefore not considered a target (for example two different bicycles). The presented objects in each block consisted of 10 different photographs that were equally often presented as non-targets served as a target twice. For each trial the stimulus was presented for 500 ms and afterwards the fixation cross was presented for 1750 ms (jittered between 2092ms and 2500ms) resulting in a mean duration of 2250 ms for one trial. During the 10 intervention sessions in study A the n-back task was presented for three blocks (5 min per run) with 1 min. pauses in between and a 2.5min interval at the beginning and the end without any task. During baseline , post-stimulation and follow up assessment, the task was presented for two blocks with a short practice at the beginning and with an 1 min. break in between.
Task performance (accuracy, commission and omission errors, reaction time and reaction time variability) was assessed during baseline, post, and follow-up assessment in both studies and also during intervention sessions in study A. Only the first reaction after the stimulus was considered. Trials were removed from analyses, if the reaction time was below 100 ms. The maximum time window to reply in this task was 2250 ms, which means a trial was considered no reaction, if the reaction time was more than 2250 ms.
Possible reactions in the n-back task are the correct identification of a target (hit) and a non-target (correct rejection) and the incorrect reaction to a target (miss) and non-target (false alarm). The sensitivity measure d-prime (d') was calculated via the formula: *d' = z-transformed rate of correct identifications of a target (hit rate) minus z-transformed rate of incorrect reactions to a non-target (false alarm)*. Hautus correction was applied to address extreme false-alarm and hit proportions[20].

© 2025 Krauel K et al. *JAMA Network Open*.

Figure S3
Schematic representation of the n-back working memory task.

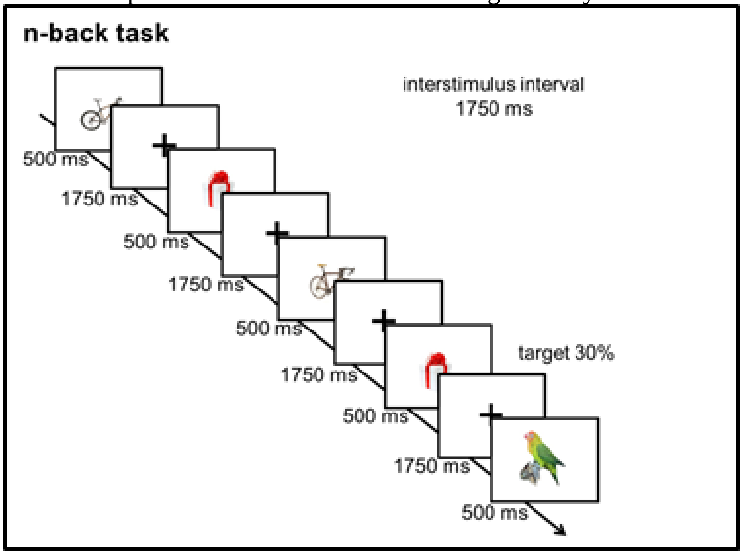

4.2 Flanker task
In the flanker task (Figure S4), stimuli consisted of five arrows. The middle arrow was the target stimulus and participants should indicate via button press on a keyboard with the forefinger right or with the left hand if the target arrow points to the right or to the left. The outer arrows served as distractors and can point in the same direction (congruent trial) or in the opposite direction (incongruent trial). Each block consisted of 184 trials and had a duration of 4.5 min, with 50% of the trials being congruent and incongruent. Stimuli were presented for 60 ms and participants had 1360 ms to respond. The interstimulus interval varies between 1560 to 1860 ms. During the 10 intervention sessions in study B the flanker task was presented for three blocks (5 min per run) with 1 min. pauses in between and a 2.5 min interval at the beginning and the end without any task. During baseline, post-stimulation and follow-up assessment, the task was be presented for two blocks with a short practice at the beginning and with a 1 min. break in between.
Task performance (accuracy, commission and omission errors, reaction time and reaction time variability) was assessed during baseline, post, and follow-up assessment in both studies and also during intervention sessions in study B. Only the first reaction after the stimulus was considered. Trials were removed from analyses, if the reaction time was below 100 ms. The maximum time window to reply in this task was 1660 ms, which means a trial was considered an omission error, if the reaction time exceeded 1660 ms.
The flanker effect was calculated via the formula: *percent correct responses in incongruent trials minus percent correct responses in congruent trials*.

Figure S4
Schematic representation of the flanker task.

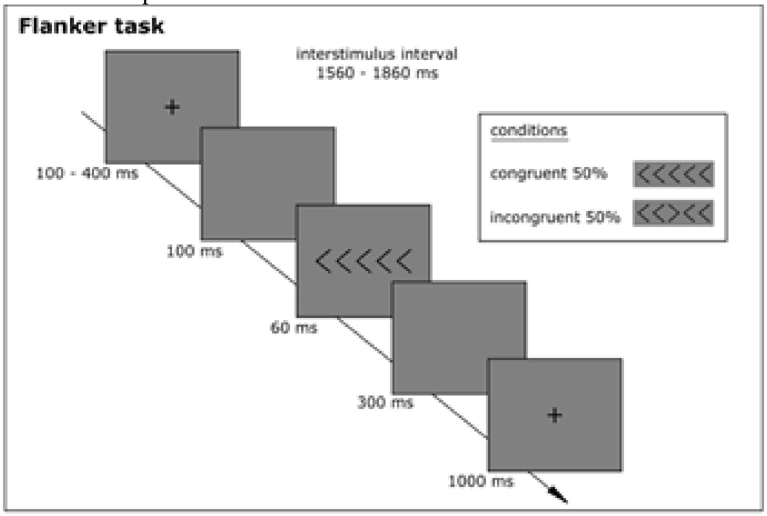

© 2025 Krauel K et al. *JAMA Network Open*.

4.3 Continuous performance task

In the continuous performance task (CPT) (Figure S5), various upper-case letters were presented. One of the letters was defined as a cue and one as a target stimulus. Whenever the cue was followed by the target stimulus, participants were instructed to press a button. However, participants should withhold their response if the cue was followed by another stimulus. The task consisted of 2 runs á 4.5 min (80 stimulus sequences per run, 20 target sequences, 20 non-target sequences) a short practice at the beginning and with a 1 min break in between. For each trial the stimulus was presented for 150 ms and participants had 1400 ms to give their response (interstimulus interval = 1650 ms). The CPT was administered during baseline, post-stimulation and follow-up assessments.

Task performance (accuracy, commission and omission errors, reaction time and reaction time variability) was assessed during baseline, post, and follow-up assessment in both studies. Only the first reaction after the stimulus was considered. Trials were removed from analyses, if the reaction time was below 100 ms. The maximum reaction time was 1550 ms, which means a trial was considered a miss, if the reaction time exceeded 1550 ms.

Figure S5

Schematic representation of the continuous performance task.

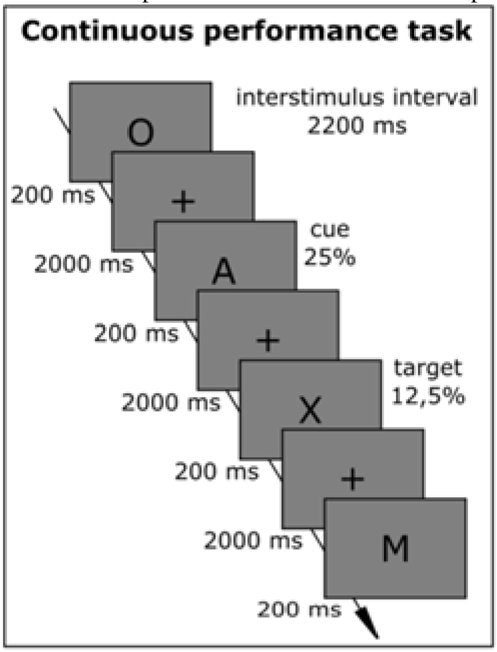

© 2025 Krauel K et al. *JAMA Network Open*.

eMethods 5. Randomization / sequence generation / allocation concealment

Randomization was stratified by center (5 centers) and sex and blocked with a block size of four. Allocation sequences (separately by center and sex) fulfilling these criteria were generated using computer-generated random numbers as implemented in the BiAS software (https://www.bias-online.de/). Randomization lists containing these random allocation sequences with the assignment both to study A or study B and the respective study arm (sham or verum) were prepared by the Institute of Medical Informatics and Statistics (IMIS) Kiel.
The device Starstim32 (Neuroelectrics SLU, Barcelona, Spain) allows to perform double-blind stimulation. Prior to study start, an administrator at Neuroelectrics created templates for verum and sham stimulation separately for studies A and B to be used in the stimulation software, Neuroelectrics® Instrument Controller (NIC2). A sufficiently large number of identical copies of the four different basic types of stimulation setting files (for A and B, sham and verum tDCS each) were prepared, individually named (using randomly generated file names without any reference to the content) and distributed to the study centers. The assigned intervention was associated with these prepared file names. Participants were recruited at the study centers and after definite inclusion of a subject into the study (signed informed consent, fulfilment of all inclusion criteria), allocation to one study arm was performed via the the electronic Case Report Form (eCRF) system by revealing the file name of the stimulation setting file to be used for the specific participant. Thus allocation was concealed until definite inclusion into the study. Allocation to study A or B (but not sham or verum) were revealed to study personnel, as different concurrent target tasks were performed during stimulation.

eMethods 6. Blinding

6.1 Blinding

During the stimulation session, the study personnel had no access to the password-protected content of this file (protocol data, i.e. current intensity, sham-settings) and thus remained effectively blinded. Patients, parents, care providers and all those involved in administering the intervention or assessing outcomes were blind to the stimulation condition (verum vs. sham) but not to the study A or B (i.e. stimulated brain region and associated neuropsychological task). Blinding was maintained for the whole duration of the trial.

6.2 Unblinding in emergencies

Unblinding would have been possible in case of the following emergencies: reasons of subjects' safety and decisions on further medical treatment. Such an unblinding would have been reported to authorities and ethics committees as a serious adverse device effect (SADE). Unblinding was possible through emergency envelopes on site, which contain information on sham or real stimulation for each of the distributed files (based on file name). The participant and his/her parents needed to be informed about unblinding. Unblinding would have led to the exclusion of the respective participant from further intervention sessions. No unblinding actually happened during the study.

eMethods 7. Statistical methods

In order to follow the intention-to-treat principle as closely as possible, all participants were asked to participate in the post-intervention and follow-up assessment, even if they prematurely discontinued treatment, to minimize the amount of missing data. In addition, the primary outcome variable (cognitive task performance) was measured at every stimulation visit, so that any participant who actually started the randomised intervention and had at least one stimulation session would also have at least one value of the primary outcome variable available. Thus, all participants who actually received at least one stimulation were included in the modified intention-to-treat set in their originally assigned group. However, patients for whom no outcome data at all is available (in particular no value of the primary outcome measure except from the baseline visit), do not contribute any information regarding treatment effects and were thus not included in the statistical analysis. This only occurred for a total of 3 randomised participants, who did not receive even one stimulation (1 participant in Study A sham arm, 1 participant in Study B sham arm and participant in Study B verum arm, see CONSORT flow chart, figure 1). Before analysis, a blinded review of all visit documentation notes was performed to assess relevant protocol violations (in particular regarding stimulation duration and conditions). Additionally, notes about neuropsychological testing conditions were assessed (also blinded) to determine whether these were critically compromised so that data could not be included in any further analysis (e.g. technical issues with task administration). Neuropsychological testing data was collected in the task presentation software. Performance measures of all cognitive tasks were preprocessed for each individual in a blinded manner at ZIP Kiel and OVGU Magdeburg and aggregated in data files (see eMethods 3).
As a deviation from the original statistical analysis plan, we used linear mixed models instead of ANCOVAs for the statistical analyses of primary and secondary outcomes, since this allowed inclusion of all outcome measurements to achieve a higher statistical precision compared to ANCOVA. However,

© 2025 Krauel K et al. *JAMA Network Open*.

both ANCOVA using only the outcome value at the post-intervention visit as a dependent variable with multiple imputation (MI) for missing values and the linear mixed model of all outcome measurements without any imputation are valid under the missing at random (MAR) assumption. Specification of the MI model would have included exactly those baseline covariates that were also used in the linear mixed model, thus there would be no relevant difference in the influence of missing data between the two models. Thus, the main difference between these models lies in the fact that we used all available measurements of primary (or secondary) outcome variables in the linear mixed model which results in higher power and statistical precision than using just one outcome measurement per patient in the ANCOVA. The primary cognitive task performances (i.e. the primary outcome variables) were measured during and after the intervention (all stimulation visits, post-intervention visit and follow-up visit) i.e. up to 12 measurements per patient, while secondary outcomes were mostly measured at post-intervention visit and follow-up visit. This improved precision of the linear mixed model was particularly relevant since it was not possible to recruit the planned sample size.

Following Molenberghs[21] et al. (2004), the linear mixed effects model for primary and secondary outcomes models the correlation within the repeated outcome measurements over time by specifying correlated residual errors. In particular, to reduce the chances of model misspecification, the residual errors are assumed to be from a multivariate normal distribution with an unstructured covariance matrix. This imposes no restriction on the form of the correlation matrix for the vector of repeated measurements for each patient. This model was fitted. using restricted maximum likelihood (REML).

The randomised intervention arm and time were used as fixed effects, generally in a parsimonious model without time*intervention interaction to allow for better precision of effect estimates with the limited sample size available. A time*intervention interaction was included into the model only where p-values were < 0.15 for the interaction term of at least one measure of an outcome (e.g. subscales of the ADHD rating scale), which implies separate mean parameters for each time point in each treatment group. For all secondary outcomes with only two post-intervention measurement time points, time was used as a categorical variable, while time was used as a quantitative variable for the primary outcomes with up to 12 measurements per patient again to include fewer parameters in the model, and since at least monotone effects of time are plausible with this study design. Residual diagnostic plots were checked to assess model fit, including the linearity assumption in the time variable.

Further baseline covariates were adjusted for by inclusion as fixed effects as well; these were age, sex, IQ and the baseline measurement of the outcome variables. Since site was used in the stratified randomisation, we included site in the specification of the unstructured covariance matrix, with patient ID nested in site. Inter-individual differences in any other variables at baseline (e.g. in ADHD presentation and comorbidities) are most relevant for the outcomes if they are related to differences in e.g. baseline cognitive task performance and are thus not included in the model.

Estimates and confidence intervals for the intervention effect at each visit, were constructed using linear combinations based on the coefficients in the model (R package multcomp). Estimated marginal means (on the original scale of measurement) were calculated using the R package emmeans and are presented for male sex and the sample means of all quantitative baseline covariates (age, IQ and baseline value of the outcome measure). Standardized effect sizes were calculated as estimated difference in outcome between intervention arms (based on emmeans) divided by the estimated population standard deviation.

In line with the primary objective of estimating effect sizes, results are reported as effect sizes with 95% confidence intervals. In particular for all secondary outcomes, p-values are only reported for completeness but are not corrected for multiple testing and should be interpreted cautiously.

eResults. Recruitment

Patients were included intermittently between September 2018 (first patient, first visit) and October 2021 (last patient, last follow-up visit) at five university hospitals: (1) the ICNAS, Clinical and Academic Center, University of Coimbra, Portugal, (2) the Department of Child and Adolescent Psychiatry, Psychosomatics and Psychotherapy at the University Hospital Frankfurt, Goethe University, Frankfurt am Main, Germany, (3) Evangelisches Klinikum Bethel, Bielefeld, Germany (4) the Department of Child and Adolescent Psychiatry and Psychotherapy, Zentrum für Integrative Psychiatrie, Kiel, Germany, and (5) the Department of Child and Adolescent Psychiatry and Psychotherapy, Medical Faculty, Otto-von-Guericke-University Magdeburg, Germany. Families were recruited via approaching the patient pool of these clinics (out-patients and in-patients), locally distributed information (e.g. newspaper, postcards, leaflets), patient organizations and the STIPED homepage. Recruitment was supposed to begin in June 2018 but was immediately halted by two State Authorities (Schleswig-Holstein, Hessen). They voiced concerns that the risk classification IIa normally used for stimulation devices would not be appropriate when a) the brain is targeted and b) stimulation is applied in minors, and should therefore be raised to IIb. The German National Authority re-evaluated our protocol as well as the device and did not support the proposed change in risk classification. The recruitment stop was revocated and inclusion of patients could start in September 2018. Inspections by State Authorities were then conducted in Kiel (ZIP, study site inspection, December 5th 2018), Frankfurt (GU, study site inspection, November 20th

© 2025 Krauel K et al. *JAMA Network Open*.

2019), and Magdeburg (OvGU, sponsor inspection, December 4th 2019). The last inspection resulted in another recruitment stop starting on February, 14th 2020 for all sites. The inspector requested explicit information on the sham treatment in the protocol and the patient information as well as an updated risk analysis and a German manual for the stimulation device. In close cooperation with Neuroelectrics, the required information was provided as requested by March 13th, 2020 and was approved by June 3rd, 2020. Then, these changes had to be submitted as an amendment to the National Authority and leading ethic committee in Germany and were finally approved along with the prolongation of the study on October 14th. On January 5th 2021, all relevant material was submitted to the National Authorities in Portugal (INFARMED, CEIC) and we received the final approval on April 26th, 2021. The trial was ended before the planned sample size could be reached as funding resources did not allow further recruitment beyond October 2021.

eReferences.

1. Weiß RH. *Grundintelligenztest Skala 2—Revision (CFT 20-R) [Culture Fair Intelligence Test 20-R—Scale 2];* 2006.
2. Wechsler D. Wechsler Intelligence Scale for Children (3rd ed.). (WISC-III). Manual. 1991.
3. Lampert T, Müters S, Stolzenberg H, Kroll LE. Messung des sozioökonomischen Status in der KiGGS-Studie : Erste Folgebefragung (KiGGS Welle 1). *Bundesgesundheitsblatt Gesundheitsforschung Gesundheitsschutz.* 2014;57(7):762–770. doi:10.1007/s00103-014-1974-8.
4. Watzlawik M. Die Erfassung des Pubertätsstatus anhand der Pubertal Development Scale. *Diagnostica.* 2009;55(1):55–65. doi:10.1026/0012-1924.55.1.55.
5. Oldfield RC. The assessment and analysis of handedness: the Edinburgh inventory. *Neuropsychologia.* 1971;9(1):97–113. doi:10.1016/0028-3932(71)90067-4.
6. Rutter M, Bailey A, Lord C. The Social Communication Questionnaire. 2003.
7. Breitling C, Zaehle T, Dannhauer M, et al. Improving Interference Control in ADHD Patients with Transcranial Direct Current Stimulation (tDCS). *Front Cell Neurosci.* 2016;10:72. doi:10.3389/fncel.2016.00072.
8. Ruffini G, Wendling F, Merlet I, et al. Transcranial current brain stimulation (tCS): models and technologies. *IEEE transactions on neural systems and rehabilitation engineering : a publication of the IEEE Engineering in Medicine and Biology Society.* 2013;21(3):333–345. doi:10.1109/TNSRE.2012.2200046.
9. Rahman A, Reato D, Arlotti M, et al. Cellular effects of acute direct current stimulation: somatic and synaptic terminal effects. *The Journal of Physiology.* 2013;591(10):2563–2578. doi:10.1113/jphysiol.2012.247171.
10. Roth BJ. Mechanisms for electrical stimulation of excitable tissue. *Crit Rev Biomed Eng.* 1994;22(3-4):253–305.
11. Miranda PC, Mekonnen A, Salvador R, Ruffini G. The electric field in the cortex during transcranial current stimulation. *Neuroimage.* 2013;70:48–58. doi:10.1016/j.neuroimage.2012.12.034.
12. Jurcak V, Tsuzuki D, Dan I. 10/20, 10/10, and 10/5 systems revisited: their validity as relative head-surface-based positioning systems. *Neuroimage.* 2007;34(4):1600–1611. doi:10.1016/j.neuroimage.2006.09.024.
13. Ruffini G, Fox MD, Ripolles O, Miranda PC, Pascual-Leone A. Optimization of multifocal transcranial current stimulation for weighted cortical pattern targeting from realistic modeling of electric fields. *Neuroimage.* 2014;89:216–225. doi:10.1016/j.neuroimage.2013.12.002.
14. Jaeggi SM, Buschkuehl M, Perrig WJ, Meier B. The concurrent validity of the N-back task as a working memory measure. *Memory.* 2010;18(4):394–412. doi:10.1080/09658211003702171.
15. Redick TS, Lindsey DRB. Complex span and n-back measures of working memory: a meta-analysis. *Psychon Bull Rev.* 2013;20(6):1102–1113. doi:10.3758/s13423-013-0453-9.
16. Eriksen BA, Eriksen CW. Effects of noise letters upon the identification of a target letter in a nonsearch task. *Perception & Psychophysics.* 1974;16(1):143–149. doi:10.3758/BF03203267.
17. Wöstmann NM, Aichert DS, Costa A, Rubia K, Möller H-J, Ettinger U. Reliability and plasticity of response inhibition and interference control. *Brain Cogn.* 2013;81(1):82–94. doi:10.1016/j.bandc.2012.09.010.
18. Splittgerber M, Salvador R, Brauer H, et al. Individual Baseline Performance and Electrode Montage Impact on the Effects of Anodal tDCS Over the Left Dorsolateral Prefrontal Cortex. *Front Hum Neurosci.* 2020;14:349. doi:10.3389/fnhum.2020.00349.
19. Albrecht B, Brandeis D, Uebel H, et al. Familiality of neural preparation and response control in childhood attention deficit-hyperactivity disorder. *Psychol Med.* 2013;43(9):1997–2011. doi:10.1017/S003329171200270X.
20. Hautus MJ. Corrections for extreme proportions and their biasing effects on estimated values ofd′. *Behavior Research Methods, Instruments, & Computers.* 1995;27(1):46–51. doi:10.3758/BF03203619.
21. Molenberghs G, Thijs H, Jansen I, et al. Analyzing incomplete longitudinal clinical trial data. *Biostatistics.* 2004;5(3):445–464. doi:10.1093/biostatistics/5.3.445.

© 2025 Krauel K et al. *JAMA Network Open*.
