# Supplementary material for: Prefrontal Transcranial Direct Current Stimulation in Pediatric Attention-Deficit/Hyperactivity Disorder: A Randomized Clinical Trial
Source: JAMA Netw Open. 2025 Feb 21;8(2):e2460477. doi: 10.1001/jamanetworkopen.2024.60477 (PMC11846015; doi:10.1001/jamanetworkopen.2024.60477)
Supplement: Supplement 3. — Data Sharing Statement [file jamanetwopen-e2460477-s003.pdf]

Data Sharing Statement

Krauel. Prefrontal Transcranial Direct Current Stimulation In Pediatric Attention-Deficit/Hyperactivity Disorder. *JAMA Netw Open*. Published February 21, 2025. doi:10.1001/jamanetworkopen.2024.60477

Data

Additional Information: The Sponsor applied for and received a Universal Trial Number (U1111-1199-0145) and registered E-StimADHD in the German Clinical Trials Register (Deutsches Register Klinischer Studien, DRKS). The DRKS is an approved primary register in the WHO network.trial protocol: https://drks.de/search/en/trial/DRKS00012659. Trial Registration: EUDAMED-Nr. CIV-17-09-021424;
Data available: Yes
Data types: Data dictionary, Deidentified participant data
How to access data: Deidentified participant data are available from the corresponding author Kerstin Krauel (kerstin.krauel@med.ovgu.de) upon reasonable request.
When available: With publication

Supporting Documents

Document types: Other (please specify)
Additional Information: Data dictionary
How to access documents: A data dictionary will be available from the corresponding author Kerstin Krauel (kerstin.krauel@med.ovgu.de).
When available: With publication

Additional Information

Who can access the data: Researchers whose proposed use of the data has been approved.
Types of analyses: The data will be made available for a specified purpose.
Mechanisms of data availability: The data will be made available after approval of a proposal.
Any additional restrictions: none
